# Comparison of Human Metapneumovirus- and Respiratory Syncytial Virus–Associated Health Burden in Adults With Chronic Underlying Health Conditions: A Global Systematic Review and Meta-analysis

**DOI:** 10.1093/ofid/ofag343

**Published:** 2026-06-08

**Authors:** Durga Kulkarni, Bohee Lee, Amelie Kerry, Kate Templeton, Harish Nair

**Affiliations:** Centre for Global Health, Usher Institute, University of Edinburgh, Edinburgh, UK; National Heart and Lung Institute, Imperial College London, London, UK; Deanery of Biomedical Sciences, University of Edinburgh, Edinburgh, UK; Viral Genotyping Reference Laboratory Edinburgh, NHS Lothian, Royal Infirmary of Edinburgh, Edinburgh, UK; Centre for Global Health, Usher Institute, University of Edinburgh, Edinburgh, UK; School of Public Health, Nanjing Medical University, Nanjing, China; MRC/Wits Rural Public Health and Health Transitions Research Unit (Agincourt), School of Public Health, Faculty of Health Sciences, University of the Witwatersrand, Johannesburg, South Africa

**Keywords:** comorbidity, hospital burden, respiratory infections, viruses

## Abstract

**Background:**

We aimed to systematically compare globally the health burden associated with human metapneumovirus (hMPV) and respiratory syncytial virus (RSV) respiratory tract infections in adults with chronic illnesses.

**Methods:**

We searched MEDLINE, EMBASE, Global Health, CINAHL, Web of Science, and Global Index Medicus on 18 August 2025 and 12 January 2026. We included observational and interventional studies conducted from 2001 onwards, reporting laboratory-confirmed hMPV and RSV infections in adults with chronic illnesses. Quality assessment was conducted using the Joanna Briggs Institute critical appraisal tools. We developed an overall test positivity estimate, using a generalized linear mixed-effects model with a binomial distribution and logit link, including a study-level random intercept and fixed effects for virus, setting, and their interaction. Predicted probabilities and population-average odds ratios (ORs) comparing RSV and hMPV were calculated. Incidence and disease severity outcomes were synthesized narratively due to heterogeneous and limited data. The protocol was registered on Open Science Framework (doi:10.17605/OSF.IO/EPB3X).

**Results:**

Fifty studies, reporting on hMPV and RSV test positivity (n = 44; 29,603 laboratory tests), incidence (n = 3), and disease severity outcomes (n = 7), were included. The OR of RSV test positivity versus hMPV in adults with at least 1 chronic illness was 1.88 (95% confidence interval: 1.61–2.19) in inpatient settings and 1.65 (1.43–1.91) across all settings in studies relying on annual data. Significant differences in RSV versus hMPV test positivity were found for chronic respiratory disease (annual and seasonal analyses), cardiovascular (seasonal), immunocompromised (annual), and hematologic (annual) conditions but not among solid organ transplant recipients (annual). Incidence and severity data demonstrated inconsistent patterns.

**Conclusions:**

Respiratory syncytial virus occurs more frequently than hMPV in adults with chronic illnesses. However, evidence gaps remain regarding comparative disease severity and data from low- and middle-income countries.

Diagnostic and treatment advances have substantially improved the survival of patients with chronic underlying health conditions, including cancer and cardiovascular disease. This has led to “morbidity expansion,” characterized by a rising number of individuals with chronic illnesses and multimorbidity, particularly in younger age groups [1]. Consequently, a growing proportion of the population is susceptible to infections.

Respiratory tract infections (RTIs) associated with human influenza are well studied in populations with underlying health conditions to inform vaccine policies. However, similar epidemiological data for other respiratory viruses, like human metapneumovirus (hMPV) and respiratory syncytial virus (RSV), remain limited. Individuals with chronic conditions are at increased risk of hMPV and RSV infections and severe outcomes, with high healthcare resource utilization [2]. Multiple RSV vaccines for older adults and immunocompromised individuals have been recently licensed and are becoming increasingly available [3]. Additionally, combination vaccines targeting both hMPV and RSV are currently undergoing testing in clinical trials [4, 5].

A systematic review estimated that hMPV accounted for 4.3% (95% confidence interval [CI]: 3.2%–5.7%) of annual and 5.1% (95% CI: 3.2%–7.9%) of seasonal symptomatic respiratory infections in adults with chronic conditions in developed countries [6]. We previously estimated the annual and seasonal RSV-associated acute respiratory infection (ARI) incidence rates of 37.6 (95% CI: 20.1–70.3) and 28.4 (95% CI: 11.4–70.9) per 1000 adults with chronic conditions, respectively, in industrialized countries [7]. However, systematic comparisons of the disease burden associated with hMPV and RSV in adults with underlying health conditions, based on data from the same studies, are currently unavailable.

Therefore, we aimed to systematically estimate and compare globally the disease burden associated with hMPV- and RSV-positive RTIs in adults with chronic underlying health conditions. These findings will help inform vaccination policies and resource planning for high-risk populations, including the use of combination vaccinations when licensed.

## METHODS

We followed the Preferred Reporting Items for Systematic Review and Meta-Analysis Protocols (PRISMA-2020) guidelines [8]. The review protocol was registered on the Open Science Framework [9]. We completed the PRISMA 2020 checklist (Supplementary Table 1). Ethics approval was not required for this systematic review, as it was based exclusively on published data.

We searched 6 databases—MEDLINE (Ovid), Embase (Ovid), CINAHL (EBSCO), Global Health (Ovid), Global Health Medicus, and Web of Science Core Collection—on 18 August 2025 and updated the searches on 12 January 2026. A team member (D. K.) developed a comprehensive search strategy in MEDLINE, using terms related to hMPV, RSV, comorbidities, and disease burden outcomes. The search terms were tailored to individual databases. No language and regional filters were applied. Another team member (B. L.) peer-reviewed the search strategies [10]. The full search strategies are provided in Supplementary Table 2.

We included the following studies: using observational (cross-sectional, case–control, or cohort) or interventional study design (included data only from the placebo or control group); including laboratory-confirmed hMPV and RSV infections; providing data on hMPV and RSV proportion positive in RTI or acute exacerbation of chronic disease episodes or routine clinical testing regardless of symptoms (test positivity), hMPV and RSV incidence, or severity (hospitalization, intensive care unit [ICU] admission, deaths); including data 2001 onwards (as hMPV was first identified and isolated in 2001); providing data on adults (≥18 years); and having sample sizes of at least 100 episodes or specimens being tested (in the denominator) for the test positivity outcome. We included studies reporting data on “adults,” when they did not explicitly define the adult age range and assumed it to be ≥18 years. We did not restrict studies to the World Health Organization (WHO) case definitions of ARI or severe ARI, and we included all studies as long as the eligibility criteria for laboratory testing were clear [11, 12]. We included studies reporting at least 1 outcome in which both hMPV and RSV recorded nonzero values. Supplementary Text 1 provides the detailed inclusion and exclusion criteria.

Full-texts of non-English studies (reaching the full-text screening stage) were translated using Google Translate to assess eligibility.

A team member (D. K.) removed duplicate references first using EndNote 20 and then the COVIDence tool [13, 14]. Two reviewers (D. K., and B. L. or A. K.) independently screened titles, abstracts, and full texts of eligible studies in Covidence. Two reviewers (D. K. and B. L.) independently extracted summary data onto a pilot-tested MS Excel form (D. K.). Extracted study characteristics included first author, year, study setting and site, data collection period, data type (annual or seasonal), testing method and specimen, chronic underlying conditions, case definitions, and participant demographics. Outcomes included the total specimens or episodes tested, the number positive for hMPV and RSV, hMPV and RSV incidence, episodes requiring hospitalization or ICU admission, and deaths. Only outcomes with nonzero values for both viruses were extracted.

Two reviewers (D. K. and B. L.) independently conducted quality assessment of the included studies using the Joanna Briggs Institute (JBI) critical appraisal tools for each study design [15]. The JBI critical appraisal tools provided a structured critical appraisal of methodological quality and included domains relevant to risk of bias. Cohort studies with a score of 8 or higher (out of 11) and cross-sectional studies with a score of 7 or higher (out of 8) were deemed to be of good quality. Disagreements between the 2 reviewers at any stage were resolved through discussion and by involving an arbiter (H. N.).

### Data Analysis

All statistical analyses were conducted using R software version 4.2.1.

We conducted a meta-analysis to estimate hMPV and RSV test positivity in individuals with at least 1 underlying health condition. Analyses were stratified by study setting (inpatient, outpatient, and mixed) and restricted to studies with at least 1 complete year of data (annual). Mixed settings were defined as studies reporting combined inpatient and outpatient populations without separate stratified estimates. First, we applied a conventional random-effects meta-analysis, assessing heterogeneity using Tau^2^ and reporting *I*^2^ to indicate the proportion of variation due to heterogeneity. Second, we used a generalized linear mixed-effects model (GLMM) with a binomial distribution and link, including a random intercept for study, fixed effects for virus (RSV vs hMPV) and study setting, and a virus × setting interaction to allow the comparative effect of RSV versus hMPV to vary by setting. We assumed:


$$Y_{i}^{+}∼\mathrm{Binomial}(n_{i},p_{i}),$$

where $Y_{i}^{+}$and $Y_{i}^{-}$denote the number of virus-positive and virus-negative episodes, respectively, in the study *i*, and $n_{i}=Y_{i}^{+}+Y_{i}^{-}$.


$$\begin{array}{rl} \mathrm{logit}(\;p_{i}) & =\beta_{0}+\beta_{1}\mathrm{Viru}s_{i}+\beta_{2}\mathrm{Settin}g_{i}+\beta_{3}(\mathrm{Viru}s_{i}\times \mathrm{Settin}g_{i})\\ & \;+u_{\mathrm{Study}(i)}, \end{array}$$

where $u_{\mathrm{Study}(i)}∼N(0,\;\sigma_{\mathrm{Study}}^{2})$ represented a study-level random intercept.

We estimated the predicted probabilities of RSV and hMPV test positivity for the overall population by back-transforming the model's predicted log-odds to the probability scale with the corresponding 95% CIs, using the standard errors of the predicted log-odds. Setting-specific differences between RSV and hMPV were expressed as population-average odds ratios (ORs) with 95% CIs, obtained from the fixed-effect coefficients. Statistical significance was defined as *P* < .05. A pooled population-level estimate was also obtained from the mixed-effects model, constructed by weighting the setting-specific fixed-effect estimates by the proportion of observations in each setting.

Additionally, we performed a leave-one-out sensitivity analysis, sequentially removing each data point and recalculating the GLMM population-average ORs in each study setting. One study (1 data point) lacked information on the underlying disease before hematopoietic cell transplant (HCT) and was assumed to involve hematologic disease. The leave-one-out analysis also assessed sensitivity to excluding this study. Additional sensitivity analyses were conducted—one excluding the influential data points (those altering the OR by >10%), and another including only studies rated as good quality.

Moreover, we conducted a separate analysis using a similar GLMM but limiting the data to high-income countries (HICs). Countries were classified as high income if they were categorized as HIC by the World Bank country classification by income (according to the per capita gross national income as on 1 July 2025) [16].

We conducted a similar GLMM analysis for studies using seasonal data. Studies with mixed annual and seasonal data or from unclear settings were excluded from the meta-analysis. Additionally, 3 studies were excluded from the meta-analysis for population-specific reasons. One community-based study was excluded because only a single study was available in this setting, precluding GLMM analysis within the inpatient, outpatient, or mixed-care groups [17]. One study focused on asthma in pregnant women, and another investigated severe hospital-acquired pneumonia (HAP); both were excluded due to highly specific populations [18, 19]. Findings from these studies were summarized narratively.

Furthermore, we classified underlying health conditions into 11 broad groups (Supplementary Table 3). For each combination of comorbidity group and data collection period (annual or seasonal) with ≥3 studies, we performed a separate meta-analysis, using the same analytical method described above (Supplementary Table 4).

We could not pool the incidence data due to heterogeneity in chronic underlying health conditions, data collection periods, and study settings. We therefore presented incidence from individual studies and tested statistical differences between hMPV and RSV in individual studies, where 95% CIs were available.

Severity outcomes were analyzed narratively, as fewer than 3 studies reported hospital admissions. Although 3 studies reported ICU admissions, 2 included fewer than 50 RSV or hMPV cases, and RTI case definitions varied substantially (Supplementary Table 5). Differences in these definitions precluded meaningful pooling and limited the robustness of combined estimates. Mortality outcomes were heterogeneous (virus-attributable, in-hospital, 90-day overall), preventing meta-analysis.

### Role of the Funding Source

The funder had no role in study design, data collection, data analysis, data interpretation, or manuscript writing.

## RESULTS

**Figure 1.**
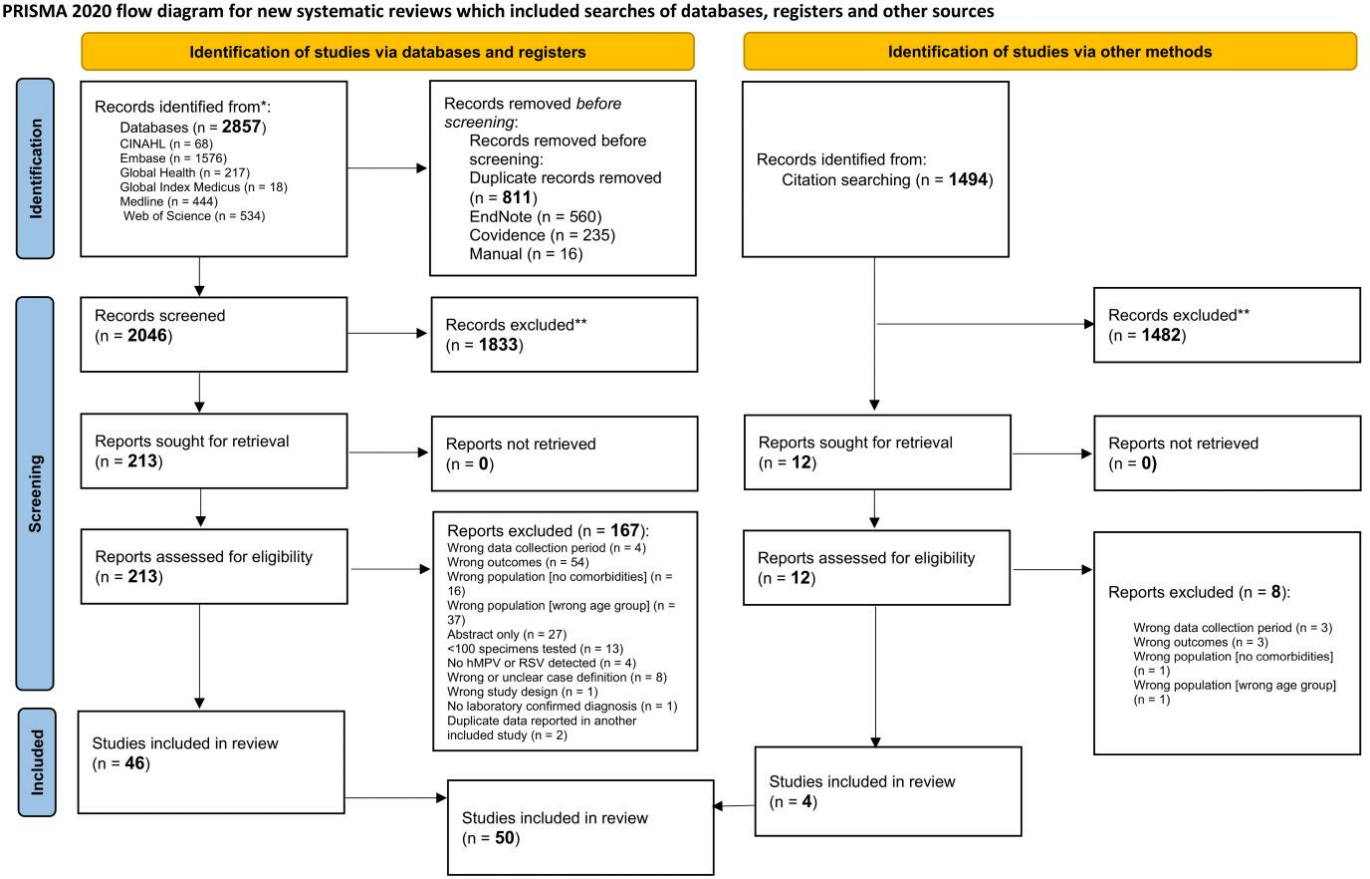
PRISMA flow chart.

Fifty studies were included in this systematic review [17–66]. Figure 1 demonstrates the flow of studies at each stage. Supplementary Table 6 provides the complete list of studies excluded at the full-text screening stage, along with reasons for exclusion. None of the studies published in languages other than English met the eligibility criteria.

Table 1 summarizes the characteristics of included studies. The studies were conducted between 2004 and 2023. Twenty-two percent were conducted in North America (10 from the United States, 1 from the United States and Canada). Over 88% (n = 44) were conducted in HICs, with only 3 from lower-middle-income and 3 from upper-middle-income countries. Forty-one (82%) studies reported annual data, and 7 (14%) reported seasonal data. Of the seasonal studies, 2 lacked clear data collection periods [24, 53]. The remaining 5 seasonal studies spanned from 3 to 9 months [25, 26, 32, 46, 63]. One study combined annual and seasonal data, and the data type was unclear in 1 study. These 2 studies were excluded from the meta-analysis [48, 56].

**Table 1.** Characteristics of Included Studies

Study	Site (Country)	Study Start	Study End	Nature of Data	Case Definition	Chronic Condition Studied	Specimen Tested	Age Criteria for Inclusion in the Study	Age Summary Measure	Age Summary (y)	Sample Size (Number of Unique Individuals [Number of Tests])^a^	Sex Summary, Female (%):Male (%)
Akhmedov et al (2020) [20]	Ulm University Hospital (Germany)	2005	2018	Annual	URTI or LRTI	Allogeneic hematopoietic stem transplant recipients	Nasopharyngeal swab (URTI), bronchoalveolar lavage (LRTI)	>18 y	Median (range)	URTI: 53 (range 22–68); LRTI 55.5 (range 20–70); Total age range: 20–70	71 (71)	25 (35%):46 (65%)
Biancardi et al (2016) [21]	Prince of Wales Hospital, Sydney (Australia)	Oct 2014	Sep 2015	Annual	AECOPD	COPD	Nasopharyngeal aspirates	>18 y	Not reported	Unclear	153 (102)	76 (50%):77 (50%)
Brañas et al (2015) [22]	University Hospital 12 de Octubre, Madrid (Spain)	2004	2006	Annual	RTI	Immunocompromised	Nasopharyngeal swabs	Unclear	Median (interquartile range)	52 (18–84)	370 (595)	252 (42%):343 (58%)^b^
Campbell et al (2015) [23]	Fred Hutchinson Cancer Research Center (USA)	Dec 2005	Feb 2010	Annual	Weekly virologic surveillance (asymptomatic testing) or LRTI	Allogeneic hematopoietic cell transplant recipients	Nasal washes or nasopharyngeal swabs, oropharyngeal swabs	≥18 y	Not reported	Unclear	406 (406)	Unclear
Choi et al (2025) [24]	2 Atlanta-area hospitals (USA)	2018	2020	Seasonal	AECOPD or exacerbation of CHF	COPD or CHF	Combined nasopharyngeal and oropharyngeal swabs	>18 y	Median (interquartile range)	65 (58–73) for COPD; 61 (50–68) for CHD	311 (311) for COPD; 490 (490) for CHD	181 (58%):130 (42%) for COPD; 260 (53%):230 (47%) for CHD
Clark et al (2014) [25]	2 hospital sites with acute medical admission units, within the University Hospitals of Leicester NHS Trust (Leicester, UK)	Sep 2005	May 2008	Seasonal	Acute exacerbation of a chronic cardiorespiratory disease or new onset of acute cardiorespiratory illness	COPD	Nasopharyngeal swabs	>18 y	Median (interquartile range)	70 (62–77)	264 (264)	124 (47%):140 (53%)
De Serres et al (2009) [26]	2 university-affiliated hospitals, Province of Quebec (Canada)	Jan 2003	May 2004	Seasonal	AECOPD	COPD	Nasopharyngeal aspirates; acute and convalescent phase sera	≥50 y	Categories	50–59 y: 15; 60–69 y: 31; ≥70 y: 62	108 (108)	49 (45%):59 (55%)
Dimopoulos et al (2012) [27]	“SOTIRIA” Hospital, Athens (Greece)	Jan 2008	Dec 2009	Annual	AECOPD	COPD	Sputum and oropharyngeal swabs	>18 y	Mean (standard deviation)	69.7 (9.1)	200 (400)	50 (25%):150 (75%)
Djamin et al (2015) [28]	Amphia Hospital, Breda (the Netherlands)	May 2010	Jun 2013	Annual	AECOPD	COPD	Pharyngeal swabs	≥18 y	Not reported	Unclear	63 (136)	Unclear
Dumas et al (2023) [29]	72 ICUs in the country (France)	2003	2017	Annual	Acute respiratory failure	Immunocompromised	Blood, serum, nasopharyngeal aspirates	≥18 y	Not reported	Unclear	3067 (3067)	Unclear
Etherington et al (2014) [30]	Leeds Regional Adult CF Unit (UK)	Dec 2008	Nov 2009	Annual	Pulmonary exacerbation	Cystic fibrosis	Throat swabs	Unclear	Median (interquartile range)	Unclear	180 (432)	Unclear
Feikin et al (2012) [31]	Lwak Hospital (Kenya)	Mar 2007	Feb 2010	Annual	ARI	HIV positive	Nasopharyngeal and oropharyngeal swabs	≥18 y	Not reported	Unclear	Unclear	Unclear
Flight et al (2014) [32]	Manchester Adult Cystic Fibrosis Centre (UK)	Dec 2010	Mar 2011	Seasonal	Routine surveillance (asymptomatic testing) or pulmonary exacerbation, or upper respiratory tract infection	Cystic fibrosis	Sputum, nose swabs, and throat swabs	≥18 y	Median (interquartile range)	28 (23–36)	100 (626)	52 (52%):48 (48%)
Gao et al (2015) [33]	Outpatient clinics of The First Affiliated Hospital of Guangzhou Medical University (China) and a previous cohort study assessing the effects of anxiety and depression on bronchiectasis exacerbations	Feb 2013	Mar 2014	Annual	Bronchiectasis exacerbation	Bronchiectasis	Nasopharyngeal swabs and sputum	≥18 y	Mean (standard deviation)	44.27 (14.35)	58 (100)	38 (66%):20 (34%)
Gorse et al (2015) [34]	St Louis, Missouri (USA)	Nov 2009	Jul 2013	Annual	ARI	Chronic heart or lung disease or both	Nasopharyngeal and oropharyngeal swabs	≥60 y	Mean (standard deviation)	66.6 (20.3)	100 (100)	10 (10%):90 (90%)
Gottlieb et al (2009) [35]	Lung Transplant Outpatient Clinic, Hannover Medical School (Germany)	Oct 2005	Apr 2006	Annual	URTI or LRTI	Lung transplant recipients	Nasopharyngeal and oropharyngeal swabs, bronchoalveolar lavage	≥18 y	Median (range)	47 (18–68)	388 (447)	192 (50%):196 (50%)
Hong et al (2014) [18]	Asan Medical Center, Seoul (South Korea)	Mar 2010	Feb 2012	Annual	Severe hospital-acquired pneumonia	Immunocompromised	Nasopharyngeal specimens	Unclear	Mean (standard deviation)	64.5 (13.5)	119 (119)	Unclear
Hopkins et al (2008) [36]	Prince Charles Hospital (Australia)	Jul 2003	Dec 2006	Annual	ILI	Lung transplant recipients	Nasopharyngeal aspirate	Unclear	Not reported	Unclear	89 (199)	Unclear
Hosseini et al (2015) [37]	Divisions of Respiratory Diseases in Tehran Hospitals (Iran)	2010	2012	Annual	AECOPD	COPD	Sputum, nasal lavage, and throat wash samples	Unclear	Mean (standard deviation)	66 (8.9)	170 (170)	77 (45%):93 (55%)
Jang et al (2021) [38]	Yeungnam University Medical Center (South Korea)	Jan 2017	Dec 2018	Annual	AECOPD	COPD	Nasopharyngeal swabs	>40 y	Mean (standard deviation)	76.5 (7.9)	192 (262)	37 (19%):155 (81%)
Jethani et al (2025) [39]	Department of Medical Oncology, Dr Bheemrao Ambedkar Institute Rotary Cancer Hospital, All India Institute of Medical Sciences, New Delhi (India)	Jan 2017	Feb 2020	Annual	Respiratory viral infections	Hematopoietic stem-cell transplant	Nasal and throat swabs	>18 y	Median and mean	43.5 and 42	100 (158)	34 (34%):66 (66%)
Kim et al (2016) [40]	Asan Medical Center, Seoul (South Korea)	Jan 2010	Dec 2012	Annual	AECOPD or pneumonia	COPD	Nasal swabs or sputum samples, bronchoalveolar lavage or both	Unclear	Mean (standard deviation)	71.9 (9.3)	477 (477)	65 (14%):412 (86%)
Kim et al (2022) [41]	Fred Hutchinson Cancer Center (USA)	Mar 2010	Mar 2016	Annual	Symptomatic RVI	Hematological diseases^c^	Nasopharyngeal or nasal swabs for URI and bronchoalveolar lavage for LRD	≥18 y	Categories	18–75 y: 697; and >75 y: 7	704 (704)	302 (43%):402 (57%)
Ko et al (2007) [42]	Prince of Wales Hospital (Hong Kong)	May 2004	Apr 2005	Annual	AECOPD	COPD	Nasopharyngeal aspirates	Unclear	Mean (standard deviation)	75.7 (7.7)	196 (245)	36 (18%):160 (82%)
Koul et al (2017) [43]	Tertiary care facility in Srinagar (India)	May 2011	Sep 2012	Annual	AECOPD	COPD	Nasopharyngeal or oropharyngeal swabs	Unclear	Median (range)	65 (40–100)	233 (233)	81 (35%):152 (65%)
Kwak et al (2016) [44]	Hanyang University Hospital, Seoul (South Korea)	Jan 2010	Dec 2011	Annual	AECOPD	COPD	Nasopharyngeal swabs	Unclear	Mean (standard deviation)	69.2 (11.0)	213 (278)	73 (34%):140 (66%)
Lee et al (2021) [45]	28 hospitals in the country (South Korea)	Jan 2015	Dec 2018	Annual	Moderate-to-severe AECOPD	COPD	Sputum, bronchial washing fluid, endotracheal aspirate, or nasopharyngeal swabs	>40 y	Mean (standard deviation)	73.78 (9.2)	1186 (1186)	207 (17%):979 (83%)
Lokhandwala et al (2026) [66]	2 academic hospitals with National Cancer Institute–designated Comprehensive Cancer Centers: Oregon Health and Science University and Barnes Jewish Hospital (USA)	Jan 2019	Jun 2023	Annual	Physiologically significant RTIs	Hematologic malignancies	Unclear	Unclear	Median (interquartile range)	hMPV: 61 (38–72); RSV: 65 (53–72)	hMPV: 20; RSV: 28	HMPV: 5 (25%):15 (75%); RSV: 12 (43%):16 (57%)
Loubet et al (2021) [46]	5 teaching hospitals (for 4 seasons) and 6 teaching hospitals (for 2 seasons) in the country (France)	2012	2018	Seasonal	ILI	Chronic respiratory disease, chronic heart disease, diabetes, chronic renal failure, cancer, cirrhosis, immunosuppressant	Nasopharyngeal swabs	Unclear	Not reported	Unclear	3138 (3138)	Unclear
Maillard et al (2023) [47]	32 ICUs (France)	May 2016	Dec 2017	Annual	Acute respiratory failure	Immunocompromised	Nasopharyngeal swabs	≥18 y	Median (interquartile range)	64.0 (56.7–71.3)	510 (510)	167 (33%):343 (67%)
McManus et al (2008) [48]	Belfast (UK)	Unclear	Unclear	Unclear	AECOPD	COPD	Sputum	Unclear	Mean (standard deviation)	70.2 (9.4)	136 (136)	64 (47%):72 (53%)
Menéndez et al (2017) [49]	Specialized clinic of 2 tertiary care hospitals (Spain)	2011	2015	Annual	Bronchiectasis exacerbation	Bronchiectasis	Sputum, blood sample, and/or nasopharyngeal specimens	Unclear	Not reported	Unclear	233 (233)	Unclear
Murphy et al (2013) [19]	John Hunter Hospital, Newcastle (Australia)	Apr 2007	Nov 2009	Annual	Common cold	Asthma in pregnant women	Nasal and throat swabs	>18 y	Not reported	Unclear	168 (168)	Not applicable
Park et al (2013) [50]	Asan Medical Center, Seoul (South Korea)	Jan 2009	Feb 2012	Annual	RVI (URI or LRI)	Hematologic diseases	Nasopharyngeal aspirates and swab specimens	Unclear	Not reported	Unclear	737 (737)	64 (44%):81 (56%)^d^
Peghin et al (2017) [51]	Hospital Univeristari Vall d’Hebron, Barcelona (Spain)	Sep 2009	Sep 2011	Annual	Respiratory tract infectious disease (URTID or LRTID)	Lung transplant recipients	Nasopharyngeal swabs	>18 y	Mean (standard deviation)	49.9 (12.6)	98 (292)	36 (37%):62 (63%)
Piñana et al (2020) [52]	2 transplant centers in Valencia (Spain)	Dec 2013	Dec 2018	Annual	URTD or LRTD	Allogeneic hematopoietic cell transplant recipients	Upper and/or lower respiratory specimens	>18 y	Median (range)	47 (18–70)	233 (376)	103 (44%):130 (56%)
Ponsford et al (2021) [17]	Immunodeficiency Centre for Wales, Cardiff (UK)	Aug 2015	Jan 2018	Annual	Fortnightly routine surveillance (asymptomatic testing) or RTI (URTI or LRTI)	Primary antibody deficiency	Nasal swabs	>18 y	Median (range)	51.5 (21–78)	44 (870)	21 (48%):23 (52%)
Rachow et al (2020) [53]	Jena (Germany)	2014	2017	Seasonal	At least twice at a 3-wk interval independent of symptoms (asymptomatic testing)	Allogeneic stem-cell transplant recipients	Throat gargles	≥18 y	Median (range)	56 (21–72)	194 (426)	79 (41%):115 (59%)
Reckziegel et al (2020) [54]	Jena University Hospital (Germany)	Sep 2009	Dec 2012	Annual	RTI	Immunocompromised	Upper (nasal or throat swabs and washings) and lower (bronchoalveolar/tracheal washings, induced sputum) respiratory tract	Unclear	Median	57.2	188 (188)	87 (46%):101 (54%)
Samannodi et al (2021) [55]	8 hospitals in the Memorial Hermann Health System in Houston (USA)	Mar 2016	Apr 2019	Annual	Patient hospitalized for any reason	Solid organ transplant recipients	Nasopharyngeal specimens	Unclear	Median	62	102 (102)	58 (57%):44 (43%)
Samoriski et al (2025) [56]	Rochester General Hospital, University of Rochester Medical Center (USA)	2008	2023	Both: Nov to May from 2008 to 2011 (seasonal) and Mar 2019 to Apr 2023 (annual)	Acute cardiopulmonary illness or ARI	COPD, asthma, coronary artery disease, congestive heart failure, diabetes mellitus, chronic kidney disease, substance use disorder and dementia	Nasal swabs	≥18 y	Mean (standard deviation)	62.2 (16.6)	Unclear (1072)	585 (55%):487 (45%)
Seo et al (2017) [58]	A tertiary hospital (South Korea)	Jun 2009	Jun 2014	Annual	Exacerbate LRTI	Asthma	Sputum	Unclear	Mean (standard deviation)	56.30 (0.90)	Unclear (259)	176 (68%):84 (32%)
Seo et al (2022) [57]	28 hospitals (South Korea)	Jan 2015	Dec 2018	Annual	AECOPD	COPD	Throat or nasopharyngeal swabs	>40 y	Mean (standard deviation)	73.78 (9.22)	1186 (1186)	207 (17%):979 (83%)
Spahr et al (2018) [59]	University Hospital Basel (Switzerland)	Jun 2010	Dec 2014	Annual	RTID (URTID or LRTID)	Allogeneic hematopoietic cell transplant	Nasopharyngeal swabs and bronchoalveolar lavage	>18 y	Median (range)	52.3 (19.9–70.6)^4^	66 (103)	31 (30%):72 (70%)
Thornton et al (2024) [60]	11 adult and pediatric CFF Therapeutic Development Network sites (USA and Canada)	Jul 2016	Jan 2020	Annual	Pulmonary exacerbation	Cystic fibrosis	Sputum	≥18 y	Not reported	Unclear	621 (1254)	285 (46%):336 (54%)
Wee et al (2025) [65]	Singapore General Hospital (Singapore)	Jan 2018	May 2024	Annual	Acute respiratory symptoms	Cancer	Upper or lower respiratory tract specimen	≥21 y	Median (interquartile range)	hMPV: 63 (55–73); RSV: 66 (57–72)	hMPV: 128; RSV: 235	hMPV: 58 (45%):70 (55%); RSV: 105 (45%):130 (55%)
Weinberg et al (2010) [61]	University of Colorado Denver (USA)	Sep 2005	Nov 2007	Annual	RTI (URTI or LRTI)	Lung transplant recipients	Nasal washes or bronchoalveolar lavage	>18 y	Median (range)	60 (26–73)	60 (112)	23 (38%):37 (62%)
Widmer et al (2012) [62]	Davidson, Tennessee (during the 2006–2007 season, 1 academic and 1 community hospital; during the 2007–2008 season, 1 academic hospital; and during the 2008–2009 season, 1 academic and 3 community hospitals) (USA)	Nov 2006	Apr 2009	Seasonal	RTI	Cardiovascular disease, pulmonary disease, diabetes mellitus, immunodeficiency	Nasal and throat swab specimens	≥50 y	Median (interquartile range)	66 (58–78)	508 (508)	305 (60%):203 (40%)
Widmer et al (2012) [62]	1 academic emergency department, 1 academic hospital, and 3 community hospitals (USA)	May 2009	Apr 2010	Annual	Respiratory symptoms or nonlocalizing fever within 7 d before presentation	Cardiovascular disease, pulmonary disease, diabetes mellitus, and immunodeficiency	Nasal and throat swabs	≥18 y	Median (interquartile range)	51.1 (35.3–65.5)	1248 (1248)	735 (59%):513 (41%)
Yin et al (2018) [64]	Jiangchuan Community, Minhang District in Shanghai (China)	Jun 2013	May 2015	Annual	AECOPD	COPD	Oropharyngeal swabs	>18 y	Mean (standard deviation)	75 (0.5)	264 (264)	67 (25%):197 (75%)

Abbreviations: AECOPD, acute exacerbation of chronic obstructive pulmonary disease; ARI, acute respiratory infection; CHD, chronic heart disease; CHF, chronic heart failure; COPD, chronic obstructive pulmonary disease; HIV, human immunodeficiency virus; ILI, influenza-like illness; LRI, lower respiratory infection; LRTD, lower respiratory tract disease; LRTI, lower respiratory tract infection; RTI, respiratory tract infection; RTID, respiratory tract infectious disease; RVI, respiratory viral infection; URI, upper respiratory infection; URTD, upper respiratory tract disease; URTI, upper respiratory tract infection.

^a^Eligibility criteria were applied at the outcome level. The ≥100 denominator threshold was required for inclusion in test positivity analyses; however, studies were also eligible if they reported at least one other predefined outcome of interest (eg hospitalization, ICU admission, or mortality), as specified in Supplementary Text 1. Sample sizes may therefore vary across outcomes reported within individual studies.

^b^Sex summary for number of tests rather than number of unique individuals.

^c^The study examined respiratory viral infections in adults within 90 days before autologous and allogeneic HCT at the Fred Hutchinson Cancer Center. The study did not report the precise underlying indications for transplantation. Given that autologous and allogeneic hematopoietic cell transplantation in adults are predominantly performed for hematological diseases, the study population was assumed to have an underlying chronic hematological condition and was classified accordingly for this analysis.

^d^Summary of patients with a positive test for at least 1 virus (human metapneumovirus, respiratory syncytial virus, or parainfluenza virus) and does not include those who tested negative for the 3 viruses.

Chronic obstructive pulmonary disease (COPD) was the most commonly studied underlying condition, reported in 17 (34%) studies. Two studies used nucleic acid testing for viral confirmation (hMPV and RSV) [51, 59]. All remaining studies used polymerase chain reaction (PCR). Only 1 study employed paired serology with PCR [26]. Supplementary Table 5 presents the complete case definitions for the chronic underlying health conditions, and either RTI case definitions or the testing criteria used in each study.

Forty-two studies contributed 63 data points for hMPV and RSV test positivity, that is the proportion of episodes testing positive for each virus in their study populations. The distribution of chronic underlying health conditions and data collection periods is provided in Supplementary Table 4.

Our meta-analysis using annual data, based on 29 studies (32 data points, in adults with at least 1 chronic underlying health condition, showed that the pooled hMPV test positivity was 2.11% (95% CI: 1.57–2.83; *I*^2^ = 73.8%) in inpatient settings, 1.93% (95% CI: 0.94–3.92; *I*^2^ = 70.9%) in outpatient settings, and 1.51% (95% CI: 0.50–4.45; *I*^2^ = 93.5%) in studies reporting from mixed (including both inpatients and outpatients) settings. The RSV test positivity in the same populations was 3.87% (95% CI: 2.59–5.73; *I*^2^ = 95.7%) in inpatient settings, 2.46% (95% CI: 1.29–4.61; *I*^2^ = 68.8%) in outpatient settings, and 2.52% (95% CI: 1.21–5.16, *I*^2^ = 90.9%) in studies reporting from mixed settings. These conventional random-effects meta-analysis estimates are presented with the descriptive summaries of study-level proportions in Supplementary Figure 1.

Among adults with at least 1 chronic underlying health condition, the odds of testing positive for RSV were 61% to 119% higher than for hMPV in inpatient settings. In studies conducted in outpatient or mixed (including inpatients and outpatients) settings, the odds of testing positive for hMPV did not differ significantly from RSV (Table 2). Supplementary Figure 2 illustrates the GLMM-predicted proportions of hMPV and RSV test positivity by settings.

**Table 2.** Odds of RSV Relative to hMPV Test Positivity in Different Study Settings in Adults With at Least 1 Chronic Underlying Health Condition, Using Annual Data

Setting	OR (95% CI)	*Z* value	*P* value
Inpatient (n = 22)	1.88 (1.61–2.19)	8.07	<.001
Outpatient (n = 5)	1.07 (0.64–1.80)	0.27	.786
Mixed (n = 5)	1.44 (0.95–2.20)	1.72	.086
Overall (n = 32)	1.65 (1.43–1.91)	6.82	<.001

Abbreviations: CI, confidence interval; n, number of data points; OR, odds ratio.

Supplementary Figure 3 demonstrates the GLMM-predicted population-level ORs of RSV versus hMPV test positivity from the leave-one-out analysis. Exclusion of the study by Kim et al, which did not report underlying disease before HCT, did not significantly affect the results [41]. After excluding 8 influential studies, the odds of RSV versus hMPV test positivity among adults with at least 1 chronic condition remained higher in inpatient settings (OR = 1.84, 95% CI: 1.52–2.22), but not in outpatient (OR = 1.00, 95% CI: 0.44–2.29) or mixed settings (OR = 1.66, 95% CI: 0.91–3.03).

Fifteen studies (18 data points) included in this meta-analysis were rated as good quality (Supplementary Table 7). In sensitivity analyses restricted to these studies, the inpatient OR remained statistically significant, the outpatient estimate remained nonsignificant, and the mixed-setting OR became statistically significant (Supplementary Table 8).

Restriction to HICs showed a similar setting-specific pattern, with higher odds of RSV test positivity in inpatient settings only (Supplementary Table 9). Supplementary Figure 4 illustrates the GLMM-predicted proportions of hMPV and RSV test positivity in HICs by settings. Estimates for both viruses were generally higher than the corresponding overall (global) estimates in inpatient and mixed settings, but lower than those in outpatient settings, although with overlapping 95% CI across all settings. Seasonal data analyses (7 studies, 17 data points) are presented in Supplementary Figure 5 and Supplementary Table 10. The overall effect estimate (OR 1.68, 95% CI 1.42–2.00) was consistent with a similar positive association observed using annual data (OR 1.65, 95% CI 1.43–1.91). Subgroup estimates were broadly comparable between analyses, with inpatient settings showing stable and statistically significant differences in RSV versus hMPV odds in both approaches, while outpatient and mixed settings remained imprecise and not statistically significant. GLMM-predicted proportions were generally consistent between seasonal and annual models, with overlapping 95% confidence intervals across estimates. The remaining 5 studies (11 data points) were excluded from these meta-analyses because of the unclear nature of the data collection period (n = 1), data points combining estimates from both seasonal and annual data (n = 7), and data points from studies with unclear study settings (n = 3; Supplementary Table 11) [48, 54, 56, 58, 61].

Among studies excluded from the meta-analysis for distinct populations, 1 study of pregnant women with asthma identified 15 hMPV-positive cases (8.9%, 95% CI: 4.6–13.2) and 2 RSV-positive cases (1.2%, 95% CI: 0–2.8) among 168 common cold episodes [19]. Another study of 119 immunocompromised adults with severe HAP reported 1 hMPV-positive episode (0.8%, 95% CI: 0–2.5) and 13 RSV-positive episodes (10.9%, 95% CI: 5.3–16.5) [18]. Ponsford et al investigated adults with primary antibody deficiency using self-swabbing and postal sample submission, indicating a community-based setting. Of 870 tests, 16 were RSV positive (1.84%, 95% CI: 0.95–2.73) and 7 were hMPV-positive (0.80%, 95% CI: 0.21–1.40) [17].

At least 3 studies provided annual or seasonal data for chronic respiratory disease, solid organ transplant recipients, hematologic diseases, immunocompromised individuals, and cardiovascular disease. Random-effects pooled estimates of hMPV and RSV test positivity are presented in Supplementary Figure 6a–f, and the GLMM results in Table 3. Statistically significant differences in the RSV versus hMPV test positivity odds were observed for chronic respiratory disease (annual and seasonal), cardiovascular disease (seasonal), immunocompromised status (annual), and hematological disease (annual) but not among solid organ transplant recipients (annual).

**Table 3.** hMPV and RSV Predicted Test Positivity and Odds of RSV Relative to hMPV Test Positivity in Adults With 6 Underlying Condition Groups

Comorbidity Group	Data Collection Duration	Number of Studies	hMPV Predicted Positive % (95% CI)	RSV Predicted Positive % (95% CI)	Odds Ratio (95% CI)	*P* value
Chronic respiratory disease	Annual	18	1.89 (1.19–3.01)	3.76 (2.41–5.83)	2.02 (1.67–2.45)	<.001
Chronic respiratory disease	Seasonal	6	3.17 (2.40–4.17)	4.44 (3.44–5.71)	1.42 (1.09–1.85)	.009
Cardiovascular disease	Seasonal	3	3.04 (2.01–4.55)	4.56 (3.11–6.64)	1.53 (1.11–2.10)	.009
Solid organ transplant	Annual	5	3.95 (2.15–7.18)	4.13 (2.25–7.46)	1.05 (0.69–1.57)	.816
Immunocompromised	Annual	6	1.48 (0.98–2.25)	2.17 (1.46–3.21)	1.47 (1.10–1.97)	.010
Hematological diseases	Annual	4	2.15 (1.07–4.25)	4.43 (2.31–8.33)	2.11 (1.49–3.00)	<.001

Abbreviations: CI, confidence interval; hMPV, human metapneumovirus; OR, odds ratio; RSV, respiratory syncytial virus.

Findings from the 2 studies reporting hMPV and RSV test positivity in adults with COPD, with other comorbidities, are presented in Supplementary Table 12 [45, 57].

Only 3 studies estimated the incidence of hMPV and RSV in adults with chronic underlying health conditions (Table 4) [31, 32, 39]. The incidence of hMPV appeared higher than RSV among adults with human immunodeficiency virus (HIV) and cystic fibrosis [31, 32]. However, the difference was statistically significant only in cystic fibrosis patients [32]. The third study did not report 95% CI, preventing statistical comparisons. In this study on hematopoietic stem-cell transplant patients, RSV incidence appeared comparable to hMPV incidence [39].

**Table 4.** hMPV and RSV Incidence Reported in Adults With Chronic Underlying Health Conditions in Included Studies

Study	Chronic Underlying Health Condition Studied	Data Collection Period	Case Definition for Testing	Setting	hMPV Incidence (95% CI)	RSV Incidence (95% CI)	*Z* value	*P* value
Feikin 2012	HIV positive	annual	ARI	outpatient	1.09 (0.41–1.77) per 100 PYO	0.98 (0.51–1.45) per 100 PYO	0.26	.794
Flight 2014	Cystic fibrosis	seasonal	Follow-up visit every 2 m, and at the time of pulmonary exacerbation	outpatient	0.28 (0.17–0.39) cases per patient-year	0.04 (0.00–0.09) cases per patient-year	3.96	<.001
Jethani 2025	HSCT	annual	Symptoms of respiratory viral infections	Inpatient	8.9 per 100 patients per year	10 per 100 patients per year	NA	NA

Abbreviations: ARI, acute respiratory infections; CI, confidence interval; HIV, human immunodeficiency virus; hMPV, human metapneumovirus; HSCT, hematopoietic stem-cell transplantation; PYO, patient-years of observation; RSV, respiratory syncytial virus; NA, *Z* value and *P* value could not be derived as the 95% CI were not estimated in the study and could not be derived.

Findings from studies reporting the proportion of hMPV and RSV episodes requiring hospital admission, ICU admission, and case fatality rate (CFR) are presented in Supplementary Tables 13, 14, and 15, respectively.

## DISCUSSION

To our knowledge, this is the first study to systematically summarize the literature and to directly compare the global burden of hMPV and RSV in adults with chronic underlying health conditions. Our findings indicated 43%–91% higher odds of RSV versus hMPV test positivity across study settings in this population. This difference reached statistical significance in inpatient settings (69% of included studies) but not in outpatient or mixed settings. Overall pooled setting-specific estimates may be influenced by the stronger contribution of inpatient studies, which likely reflects the distribution of available evidence and may affect comparability across settings. Considerable heterogeneity among studies likely contributed to uncertainty in population-average estimates. Limited and heterogeneous data on incidence, disease severity, and CFR prevented meta-analysis and definitive conclusions.

Our conventional meta-analysis showed higher pooled RSV test positivity than hMPV across all 3 settings, although heterogeneity was moderate to high (*I*^2^ range: 69%–96%; Supplementary Figure 1). This heterogeneity was expected given variations in study populations, enrollment criteria, RTI case definitions, testing policies, sample sizes, chronic conditions, and health-seeking behavior across studies (Table 1). It persisted in subgroup analyses by chronic condition and data collection period (Supplementary Figure 6a–f). Included studies also varied substantially in sample size, which may have influenced the precision of individual estimates and resulted in greater contribution of larger studies to pooled estimates. Importantly, RSV and hMPV testing was performed in the same individuals within each study, enabling direct within-study comparisons and controlling for individual-level confounding. To further account for between-study variability, we applied a GLMM with a random study intercept (Supplementary Figure 2). This approach appropriately modeled the binomial outcome, preserved the paired data structure, reduced the influence of individual studies, and enabled estimation of population-average ORs for RSV relative to hMPV (Table 2).

Our test positivity estimates of 2.28% (95% CI: 1.43–3.60) for hMPV and 4.15% (95% CI: 2.65–6.45) for RSV among adults with chronic underlying health conditions in inpatient settings in HICs (Supplementary Figure 4) are comparable to the published proportion positive in ARI hospitalization estimates for hMPV (2.46% [95% CI: 1.33–4.51]) and RSV (4.4% [95% CI: 3.0–6.5]), respectively, in ≥65 years in HICs [67, 68]. This suggests that adults with chronic underlying health conditions in HICs likely experience an hMPV and RSV burden comparable to older adults, supporting their inclusion as a priority group in future RSV (and, when available) hMPV vaccine policies. However, this interpretation should be viewed with caution because it was not possible to separately assess the overlap between older age and chronic disease populations, owing to inconsistent and nonstandardized age reporting across studies.

The 8 studies excluded in the leave-one-out analysis were of good quality, indicating that the outlying estimates were unlikely to reflect poor study quality (Supplementary Figure 3) [20, 23, 27, 33, 36, 45, 51, 57, 62]. Instead, these differences likely reflect variation in populations, testing strategies, methods, and contextual factors. For example, Dimopoulos et al reported unusually high RSV test positivity (40.5%) in a COPD cohort (Supplementary Figure 1), possibly due to chronic viral persistence rather than acute infection, as quantitative PCR was unavailable [27]. Excluding influential studies did not significantly alter the ORs, indicating the general robustness of the main analysis. Even in the sensitivity analysis restricted to good-quality studies, RSV versus hMPV test positivity remained statistically significant in inpatient settings and became significant in mixed settings. However, estimates in mixed (n = 2) and outpatient (n = 3) settings remain uncertain due to the small number of studies (Supplementary Table 8). Studies excluded from the meta-analysis also reported higher RSV than hMPV test positivity, consistent with overall findings (Supplementary Table 11).

Despite restricting inclusion to studies assessing the same respiratory episodes for hMPV and RSV, several factors may have influenced comparative detection of hMPV and RSV. Respiratory syncytial virus diagnostic sensitivity varies by testing approach, specimen type, case definition, and timing of specimen collection [69–71]. Most studies (n = 41) used PCR, yet performance differences between PCR assays have been documented [72]. Comparable variability likely exists for hMPV, although supporting evidence is less definitive. Consequently, reliance on a single specimen type and testing method in most studies may have underestimated the true burden of both viruses. Seasonal analyses should also be interpreted cautiously, as hMPV and RSV circulation periods often do not fully overlap (Supplementary Figures 5 and 6b, c, Supplementary Table 10) [73, 74]. Moreover, seasonal definitions were often derived from influenza surveillance, which is suboptimal for both viruses. These factors may have differentially affected RSV and hMPV detection, complicating the interpretation of bias in this comparative analysis.

Incidence data were heterogeneous and sparse, with inconsistent patterns of hMPV and RSV reported across the 3 studies (Table 4). Two studies reported higher hMPV incidence than RSV, reaching statistical significance in only one [31, 32]. The third study found higher RSV incidence, but the significance could not be assessed [39]. While test positivity provides some insight, available incidence data are insufficient to draw firm conclusions regarding the relative incidence of RSV versus hMPV. Reported hospitalization, ICU admission, and CFR patterns were similarly inconsistent, with no statistically significant differences in individual studies (Supplementary Tables 13–15). Substantial heterogeneity in chronic conditions, case definitions, and healthcare settings further limited comparability, precluding pooled estimates or overarching conclusions.

Only one study reported HAP, limited to severe cases, detecting hMPV in 1 episode and RSV in 13 episodes (test positivity 0.8% vs 10.9%) [18]. This scarcity of data on HAP is an important evidence gap, as adults with chronic conditions may face higher HAP risk due to frequent healthcare exposure and impaired immunity. Most studies (88%) were conducted in HICs, preventing estimates for low- and middle-income regions, where infectious disease burden and multimorbidity are substantial, limiting generalizability [75, 76].

Whether multimorbidity influences hMPV or RSV risk remains unclear, with evidence limited to 2 COPD studies (Supplementary Table 12) [45, 57]. Respiratory syncytial virus positivity exceeded hMPV in COPD with interstitial lung disease, chronic kidney disease, congestive heart failure, bronchiectasis, previous pulmonary tuberculosis, or asthma, but was lower in COPD with diabetes. Small subgroup sizes (<100) for COPD with interstitial lung disease or chronic kidney disease limit reliability. Further studies are needed to determine how infection risk varies by the number and type of coexisting conditions.

In addition to the limitations of the included studies, our approaches had some constraints. First, studies reporting exclusively on hMPV or RSV were excluded to allow direct within-study comparisons and reduce potential study-related biases and confounders. Second, most included studies were descriptive and not designed to estimate effect sizes, precluding formal assessment of publication bias. Heterogeneity in populations and study designs further complicated such assessments, though selective reporting cannot be ruled out.

## CONCLUSIONS

Human metapneumovirus and RSV contribute substantially to the respiratory disease burden among adults with chronic underlying health conditions, with RSV generally being more frequent. Clinical severity and mortality in these populations may be similar between the viruses but well-designed, adequately powered studies are needed to confirm this. Important evidence gaps include limited data from low- and middle-income countries and sparse characterization of hospital-acquired infections, underscoring the need for more comprehensive and geographically diverse research.

## Supplementary Material

ofag343_Supplementary_Data

## Supplementary Data

Supplementary materials are available at *Open Forum Infectious Diseases* online. Consisting of data provided by the authors to benefit the reader, the posted materials are not copyedited and are the sole responsibility of the authors, so questions or comments should be addressed to the corresponding author.
