# Supplementary material for: Comparison of Human Metapneumovirus- and Respiratory Syncytial Virus–Associated Health Burden in Adults With Chronic Underlying Health Conditions: A Global Systematic Review and Meta-analysis
Source: Open Forum Infect Dis. 2026 Jun 8;13(6):ofag343. doi: 10.1093/ofid/ofag343 (PMC13251343; doi:10.1093/ofid/ofag343)
Supplement: ofag343_Supplementary_Data [file ofag343_supplementary_data.docx]

Table of Contents

[Supplementary Table S1: PRISMA 2020 checklists 3](#_Toc222686839)

[PRISMA 2020 checklist 3](#_Toc222686840)

[PRISMA 2020 Abstract checklist 5](#_Toc222686841)

[Supplementary Table S2: Search strategies in databases 7](#_Toc222686842)

[Supplementary Text S1: Eligibility criteria for inclusion of studies in the systematic review 14](#_Toc222686843)

[Supplementary Table S3: Classification of chronic underlying health conditions reported in studies into broad groups 16](#_Toc222686844)

[Supplementary Table S4: Mapping the distribution of chronic underlying health conditions and data collection periods in studies reporting test positivity 18](#_Toc222686845)

[Supplementary Table S5: Case definitions used by individual study authors 19](#_Toc222686846)

[Supplementary Table S6: List of studies excluded at the full text screening stage, with the reason for exclusion 31](#_Toc222686847)

[Supplementary Figure S1: Forest plot of random effects meta-analysis estimating the hMPV and RSV positivity in individuals with at least one chronic underlying health condition, stratified by study setting, restricting to studies that collected data over at least one calendar year (annual) 44](#_Toc222686848)

[Supplementary Figure S2: GLMM-predicted proportions of hMPV and RSV positivity in different study settings (population-average estimates) with 95% CI, using annual data 47](#_Toc222686849)

[Supplementary Figure S3: Leave-one-out sensitivity analysis 48](#_Toc222686850)

[Supplementary Table S7: Quality appraisal of included studies 49](#_Toc222686851)

[Cohort studies 49](#_Toc222686852)

[Cross-sectional studies 53](#_Toc222686853)

[Supplementary Table S8: Sensitivity analysis including good quality studies only 54](#_Toc222686854)

[Supplementary Table S9: Odds of RSV relative to hMPV test positivity in different study settings in adults with at least one chronic underlying condition in high-income countries, using annual data 55](#_Toc222686855)

[Supplementary Figure S4: GLMM-predicted proportions of hMPV and RSV positivity in different study settings in high-income countries (Population-average estimates) with 95% CI, using annual data 56](#_Toc222686856)

[Supplementary Figure S5: GLMM-predicted proportions of hMPV and RSV positivity in different study settings (Population-average estimates) with 95% CI, using seasonal data 57](#_Toc222686857)

[Supplementary Table S10: Odds of RSV relative to hMPV test positivity in different study settings in adults with at least one chronic underlying health condition, using seasonal data 58](#_Toc222686858)

[Supplementary Table S11: hMPV and RSV test positivity reported in studies excluded from the meta-analysis 59](#_Toc222686859)

[Supplementary Figure S6: Meta-analysis by chronic underlying condition groups and period of data collection 60](#_Toc222686860)

[Supplementary Figure S6a: Chronic respiratory disease using annual data 60](#_Toc222686861)

[Supplementary Figure S6b: Chronic respiratory disease using seasonal data 61](#_Toc222686862)

[Supplementary Figure S6c: Cardiovascular disease using seasonal data 61](#_Toc222686863)

[Supplementary Figure S6d: Organ transplant using annual data 62](#_Toc222686864)

[Supplementary Figure S6e: Immunocompromised status using annual data 62](#_Toc222686865)

[Supplementary Figure S6f: Haematologic disease using annual data 63](#_Toc222686866)

[Supplementary Table S12: Findings from studies reporting hMPV and RSV test positivity in adults with COPD, along with other chronic conditions 64](#_Toc222686867)

[Supplementary Table S13: Proportion of episodes requiring hospital admission in individual studies 65](#_Toc222686868)

[Supplementary Table S14: Proportion of episodes requiring intensive care unit (ICU) admission in individual studies 66](#_Toc222686869)

[Supplementary Table S15: Case fatality rate (CFR) in individual studies 67](#_Toc222686870)

# Supplementary Table S1: PRISMA 2020 checklists

## PRISMA 2020 checklist

| **Section and Topic** | **Item #** | **Checklist item** | **Location where item is reported** |
| --- | --- | --- | --- |
| **TITLE** | | |  |
| Title | 1 | Identify the report as a systematic review. | Page 1 (lines 2-3) |
| **ABSTRACT** | | |  |
| Abstract | 2 | See the PRISMA 2020 for Abstracts checklist. |  |
| **INTRODUCTION** | | |  |
| Rationale | 3 | Describe the rationale for the review in the context of existing knowledge. | Page 3 (lines 2-14) |
| Objectives | 4 | Provide an explicit statement of the objective(s) or question(s) the review addresses. | Page 2 (lines 25-27) |
| **METHODS** | | |  |
| Eligibility criteria | 5 | Specify the inclusion and exclusion criteria for the review and how studies were grouped for the syntheses. | Page 4 (lines 11-23) |
| Information sources | 6 | Specify all databases, registers, websites, organisations, reference lists and other sources searched or consulted to identify studies. Specify the date when each source was last searched or consulted. | Page 4 (lines 4-6) |
| Search strategy | 7 | Present the full search strategies for all databases, registers and websites, including any filters and limits used. | Supplementary Table S2 |
| Selection process | 8 | Specify the methods used to decide whether a study met the inclusion criteria of the review, including how many reviewers screened each record and each report retrieved, whether they worked independently, and if applicable, details of automation tools used in the process. | Page 4 (lines 26-28) |
| Data collection process | 9 | Specify the methods used to collect data from reports, including how many reviewers collected data from each report, whether they worked independently, any processes for obtaining or confirming data from study investigators, and if applicable, details of automation tools used in the process. | Page 4 (lines 28-29) |
| Data items | 10a | List and define all outcomes for which data were sought. Specify whether all results that were compatible with each outcome domain in each study were sought (e.g. for all measures, time points, analyses), and if not, the methods used to decide which results to collect. | Page 4 (lines29-33, page 5 lines 1-4) |
|  | 10b | List and define all other variables for which data were sought (e.g. participant and intervention characteristics, funding sources). Describe any assumptions made about any missing or unclear information. | Not applicable |
| Study risk of bias assessment | 11 | Specify the methods used to assess risk of bias in the included studies, including details of the tool(s) used, how many reviewers assessed each study and whether they worked independently, and if applicable, details of automation tools used in the process. | Page 5 lines 6-9 |
| Effect measures | 12 | Specify for each outcome the effect measure(s) (e.g. risk ratio, mean difference) used in the synthesis or presentation of results. | Page 4 line 33, page 5 lies 1-4 |
| Synthesis methods | 13a | Describe the processes used to decide which studies were eligible for each synthesis (e.g. tabulating the study intervention characteristics and comparing against the planned groups for each synthesis (item #5)). | Page 7 line 30, page 8 lines 1-2 |
|  | 13b | Describe any methods required to prepare the data for presentation or synthesis, such as handling of missing summary statistics, or data conversions. | Not applicable |
|  | 13c | Describe any methods used to tabulate or visually display results of individual studies and syntheses. | Supplementary Table S4 |
|  | 13d | Describe any methods used to synthesize results and provide a rationale for the choice(s). If meta-analysis was performed, describe the model(s), method(s) to identify the presence and extent of statistical heterogeneity, and software package(s) used. | Page 5 lines11-29, page 6 lines 1-26 |
|  | 13e | Describe any methods used to explore possible causes of heterogeneity among study results (e.g. subgroup analysis, meta-regression). | Page 6 lines 11-14; page 6 lines 22-26 |
|  | 13f | Describe any sensitivity analyses conducted to assess robustness of the synthesized results. | Page 6 lines 5-10 |
| Reporting bias assessment | 14 | Describe any methods used to assess risk of bias due to missing results in a synthesis (arising from reporting biases). | Not applicable |
| Certainty assessment | 15 | Describe any methods used to assess certainty (or confidence) in the body of evidence for an outcome. | Page 5 lines 28-29, page 6 lines 1-3 |
| **RESULTS** | | |  |
| Study selection | 16a | Describe the results of the search and selection process, from the number of records identified in the search to the number of studies included in the review, ideally using a flow diagram. | Figure 1 |
|  | 16b | Cite studies that might appear to meet the inclusion criteria, but which were excluded, and explain why they were excluded. | Supplementary table S5 |
| Study characteristics | 17 | Cite each included study and present its characteristics. | Table 1 |
| Risk of bias in studies | 18 | Present assessments of risk of bias for each included study. | Supplementary table S8 |
| Results of individual studies | 19 | For all outcomes, present, for each study: (a) summary statistics for each group (where appropriate) and (b) an effect estimate and its precision (e.g. confidence/credible interval), ideally using structured tables or plots. | Supplementary Figure S1, Supplementary Tables S13, S14, s15 |
| Results of syntheses | 20a | For each synthesis, briefly summarise the characteristics and risk of bias among contributing studies. | Page 8 lines 25-26 |
|  | 20b | Present results of all statistical syntheses conducted. If meta-analysis was done, present for each the summary estimate and its precision (e.g. confidence/credible interval) and measures of statistical heterogeneity. If comparing groups, describe the direction of the effect. | Supplementary Figure S1, Supplementary Figures S5a-f |
|  | 20c | Present results of all investigations of possible causes of heterogeneity among study results. | Supplementary Figure S3, Supplementary Table S9, Supplementary Figures S5a-e |
|  | 20d | Present results of all sensitivity analyses conducted to assess the robustness of the synthesized results. | Page 8 lines 17-28 |
| Reporting biases | 21 | Present assessments of risk of bias due to missing results (arising from reporting biases) for each synthesis assessed. | Not applicable |
| Certainty of evidence | 22 | Present assessments of certainty (or confidence) in the body of evidence for each outcome assessed. | Table 2, Table 3, Supplementary Figure S1, Supplementary Figure |
| **DISCUSSION** | | |  |
| Discussion | 23a | Provide a general interpretation of the results in the context of other evidence. | Page 10 lines 14-22, page 12 lines 15-20 |
|  | 23b | Discuss any limitations of the evidence included in the review. | Page 10 lines 29-29, page 11 lines 32-33 and page 12 lines 1 to 11, page 12 lines 25-28 |
|  | 23c | Discuss any limitations of the review processes used. | Page 13 (lines 26-31) page 14 (lines 1-2) |
|  | 23d | Discuss implications of the results for practice, policy, and future research. | Page 14 lines 4-10 |
| **OTHER INFORMATION** | | |  |
| Registration and protocol | 24a | Provide registration information for the review, including register name and registration number, or state that the review was not registered. | Page 3 (line 31) Page 4 line 1 |
|  | 24b | Indicate where the review protocol can be accessed, or state that a protocol was not prepared. | Page 3 (line 31) Page 4 line 1 |
|  | 24c | Describe and explain any amendments to information provided at registration or in the protocol. | Not applicable |
| Support | 25 | Describe sources of financial or non-financial support for the review, and the role of the funders or sponsors in the review. | Page 7 lines 5-6) |
| Competing interests | 26 | Declare any competing interests of review authors. | Page 14 (lines 14-18) |
| Availability of data, code and other materials | 27 | Report which of the following are publicly available and where they can be found: template data collection forms; data extracted from included studies; data used for all analyses; analytic code; any other materials used in the review. | Page 14 (lines 11-12) |

## PRISMA 2020 Abstract checklist

| **Section and Topic** | **Item #** | **Checklist item** | **Reported (Yes/No)** |
| --- | --- | --- | --- |
| **TITLE** | | |  |
| Title | 1 | Identify the report as a systematic review. | Title (Page 1 lines 2-4) |
| **BACKGROUND** | | |  |
| Objectives | 2 | Provide an explicit statement of the main objective(s) or question(s) the review addresses. | Lines 3-4 |
| **METHODS** | | |  |
| Eligibility criteria | 3 | Specify the inclusion and exclusion criteria for the review. | Lines 7-9 |
| Information sources | 4 | Specify the information sources (e.g. databases, registers) used to identify studies and the date when each was last searched. | Lines 6-7 |
| Risk of bias | 5 | Specify the methods used to assess risk of bias in the included studies. | Lines 9-10 |
| Synthesis of results | 6 | Specify the methods used to present and synthesise results. | Lines 10-16 |
| **RESULTS** | | |  |
| Included studies | 7 | Give the total number of included studies and participants and summarise relevant characteristics of studies. | Lines 18-19 |
| Synthesis of results | 8 | Present results for main outcomes, preferably indicating the number of included studies and participants for each. If meta-analysis was done, report the summary estimate and confidence/credible interval. If comparing groups, indicate the direction of the effect (i.e. which group is favoured). | Lines 19-26 |
| **DISCUSSION** | | |  |
| Limitations of evidence | 9 | Provide a brief summary of the limitations of the evidence included in the review (e.g. study risk of bias, inconsistency and imprecision). | Lines 28-30 |
| Interpretation | 10 | Provide a general interpretation of the results and important implications. | Line 28 |
| **OTHER** | | |  |
| Funding | 11 | Specify the primary source of funding for the review. | Lines 31-32 |
| Registration | 12 | Provide the register name and registration number. | Lines 15-16 |

# Supplementary Table S2: Search strategies in databases

Ovid Medline

| 1. | metapneumovirus.mp. |
| --- | --- |
| 2. | exp Metapneumovirus/ |
| 3. | hMPV.mp. |
| 4. | 1 or 2 or 3 |
| 5. | Mortality/ |
| 6. | (mortality or fatality or death*).mp. |
| 7. | (intensive care unit* or intensive treatment unit* or critical care unit*).mp. |
| 8. | exp Intensive Care Units/ |
| 9. | ICU.mp. |
| 10. | exp Hospitalization/ |
| 11. | (hospitali*ation or hospital stay or hospital admission*).mp. |
| 12. | oxygen support.mp. |
| 13. | (oxygen adj2 therapy).mp. or exp Oxygen Inhalation Therapy/ |
| 14. | supplemental oxygen.mp. |
| 15. | oxygen supplementation.mp. |
| 16. | artificial respiration.mp. |
| 17. | exp Respiration, Artificial/ |
| 18. | assisted ventilation.mp. |
| 19. | mechanical ventilation.mp. |
| 20. | exp Morbidity/ |
| 21. | (morbidit*).mp. |
| 22. | incidence.mp. |
| 23. | prevalence.mp. |
| 24. | (disease burden or burden of disease).mp. |
| 25. | exp "Global Burden of Disease"/ |
| 26. | odds ratio/ |
| 27. | risk/ |
| 28. | (hazard* or odd* or risk*).mp. |
| 29. | 5 or 6 or 7 or 8 or 9 or 10 or 11 or 12 or 13 or 14 or 15 or 16 or 17 or 18 or 19 or 20 or 21 or 22 or 23 or 24 or 25 or 26 or 27 or 28 |
| 30. | (cancer or neoplasia or neoplasm or malignanc*).mp. |
| 31. | exp Neoplasms/ |
| 32. | Immunocompromised Host/ |
| 33. | (immunocompromised or immunosuppressed or immunosuppression).mp. |
| 34. | exp HIV/ |
| 35. | HIV.mp. |
| 36. | immunotherapy.mp. |
| 37. | exp Immunotherapy/ |
| 38. | exp Lung Diseases/ |
| 39. | lung disease.mp. |
| 40. | exp Respiratory Tract Diseases/ |
| 41. | exp Cardiovascular Diseases/ |
| 42. | exp Comorbidity/ |
| 43. | (comorbidit* or multimorbidit* or co?morbid or multi?morbid*).mp. |
| 44. | diabetes.mp. |
| 45. | exp Diabetes Mellitus/ |
| 46. | exp Transplantation/ |
| 47. | (transplantation or transplant*).mp. |
| 48. | exp "Cell- and Tissue-Based Therapy"/ |
| 49. | underlying health condition*.mp. |
| 50. | 30 or 31 or 32 or 33 or 34 or 35 or 36 or 37 or 38 or 39 or 40 or 41 or 42 or 43 or 44 or 45 or 46 or 47 or 48 or 49 |
| 51. | exp child/ or exp infant/ |
| 52. | Adult/ |
| 53. | 51 not 52 |
| 54. | exp Respiratory Syncytial Viruses/ |
| 55. | respiratory syncytial virus.mp. |
| 56. | RSV.mp. |
| 57. | 54 or 55 or 56 |
| 58. | 4 and 57 |
| 59. | 58 and 29 and 50 |
| 60. | 59 not 53 |

Ovid Embase

| 1. | exp Metapneumovirus/ |
| --- | --- |
| 2. | metapneumovirus.mp. |
| 3. | exp Metapneumovirus infection/ |
| 4. | hMPV.mp. |
| 5. | 1 or 2 or 3 or 4 |
| 6. | (mortality or fatality or death*).mp. |
| 7. | exp mortality/ |
| 8. | exp intensive care unit/ |
| 9. | (intensive care unit* or intensive treatment unit* or critical care unit*).mp. |
| 10. | ICU.mp. |
| 11. | exp hospitalization/ |
| 12. | (hospitali*ation or hospital stay or hospital admission).mp. |
| 13. | oxygen support.mp. |
| 14. | exp oxygen therapy/ |
| 15. | (oxygen adj2 therapy).mp. |
| 16. | supplemental oxygen.mp. |
| 17. | oxygen supplementation.mp. |
| 18. | artificial respiration.mp. |
| 19. | exp artificial ventilation/ |
| 20. | ventilation.mp. |
| 21. | exp assisted ventilation/ |
| 22. | assisted ventilation.mp. |
| 23. | mechanical ventilation.mp. |
| 24. | exp morbidity/ |
| 25. | (morbidit*). mp. |
| 26. | exp incidence/ |
| 27. | incidence.mp. |
| 28. | exp prevalence/ |
| 29. | prevalence.mp. |
| 30. | exp disease burden/ |
| 31. | (disease burden or burden of disease).mp. |
| 32. | exp global disease burden/ |
| 33. | risk/ |
| 34. | attributable risk/ |
| 35. | risk factor/ |
| 36. | hazard/ |
| 37. | hazard ratio/ |
| 38. | odds ratio/ |
| 39. | 6 or 7 or 8 or 9 or 10 or 11 or 12 or 13 or 14 or 15 or 16 or 17 or 18 or 19 or 20 or 21 or 22 or 23 or 24 or 25 or 26 or 27 or 28 or 29 or 30 or 31 or 32 or 33 or 34 or 35 or 36 or 37 or 38 |
| 40. | exp malignant neoplasm/ |
| 41. | (cancer or neoplasia or neoplasm or malignanc*).mp. |
| 42. | exp immunocompromised patient/ |
| 43. | (immunocompromised or immunosuppress*).mp. |
| 44. | HIV.mp. |
| 45. | exp Human immunodeficiency virus/ |
| 46. | exp immunotherapy/ |
| 47. | immunotherapy.mp. |
| 48. | exp respiratory tract disease/ |
| 49. | respiratory tract disease.mp. |
| 50. | cardiovascular disease.mp. or exp cardiovascular disease/ |
| 51. | exp comorbidity/ |
| 52. | (comorbidit* or multimorbidit* or co?morbid* or multi?morbid*).mp. |
| 53. | diabetes.mp. |
| 54. | exp diabetes mellitus/ |
| 55. | exp transplantation/ or transplant*.mp. |
| 56. | underlying health condition*.mp. |
| 57. | 40 or 41 or 42 or 43 or 44 or 45 or 46 or 47 or 48 or 49 or 50 or 51 or 52 or 53 or 54 or 55 |
| 58. | exp child/ |
| 59. | adult/ |
| 60. | 58 not 59 |
| 61. | exp Human respiratory syncytial virus/ |
| 62. | respiratory syncytial virus.mp. |
| 63. | rsv.mp. |
| 64. | 61 or 62 or 63 |
| 65. | 5 and 64 |
| 66. | 65 and 39 and 57 |
| 67. | 66 not 60 |

Ovid Global Health

| 1. | metapneumovirus.mp. or exp Metapneumovirus/ |
| --- | --- |
| 2. | hMPV.mp. |
| 3. | 1 or 2 |
| 4. | mortality.mp. or exp mortality/ |
| 5. | (fatality or death*).mp. |
| 6. | (intensive care unit* or critical care unit* or intensive treatment unit*).mp. |
| 7. | intensive care units/ |
| 8. | ICU.mp. |
| 9. | hospitali*ation.mp. |
| 10. | hospital admission*.mp. or hospital admission/ |
| 11. | hospital stay/ or hospital stay.mp. |
| 12. | oxygen support.mp. |
| 13. | (oxygen adj2 therapy).mp. |
| 14. | oxygen supplementation.mp. |
| 15. | exp artificial respiration/ |
| 16. | artificial respiration.mp. |
| 17. | ventilation.mp. |
| 18. | exp ventilation/ |
| 19. | (morbidit*).mp. |
| 20. | exp morbidity/ |
| 21. | incidence.mp. |
| 22. | exp incidence/ |
| 23. | disease prevalence.mp. |
| 24. | exp disease prevalence/ |
| 25. | (global burden of disease or disease burden).mp. |
| 26. | (odd* or risk* or hazard*).mp. |
| 27. | cancer.mp. or exp neoplasms/ or neoplasms.mp. or malignanc*.mp. |
| 28. | immunocompromised.mp. or exp immunocompromised hosts/ or immunosuppress*.mp. |
| 29. | HIV.mp. or exp human immunodeficiency viruses/ |
| 30. | immunotherapy.mp. or exp immunotherapy/ |
| 31. | lung disease.mp. or exp respiratory diseases/ |
| 32. | respiratory tract disease.mp. |
| 33. | cardiovascular disease.mp. or exp cardiovascular diseases/ |
| 34. | (comorbidit* or multimorbidit* or co?morbid*).mp.or exp comorbidity/ |
| 35. | diabetes.mp. or exp diabetes mellitus/ |
| 36. | transplant*.mp. or exp transplantation/ |
| 37. | underlying health condition*.mp. |
| 38. | 4 or 5 or 6 or 7 or 8 or 9 or 10 or 11 or 12 or 13 or 14 or 15 or 16 or 17 or 18 or 19 or 20 or 21 or 22 or 23 or 24 or 25 |
| 39. | 27 or 28 or 29 or 30 or 31 or 32 or 33 or 34 or 35 or 36 or 37 |
| 40. | exp children/ |
| 41. | adults/ |
| 42. | 40 not 41 |
| 43. | rsv.mp. |
| 44. | respiratory syncytial virus.mp. |
| 45. | exp human respiratory syncytial virus/ |
| 46. | 43 or 44 or 45 |
| 47. | 3 and 46 |
| 48. | 47 and 38 and 39 |
| 49. | 48 not 42 |

Ebscohost CINAHL

| 1 | (TX metapneumovirus or TX hmpv or TX mpv) and (TX respiratory syncytial virus or rsv) |
| --- | --- |
| 2 | (MH "Mortality") OR (MH "Fatal Outcome") |
| 3 | XB (mortality or fatality or death or deaths) |
| 4 | (MH "Intensive Care Units") |
| 5 | XB (intensive are uni* or critical care uni* or intensive treatment uni*) |
| 6 | (MH "Hospitalization") |
| 7 | XB (hospitalisation or hospitalization or hospital stay or hospital admission or hospital admissions) |
| 8 | (MH "Oxygen Therapy") |
| 9 | XB (oxygen inhalation therapy or oxygen therapy or oxygen support or supplemental oxygen or oxygen supplementation) |
| 10 | (MH "Respiration, Artificial") |
| 11 | XB (artificial respiration or mechanical ventilation or assisted ventilation) |
| 12 | (MH "Morbidity") |
| 13 | XB (morbidit*) |
| 14 | (MH "Incidence") |
| 15 | (MH "Prevalence") |
| 16 | XB (incidence or prevalence) |
| 17 | (MH "Global Burden of Disease") |
| 18 | XB disease burden of burden of disease |
| 19 | (MH "Odds Ratio") |
| 20 | (MH "Risk Management") OR (MH "Relative Risk") OR (MH "Attributable Risk") |
| 21 | XB (odds or hazard or hazards or risk or risks) |
| 22 | S2 OR S3 OR S4 OR S5 OR S6 OR S7 OR S8 OR S9 OR S10 OR S11 OR S12 OR S13 OR S14 OR S15 OR S16 OR S17 OR S18 OR S19 OR S20 OR S21 |
| 23 | (MH "Neoplasms") |
| 24 | (MH "Cancer Patients") |
| 25 | XB (cancer or neoplasia or neoplasm or malignanc*) |
| 26 | (MH "Immunocompromised Host") |
| 27 | XB (immunocompromised or immunosuppressed or immunosuppression) |
| 28 | (MH "Human Immunodeficiency Virus") |
| 29 | XB (hiv or human immunodeficiency virus) |
| 30 | (MH "Immunotherapy") |
| 31 | XB immunotherapy |
| 32 | (MH "Lung Diseases") OR (MH "Lung Diseases, Obstructive") |
| 33 | XB lung disease* |
| 34 | (MH "Respiratory Tract Diseases") |
| 35 | (MH "Cardiovascular Diseases") |
| 36 | (MH "Cardiovascular Diseases") |
| 37 | (MH "Comorbidity") |
| 38 | XB (morbid or morbidit* or co-morbid or co-morbidit* or comorbid or comorbidit* or multimorbid or multimorbidit* or multi-morbid or multi-morbidit*) |
| 39 | (MH "Diabetes Mellitus") |
| 40 | XB diabetes |
| 41 | (MH "Transplantation") |
| 42 | XB (transplant* and transplantation) |
| 43 | XB underlying health condition |
| 44 | S23 OR S24 OR S25 OR S26 OR S27 OR S28 OR S29 OR S30 OR S31 OR S32 OR S33 OR S34 OR S35 OR S36 OR S37 OR S38 OR S39 OR S40 OR S41 OR S42 OR S43 |
| 45 | S1 AND S22 AND S44 |

Global Index Medicus

(tw:(acute respiratory infection or respiratory disease or respiratory tract infection or pneumonia)) AND (tw:(hmpv or mpv or metapneumovirus)) AND (tw:(adult or adults))

Web of Science

| # | Search Query |
| --- | --- |
| 1 | metapneumovirus OR hmpv OR mpv (Topic) |
| 2 | respiratory syncytial virus OR rsv (Topic) |
| 3 | TS=(((hospitali?tion or hospital* or admission* or morbid* or mortal* or death* or prevalen* or inciden* or proportion or oxygen or ventilat* or ((intensive or critical or emergency) NEAR/2 (unit* or room* or facility* or clinic* or treatment)) or ICU or burden* or epidemiolog*) ) ) |
| 4 | TS = (comorbid* or co?morbid* or cancer or neoplasm* or immunocompromise* or immunosuppress* or immunotherapy or hiv or lung disease* or respiratory tract disease* or respiratory disease* or cardiovascular disease* or diabetes or transplant* or underlying health condition) |
| 5 | #1 AND #2 AND #3 AND #4 |
| 6 | TS = adult* |
| 7 | child* (All Fields) OR p*ediatric (Topic) |
| 8 | #7 NOT #6 |
| 9 | #5 NOT #8 |

# Supplementary Text S1: Eligibility criteria for inclusion of studies in the systematic review

*Criteria for including studies:*

- Reporting data on adults (≥18 years) AND;
- Reporting data in people with underlying chronic health conditions (either a specific condition or a group of conditions) AND;
- Reporting a specific case definition or routine surveillance indicating the criteria for inclusion in the study AND;
- Laboratory-confirmed hMPV infections by at least one of the following methods: molecular methods or polymerase chain reaction (PCR), viral culture, immunofluorescence assay (IFA), enzyme-linked immunosorbent assay (ELISA) AND;
- Laboratory-confirmed RSV infections by at least one of the following methods: molecular methods or polymerase chain reaction (PCR), viral culture, immunofluorescence assay (IFA), enzyme-linked immunosorbent assay (ELISA) AND;
- Reporting at least one of the following outcomes:
  - Proportion positive in respiratory illness
  - Incidence
  - Hospital admission rate
  - Intensive care unit (ICU) admission rate
  - Oxygen supplementation rate
  - Case fatality rate AND;
- Prospective or retrospective observational studies, including cross-sectional studies, case-control studies, or cohort studies with a sample size of 100 participants or more or interventional studies (reporting the data for the placebo or control group separately) with a sample size of 100 participants or more (for proportion positive outcome), AND;
- Data collected 2001 onwards

*Criteria for excluding studies:*

- Not reporting data on adults (≥18 years) OR;
- Not reporting data in people with underlying chronic health conditions OR;
- Not reporting specific case definitions or routine surveillance and thus, not indicating the criteria for inclusion in the study, OR;
- Absence of laboratory-confirmed hMPV infection OR;
- Absence of laboratory-confirmed RSV infection OR;
- Not reporting at least one of the following outcomes: Proportion positive in respiratory illness, incidence, hospital admission rate, intensive care unit (ICU) admission rate, oxygen supplementation rate, or case fatality rate, OR;
- Single case reports or case series or studies with a sample size of <100 eligible participants (for proportion positive outcome); or expert opinions or pre-prints, OR;
- Data collection before 2001

# Supplementary Table S3: Classification of chronic underlying health conditions reported in studies into broad groups

| **Chronic health condition group** | **Study** | **Chronic health condition reported in the study** | **Case definition** |
| --- | --- | --- | --- |
| Cancer | Loubet 2021 | Cancer | ILI |
|  | Wee 2025 | Cancer | acute respiratory symptoms |
| Cardiovascular disease | Samoriski 2025 | Congestive heart failure | acute cardiopulmonary illness or ARI |
|  | Samoriski 2025 | Coronary artery disease | acute cardiopulmonary illness or ARI |
|  | Choi 2025 | Chronic heart failure | chronic heart failure exacerbation |
|  | Loubet 2021 | Chronic heart disease | ILI |
|  | Widmer 2014 | Cardiovascular disease | respiratory symptoms |
|  | Widmer 2012 | Cardiovascular disease | RTI |
| Chronic heart disease or lung disease or both | Gorse 2015 | Chronic heart or lung disease or both | ARI |
| Chronic respiratory disease | Samoriski 2025 | Asthma | acute cardiopulmonary illness or ARI |
|  | Samoriski 2025 | COPD | acute cardiopulmonary illness or ARI |
|  | Biancardi 2016 | COPD | AECOPD |
|  | Djamin 2014 | COPD | AECOPD |
|  | McManus 2008 | COPD | AECOPD |
|  | Hosseini 2015 | COPD | AECOPD |
|  | Dimopoulos 2012 | COPD | AECOPD |
|  | Koul 2017 | COPD | AECOPD |
|  | Jang 2021 | COPD | AECOPD |
|  | Kwak 2016 | COPD | AECOPD |
|  | Choi 2025 | COPD | AECOPD |
|  | Seo 2022 | COPD | AECOPD |
|  | Kim 2016 | COPD | AECOPD or CPCOPD |
|  | Ko 2007 | COPD | any COPD GOLD stage |
|  | Gao 2015 | Bronchiectasis | bronchiectasis exacerbation |
|  | Menendez 2017 | Bronchiectasis | bronchiectasis exacerbation |
|  | Loubet 2021 | Chronic respiratory disease | ILI |
|  | Lee 2021 | COPD | moderate-to-severe AECOPD |
|  | Etherington 2014 | Cystic fibrosis | pulmonary exacerbation |
|  | Thornton 2024 | Cystic fibrosis | pulmonary exacerbation |
|  | Widmer 2014 | Pulmonary disease | respiratory symptoms |
|  | Flight 2014 | Cystic fibrosis | routine asymptomatic testing |
|  | Widmer 2012 | Pulmonary disease | RTI |
|  | Seo 2017 | Asthma | suspected RTI (stable LRTI or exacerbated LRTI) |
| Diabetes | Samoriski 2025 | Diabetes mellitus | acute cardiopulmonary illness or ARI |
|  | Loubet 2021 | Diabetes | ILI |
|  | Widmer 2014 | Diabetes mellitus | respiratory symptoms |
|  | Widmer 2012 | Diabetes mellitus | RTI |
| Haematologic diseases | Campbell 2015 | Allogeneic HCT recipients | routine asymptomatic or symptomatic testing |
|  | Jethani 2025 | HSCT | respiratory viral infection |
|  | Salmanton-Garcia 2025 | Haematologic malignancy | RTI |
|  | Kim 2022 * | Pre-autologous and allogeneic HCT | symptomatic RVI |
|  | Lokhandwala 2026 | Haematologic malignancy | physiologically significant RTI |
| Immunocompromised | Dumas 2023 | Immunocompromised | ARF |
|  | Ponsford 2021 | Primary antibody deficiency | fortnightly testing or symptomatic respiratory score exacerbation |
|  | Loubet 2021 | Immunosuppressive therapy | ILI |
|  | Widmer 2014 | Immunodeficiency | respiratory symptoms |
|  | Reckziegel 2020 | Immunocompromised | RTI |
|  | Widmer 2012 | Immunodeficiency | RTI |
|  | Branas 2015 | Immunocompromised | RTI |
| Kidney disease | Samoriski 2025 | Chronic kidney disease | acute cardiopulmonary illness or ARI |
|  | Loubet 2021 | Chronic renal failure | ILI |
| Liver disease | Loubet 2021 | Cirrhosis | ILI |
| Organ transplant | Hopkins 2018 | Lung transplant recipients | ILI |
|  | Samannodi 2021 | Solid organ transplant recipients | patients hospitalised for any reason |
|  | Weinberg 2010 | Lung transplant recipients | RTI |
|  | Peghin 2017 | Lung transplant recipients | RTID |
|  | Gottlieb 2009 | Lung transplant recipients | URTI or LRTI |
| Substance use disorder | Samoriski 2025 | Substance use disorder | acute cardiopulmonary illness or ARI |

AECOPD = Acute Exacerbation of Chronic Obstructive Pulmonary Disease, ARI = Acute Respiratory Infections, ARF = Acute Respiratory Failure, COPD = Chronic Obstructive Pulmonary Disease, CPCOPD = Concomitant Pneumonia in Chronic Obstructive Pulmonary Disease, GOLD = Global Initiative for Chronic Obstructive Lung Disease, HCT = Haematopoietic Cell Transplantation, HSCT = Haematopoietic Stem Cell Transplantation, ILI = Influenza-Like Illness, LRTI = Lower Respiratory Tract Infection, RTI = Respiratory Tract Infections, RTID = Respiratory Tract Infectious Disease, RVI = Respiratory Viral Infection, URTI = Upper Respiratory Tract Infection

* The study examined respiratory viral infections in adults within 90 days before autologous and allogeneic HCT at the Fred Hutchinson Cancer Center. The study did not report the precise underlying indications for transplantation. Given that autologous and allogeneic haematopoietic cell transplantation in adults are predominantly performed for haematologic diseases, the study population was assumed to have an underlying chronic haematologic condition and was classified accordingly for this analysis.

# Supplementary Table S4: Mapping the distribution of chronic underlying health conditions and data collection periods in studies reporting test positivity

| **Comorbidity group** | **Annual** | **Seasonal** | **Both** | **Unclear** | **Total** |
| --- | --- | --- | --- | --- | --- |
| Cancer | 0 | 1 | 0 | 0 | 1 |
| Cardiovascular disease | 1 | 3 | 2 | 0 | 6 |
| Chronic heart disease or lung disease or both | 1 | 0 | 0 | 0 | 1 |
| Chronic respiratory disease | 19 | 6 | 2 | 1 | 28 |
| Diabetes | 1 | 2 | 1 | 0 | 4 |
| Haematologic diseases * | 4 | 1 | 0 | 0 | 5 |
| Immunocompromised | 7 | 2 | 0 | 0 | 9 |
| Kidney disease | 0 | 1 | 1 | 0 | 2 |
| Liver disease | 0 | 1 | 0 | 0 | 1 |
| Organ transplant | 5 | 0 | 0 | 0 | 5 |
| Substance use disorder | 0 | 0 | 1 | 0 | 1 |
| **Total** | 38 | 17 | 7 | 1 | 63 |

* The study Kim 2022 examined respiratory viral infections in adults within 90 days before autologous and allogeneic HCT at the Fred Hutchinson Cancer Center. The study did not report the precise underlying indications for transplantation. Given that autologous and allogeneic haematopoietic cell transplantation in adults are predominantly performed for haematologic diseases, the study population was assumed to have an underlying chronic haematologic condition and was classified accordingly for this analysis.

# Supplementary Table S5: Case definitions used by individual study authors

| **Study** | **Country** | **Chronic underlying health condition** | **Case definition of the chronic underlying health condition** | **Study case definition** | **Complete case definition** |
| --- | --- | --- | --- | --- | --- |
| Akhmedov 2020 | Germany | allogeneic haematopoietic stem transplant recipients | Adult patients who underwent allogeneic stem cell transplantation at Ulm University Hospital between 2005 and 2018. | URTI, LRTI | URTI was defined as a positive RSV or hMPV PCR test from a nasopharyngeal swab with symptoms of upper respiratory tract infection and no pulmonary infiltrates on chest radiograph or CT scan. A probable/presumptive LRTI was defined as per European Conference on Infections in Leukaemia (ECIL-8) recommendation update as symptoms of lower respiratory tract infection, pulmonary infiltrates seen on chest radiograph or CT scan and a positive RSV or hMPV PCR or immunofluorescent assay test from bronchoalveolar lavage. A possible LRTI was defined as symptoms of lower respiratory tract infection with pulmonary infiltrates seen on chest radiograph or CT and a positive RSV or hMPV PCR test from the nasopharyngeal swab in the absence of BAL samples. |
| Biancardi 2016 | Australia | COPD | An audit was conducted to identify the previous diagnosis of COPD or spirometry performed on admission revealing FEV1/FVC <70%. | AECOPD | (i) age >18 years (ii) previous diagnosis of COPD or Spirometry performed on admission revealing FEV1/FVC <70% and (iii) symptoms of AECOPD as per the GOLD definition (worsening of the patient's respiratory symptoms that is beyond normal day-to-day variations and leads to a change in medication).8 The GOLD definition of AECOPD refers to an acute change in one or more of the following cardinal symptoms: an increase in cough frequency/severity, an increase in sputum volume/change in character and an increase in dyspnoea. |
| Branas 2015 | Spain | immunocompromised | unclear | respiratory disease | unclear |
| Campbell 2015 | USA | allogeneic haematopoietic cell transplant recipients | This study was part of a surveillance study among haematopoietic cell transplant recipients followed for 1 year after haematopoietic cell transplant. | weekly virologic surveillance | Weekly virologic surveillance before and up to 100 days post-transplant. |
| Choi 2025 | USA | COPD or congestive heart failure | Participants were interviewed to capture clinical data, and medical chart abstractions were performed. | exacerbation of congestive heart failure or AECOPD | ARI was defined as having at least 1 of the following symptoms within 14 days of presentation: nasal congestion, rhinorrhoea, sore throat, acute or acute-on-chronic cough, sputum production, or dyspnoea and/or wheezing; and admitting diagnoses suggestive of ARI (ie, pneumonia, upper respiratory infection, bronchitis, respiratory illness due to identified or unknown organisms, asthma exacerbation, respiratory distress, and/or respiratory failure). |
| Clark 2015 | United Kingdom | COPD | COPD as identified by discharge International Classification of Disease, 10th edition code classification. All patients had a diagnosis of COPD documented in hospital case notes and general practitioner records and 98% of patients were taking COPD medications (inhaled corticosteroids, bronchodilators and mucolytic agents) at the time of hospitalisation. | acute exacerbation of a chronic cardiorespiratory disease or new onset of acute cardiorespiratory illness | Acute exacerbation of a chronic cardiorespiratory disease or new onset of acute cardiorespiratory illness of <7 days duration; and able to be recruited within 16 h of hospital admission |
| DeSerres 2009 | Canada | COPD | COPD patients were defined by a baseline forced expiratory volume per second (FEV1) less than 70% of the predicted value or chronic bronchitis (cough and sputum ≥3 months/year for ≥2 consecutive years). | AECOPD | Any increase of respiratory symptoms (dyspnea, cough, and sputum) requiring an unscheduled medical visit. |
| Dimopoulos 2012 | Greece | COPD | Confirmed diagnosis of COPD (classified according to GOLD criteria). | AECOPD | An acute in onset event in the natural course of COPD characterized by a change in the patients “baseline” dyspnoea, cough and sputum (beyond the normal day-to-day variation) usually requiring a modification in regular medication of a stable COPD course. |
| Djamin 2014 | the Netherlands | COPD | COPD diagnosis according to the guidelines of the Global initiative for chronic Obstructive Lung Disease. | AECOPD | unclear |
| Dumas 2023 | France | immunocompromised | The study was performed using the database from a multicentric collaborative group specialised in the management of immunocompromised patients, the Groupe de Recherche en Réanimation Respiratoire en Onco-Hématologie (GRRR-OH). | ARF | PaO2 < 60 mmHg and/or SpO2 < 90% on room air and/or tachypnoea > 30/min and/or signs of respiratory distress, such as laboured breathing, and/or the need for more than 6L/min oxygen. |
| Etherington 2014 | UK | cystic fibrosis | Study included adult patients attending the Leeds Regional Adult CF Unit who were commenced on treatment with intravenous antibiotics for an acute pulmonary exacerbation over a 12 month period. | pulmonary exacerbation | unclear |
| Feikin 2012 | Kenya | HIV positive | HIV testing was performed as part of a home-based testing initiative during 2008, when all persons ≥13 years in the surveillance area were offered HIV testing (two parallel rapid HIV tests). It was assumed that a person’s HIV status during home-based testing was the same throughout the study period. HIV-testing was not performed routinely in the clinic on most patients during this period. | ARI | ARI was defined as cough, difficulty breathing or chest pain *and* reported fever. |
| Flight 2014 | UK | cystic fibrosis | Patients aged ≥18 years attending the Manchester Adult Cystic Fibrosis Centre were invited to participate. | routine surveillance or pulmonary exacerbation of upper respiratory tract infection | During follow-up, patients were seen routinely every 2 months. |
| Gao 2015 | China | bronchiectasis | The study included patients with bronchiectasis, diagnosed by a compatible history combined with bronchial dilatation on high-resolution CT (HRCT) scan. | Bronchiectasis exacerbation | persistent (> 24 h) deterioration in at least three respiratory symptoms, including cough, dyspnoea, haemoptysis, increased sputum purulence or volume, chest pain, febrile, radiographic deterioration, systemic disturbances, or changes in chest auscultation. |
| Gorse 2015 | USA | chronic heart or lung disease or both | Chronic heart or lung disease patients were capable of attending outpatient clinics and complying with study procedures, but they were excluded if they had a life expectancy <3 years in the clinical judgment of the investigator, a febrile or respiratory illness within 15 days before enrolment, a significant bleeding disorder, asplenia, or a psychiatric condition that precluded compliance. | ARI | 3 symptoms or fever (body temperature ≥37.8°C) accompanied by 2 symptoms of acute respiratory illness, and kept a daily temperature and symptom diary during the illness. |
| Gottlieb 2009 | Germany | lung transplant recipients | Patients were eligible if they were at least 4 weeks posttransplant and had received a lung transplant, including single, double lung, and combined organs. | URTI, LRTI | Symptoms of URTI and LRTI were recorded by self-rated questionnaires (list of symptoms unavailable). |
| Hong 2014 | Republic of Korea | immunocompromised | At least one of the following conditions: receipt of chemotherapy and/or radiation therapy in the previous 6 months, receipt of corticosteroids (daily administration of corticosteroids at least 5 mg per day of prednisolone or an equivalent drug), receipt of non-steroidal immunosuppressant, haematopoietic stem cell transplantation, or solid organ transplantation. | severe HAP | The presence of a new and persistent radiographic infiltrate occurring 48 hours or more after admission, plus two or more of the following: (i) fever (38.5uC or higher) or hypothermia(,36.5uC); (ii) leucocytosis (white blood cells .10,000/mm3 or ,4,000/mm3); or (iii) purulent tracheal aspirate or sputum. |
| Hopkins 2008 | Australia | lung transplant recipients | unclear | ILI | Any combination of sore throat, nasal irritation, low-grade fever, myalgia, and arthralgia with or without lower respiratory tract symptoms of cough, dyspnoea, or wheeze. |
| Hosseini 2015 | Iran | COPD | unclear | AECOPD | Patients were monitored every day by diary cards, which recorded any increase within the last 48 h in their chronic (i.e. stable) symptoms: ‘major’ (dyspnoea, sputum purulence, and sputum amount) and ‘minor’ (increased nasal discharge/congestion, wheeze, sore throat, and cough). Exacerbations were identified from symptoms listed on the diary cards. The appearance or deterioration of any two of the aforementioned major symptoms, or one major and one minor symptom, occurring within 2 consecutive days were recorded. In regards to the appearance of symptoms, the first day of the 2 consecutive days was called the day of onset of exacerbation. |
| Jang 2021 | South Korea | COPD | unclear | AECOPD | unclear |
| Jethani 2025 | India | haematopoietic stem cell transplant | This cohort study included 100 HSCT recipients >18 years of age, enrolled from January 2017 to February 2020, and followed-up prospectively for 18 months for respiratory episodes until August 2021. HSCT was performed at the Department of Medical Oncology (Dr. Bheem Rao Ambedkar Institute Rotary Cancer Hospital), at All India Institute of Medical Sciences, New Delhi. | respiratory viral infection | unclear |
| Kim 2016 | Republic of Korea | COPD | Diagnosis and severity of COPD were defined by the Global Initiative for Chronic Obstructive Lung Disease (GOLD) guidelines. | AECOPD | AE-COPD was defined clinically by presentation of at least two of the following symptoms; increased shortness of breath, increased sputum volume and purulence with no definite pneumonic infiltration on chest X-ray. CP-COPD is defined as a pneumonia in COPD patients. Pneumonia was defined as the presence of a new radiographic pulmonary infiltration plus two or more of the following; (i) fever (38.5°C or higher) or hypothermia (<36.5°C); (ii) leucocytosis or leukopenia (white blood cells >10,000/mm3 or <4,000/mm3); or (iii) purulent tracheal aspirate or sputum. |
| Kim 2022 | USA | pre-autologous and allogeneic haematopoietic cell transplantation | Adult autologous and allogeneic haematopoietic cell transplant recipients undergoing transplant from March 2010 to March 2016 at the Fred Hutchinson Cancer Center were included. | RVI | RVI status was classified as either URI or LRD.8,19  URI was defined as respiratory infection confined to the nose, throat, and sinuses as confirmed by respiratory virus detection with URI symptoms but no pulmonary infiltrates. LRD was further classified as (modified from prior definitions): (1) possible infection, respiratory virus detection in upper respiratory tract with new pulmonary infiltrates and with LRD signs and symptoms (eg, cough, wheezing, rales, tachypnoea, shortness of breath, dyspnoea, or hypoxia); (2) probable infection, respiratory virus detection in the lung with LRD symptoms without new pulmonary infiltrates; and (3) proven infection, respiratory virus detection in the lung with new pulmonary infiltrates with or without LRD symptoms. We also evaluated the possible infection category of LRD as with or without LRD signs and symptoms. |
| Ko 2007 | Hong Kong | COPD | COPD diagnosis according to Global Initiative for Chronic Obstructive Lung Disease: global strategy for the diagnosis, management and prevention of chronic obstructive pulmonary disease; updated 2004, National Heart, Lung, and Blood Institute, World Health Organization, Bethesda, MD (2004). | AECOPD | A patient with background COPD presented with at least two major symptoms (increased dyspnoea, increased sputum purulence, increased sputum volume) or one major and one minor symptom (nasal discharge/congestion, wheeze, sore throat, cough) for at least 2 consecutive days. |
| Koul 2017 | India | COPD | unclear | AECOPD | AECOPD was defined as ≥2 major symptoms (increased dyspnoea, sputum purulence, or sputum amount) or ≥1 major and ≥1 minor symptom (nasal discharge/congestion, wheezing, sore throat, or cough) for ≥2 consecutive days in a patient with COPD. |
| Kwak 2016 | South Korea | COPD | The presence of  COPD was confirmed based on clinical manifestations and postbronchodilator spirometry (the ratio of forced expiratory volume in 1 second (FEV1) over forced vital capacity (FVC) less than 70%). | AECOPD | Acute change in more than two respiratory symptoms (dyspnoea, cough, and/or sputum) beyond normal day-to-day variation requiring a change in medication for COPD as indicated by the Global Initiative for Obstructive Lung Disease (GOLD). |
| Lee 2021 | South Korea | COPD | COPD was diagnosed following the guidelines of the Global Initiative for Chronic Obstructive Lung Disease (GOLD) employing spirometry, with a ratio of forced expiratory volume in 1 second (FEV1) to forced vital capacity of less than 0.70 after bronchodilator application indicative of persistent airflow limitation. | moderate-to-severe AECOPD | Defined according to GOLD guidelines. |
| Lokhandwala 2026 | Singapore | haematologic malignancy | Eligibility for inclusion was determined through encounter-level International Classification of Diseases (10th Revision)—Clinical Modification codes (ICD-10-CM) and these codes were used to categorise patients into 4 discrete groups: leukaemia, lymphoma, myeloma, and HCT recipients | physiologically significant RTI | Hospitalisations in which patients had a recorded respiratory rate > 20 breaths per minute, peripheral oxygen saturation < 93%, or new use of supplemental oxygen or advanced respiratory support (supplemental oxygen without a concurrent ICD-10-CM code for chronic supplemental oxygen use, or any use of humidified high-flow nasal oxygen [HFNO], noninvasive ventilation [NIV], or invasive mechanical ventilation [IMV]) within the first 5 days of admission. |
| Loubet 2021 | France | chronic respiratory disease, chronic heart disease, diabetes, chronic renal failure, cancer, cirrhosis, immunosuppressant | unclear | ILI | ILI was defined according to the European Centre for Disease Prevention and Control (ECDC) definition as a combination of the following: (a) at least one of the following systemic symptoms – fever or feverishness, headache, myalgia or malaise; and (b) at least one of the following respiratory symptoms – cough, sore throat or dyspnoea. |
| Maillard 2023 | France | immunosuppressed | the use of long-term (> 3 months) or high-dose (> 0.5 mg/kg/d) steroids, use of other immunosuppressant drugs, solid organ transplantation, solid tumor requiring chemotherapy in the last 5 years, hematologic malignancy regardless of time since diagnosis, or primary immune deficiency | ARF | Pao2 < 60 mm Hg or saturation of peripheral oxygen (Spo2) < 90% on room air, or by tachypnoea > 30/min or laboured breathing or respiratory distress and need for oxygen ≥ 6 L/min. |
| McManus 2008 | UK | COPD | COPD and assessment of severity was classified according to the GOLD criteria. | AECOPD | COPD was classified according to the GOLD criteria with symptoms of increased dyspnoea, increased cough or increased sputum production. |
| Menendez 2017 | Spain | bronchiectasis | The diagnosis of bronchiectasis was confirmed by computerised tomography scan of the lungs, along with compatible symptoms, and the aetiology of bronchiectasis had been investigated according to Spanish guidelines previous to study recruitment. | bronchiectasis exacerbation | Acute change in sputum characteristics (increased volume, change of viscosity, purulence) with or without increased dyspnoea after ruling out any other causes along with the requirement of a new antibiotic treatment prescribed in our specific clinic and / or unscheduled admission to hospital, the study also included new chest -ray infiltrates diagnosed as pneumonia. |
| Murphy 2013 | Australia | asthma (in pregnant women) | Women with asthma (had a doctor's diagnosis of asthma and asthma symptoms or therapy in the prior 3 months) and were between 12 and 20 weeks' gestation, who were > 18 years of age, were recruited from April 2007 to November 2009 at the antenatal clinic of John Hunter Hospital, Newcastle, Australia. | common cold | unclear |
| Park 2013 | South Korea | haematologic diseases | Record review of the microbiology laboratory admitted to the Asan Medical Center, a 2,700-bed tertiary-care hospital in Seoul, South Korea, from January 2009 to February 2012. Diagnoses included: acute myeloid leukaemia, acute lymphoblastic leukaemia, chronic myeloid leukaemia, non-Hodgkin lymphoma, other. | haematologic diseases | Upper respiratory infection was defined as detection of viruses in upper respiratory secretions, along with symptoms involving the nose and throat. Lower respiratory infection was defined as the presence of either hypoxia or pulmonary infiltrates, along with identification of viruses in upper or lower respiratory secretions. |
| Peghin 2017 | Spain | lung transplant recipients | All consecutive adult patients undergoing lung transplantation at Hospital Univeristari Vall d’Hebron (Barcelona, Spain) from September 2009 to September 2011. | respiratory tract infectious disease | An upper respiratory tract infectious disease (URTID) was defined as an illness caused by an acute infection with the onset of sore throat, rhinorrhoea or hoarseness. A lower respiratory tract infectious disease (LRTID) was defined as new onset of shortness of breath, cough, sputum, rales, hypoxemia and/or wheezing. When symptoms of LRTID were associated with a new pulmonary infiltrate (on chest radiograph or chest computed tomography), pneumonia was distinguished from tracheobronchitis. Nosocomial RV infectious disease refers to any infectious disease contracted by a patient in a hospital at least 48–72 h after being admitted. |
| Piñana 2020 | Spain | allogeneic haematopoietic stem cell transplant recipients | This was a study of allogeneic haematopoietic stem cell transplant recipients conducted at two Spanish transplant centres in Valencia, Spain. The study analysed all consecutive molecularly-proven community-acquired respiratory viral infectious episodes occurring during the first year after allogeneic haematopoietic stem cell transplant. | URTD or LRTD | URTD was defined as a combination of upper respiratory symptoms (rhinorrhoea, sinusitis, otitis, or pharyngitis) as well as positive CARV diagnosis by PCR test in respiratory samples, and absence of LRTD symptoms and/or any indication of pulmonary infiltrates in chest X-ray or CT scan radiology results. |
| Ranchow 2020 | Germany | allogeneic stem cell transplant recipients | Patients were recruited in the outpatient clinic of the stem cell trans-plantation program during routine visits | testing during routine visits | Testing conducted at random time points |
| Ponsford 2021 | UK | primary antibody deficiency | Patients were recruited from the Immunodeficiency Centre for  Wales, Cardiff, if they had a diagnosis of primary antibody deficiency and had commenced  immunoglobulin replacement for >3 months, with trough  IgG level greater than or equal to 5 g/L. | fortnightly routine surveillance or symptomatic respiratory exacerbation | Following instruction, patients with PAD performed nasal swabbing every 2 weeks over a 12-month period; symptomatic respiratory exacerbation (SRE) was defined by a symptom score of 2 or more occurring for 2 or more consecutive days as recorded by the patient. |
| Reckziegel 2020 | Germany | immunocompromised | A compromised immune status was defined 1) for solid  organ or stem cell transplant recipients under iatrogenic  immunosuppression, 2) in patients with autoimmune  disorders under immunosuppressant therapy, but also  3) in cancer patients under chemotherapy/radiation,  and 4) in patients with primary or secondary causes  of immunodeficiency, including human immunodeficiency virus infection. | RTI | Common cold, cough with/without sputum, dyspnoea, and fever. |
| Samannodi 2021 | USA | solid organ transplant recipients | All adult solid organ transplant  recipients who were hospitalised for any reason and had the first  nasopharyngeal viral polymerase chain reaction post transplantation. | patient hospitalised for any reason | Patient hospitalised for any reason and had the first nasopharyngeal viral polymerase chain reaction post transplantation. |
| Samoriski 2025 | USA | COPD, asthma, coronary artery disease, congestive heart failure, diabetes mellitus, chronic kidney disease, substance use disorder and dementia | At enrolment, medical history was collected from medical records and patient and family interviews. | acute cardiopulmonary illness or ARI | Symptoms of acute cardiopulmonary illness (difficulty breathing, cough, wheezing, chest pain) or admission diagnoses compatible with ARI (ie, pneumonia, acute exacerbations of chronic obstructive pulmonary disease or asthma, bronchitis, upper respiratory infection, influenza, or viral syndrome) were screened. |
| Seo 2017 | South Korea | asthma | Asthma was previously diagnosed based on the Global Initiative for Asthma guidelines (GINA report: global strategy for asthma management and prevention 2011 May 4.  All subjects had a clinical diagnosis of asthma supported by at least one of the following criteria: 1) an increase in the forced expiratory  volume in 1 second (FEV1) of >12% or 200 mL after inhalation  of 400 µg albuterol, 2) a reduction in the FEV1 of 20% in response to a provocative concentration of <10 mg/mL inhaled  methacholine (PC20), and 3) an increase in the FEV1 >20%  over 14 days after inhaled or systemic corticosteroid use. | suspected RTI | Respiratory tract infections were suspected if the common symptoms of upper respiratory tract illnesses (URTIs), such as cough, sore throat, runny nose, post-nasal drip, nasal congestion, and low-grade fever, or those of LRTIs, such as shortness of breath, weakness, fever, coughing, sputum production, and wheezing were present  An exacerbation was diagnosed when pre-existing dyspnoea and wheezing became aggravated within 14 days before the study, together with a post-bronchodilator FEV1 <80% of the personal best.18 We divided them into exacerbated LRTI and stable LRTI. |
| Seo 2022 | South Korea | COPD | COPD diagnosis confirmed by a pulmonary function test (post-bronchodilator forced expiratory volume in 1 second (FEV1)/forced vital capacity < 0.7) performed within 6 months before the onset of AECOPD. | AECOPD | Acute exacerbation of COPD (AECOPD) is defined as an acute worsening of respiratory symptoms that require additional treatment. |
| Spahr 2018 | Switzerland | allogeneic haematopoietic cell transplant | At the University Hospital Basel, Switzerland, allogeneic HCT recipients were identified between June 2010 and December 2014 | RTID | [Upper and lower RTID was defined as described by the recent ECIL-4 guidelines [7]: upper RTID was defined as virus detection in upper respiratory secretions, together with symptoms involving the upper respiratory tract (nose and throat); lower RTID was defined as the presence of either hypoxia or compatible pulmonary infiltrates, together with virus detection in upper or lower respiratory secretions](javascript:;). |
| Thornton 2024 | USA, Canada | cystic fibrosis | STOP2 (NCT02781610) was a multicentre,  prospective randomised controlled clinical  trial conducted in the United States and  Canada from July 2016 through January  2020 in adults with cystic fibrosis diagnosed with pulmonary exacerbation  for which intravenous antimicrobials were prescribed. | pulmonary exacerbation | Increased cough and sputum production and acute loss of lung function and weight, among other signs and symptoms; and for which IV antimicrobials were prescribed. |
| Wee 2025 | Singapore | cancer | Patients who were hospitalized in SGH under the Department of Hematology and the oncology wards managed by the National Cancer Centre, Singapore were included. | acute respiratory symptoms | unclear |
| Weinberg 2010 | USA | lung transplant recipients | A total of 60 lung transplant recipients who resided in Denver and were followed at the University of Colorado Denver  consented to enrol in the prospective study to determine  the incidence of hMPV and other community-acquired respiratory viruses in new onset RTI. | RTI | Subjects with signs and symptoms suggestive of upper RTI, such as rhinorrhoea, sore throat, or cough, underwent nasal washes; those whose signs and symptoms suggested lower RTI, including wheezing, a >10% fall in forced expiratory volume in 1 s (FEV1), shortness of breath, or oxygen desaturation. |
| Widmer 2012 | USA | cardiovascular disease, pulmonary disease, diabetes mellitus, immunodeficiency | Medical record review captured comorbidities. | RTI | Any respiratory symptoms (ie, cough, nasal congestion, coryza, dyspnoea, or wheezing) or nonlocalising fever. |
| Widmer 2014 | USA | cardiovascular disease, pulmonary disease, diabetes mellitus, immunodeficiency | Medical record review captured comorbidities. | respiratory symptoms | Patients were eligible if they had any respiratory symptoms (i.e., cough, nasal congestion, coryza, dyspnoea or wheezing) or non-localizing fever that who underwent allogeneic stem cell transplantation at Ulm University Hospital between 2005 and 2018had begun within 7 days prior to presentation. |
| Yin 2017 | China | COPD | COPD were diagnosed according to GOLD criteria, with a postbronchodilator forced expiratory volume in 1 second (FEV1) to forced vital capacity (FVC) ratio <70%. | AECOPD | An acute event characterized by a worsening of respiratory symptoms (dyspnoea, sputum purulence or sputum volume) that was beyond normal day-to-day variations, and led to a change in medication |

AECOPD = Acute Exacerbation of Chronic Obstructive Pulmonary Disease, ARF = acute respiratory failure, ARI = Acute Respiratory Infections, COPD = Chronic Obstructive Pulmonary Disease, HAP = Hospital-Acquired Pneumonia, HCT = Haematopoietic Cell Transplantation, HIV = Human Immunodeficiency Virus,, ILI = influenza-like illness, LRTD = Lower Respiratory Tract Disease, LRTI = Lower Respiratory Tract Infection, RTI = Respiratory Tract Infection, RTID = Respiratory Tract Infectious Disease, RVI = Respiratory Viral Infection, URTD = Upper Respiratory Tract Disease, URTI = Upper Respiratory Tract Infection

# Supplementary Table S6: List of studies excluded at the full text screening stage, with the reason for exclusion

| **Title** | **Authors** | **Published Year** | **Reason for exclusion** |
| --- | --- | --- | --- |
| Comorbidities associated with respiratory viral infections in Egypt in non intensive care unit cases. single site experience/cairo university hospitals from 2010 to 2014 | Aboelhassan, U. E.; Hatem, A. M.; Abdelwahab, S. M.; Abdelwahab, M. S.; Elkholy, A. | 2019 | abstract only |
| Outcomes of patients with severe acute respiratory infections (SARI) admitted to the intensive care unit: Results from the Egyptian surveillance study 2010-2014 | Abu Elhassan, U. E.; Mohamed, S. A. A.; Rizk, M. S.; Sherif, M.; El-Harras, M. | 2020 | wrong population (no comorbidities) |
| Epidemiology of respiratory viral infections in two long-term refugee camps in Kenya, 2007-2010 | Ahmed, Jamal A.; Katz, Mark A.; Auko, Eric; Njenga, M. Kariuki; Weinberg, Michelle; Kapella, Bryan K.; Burke, Heather; Nyoka, Raymond; Gichangi, Anthony; Waiboci, Lilian W.; Mahamud, Abdirahman; Qassim, Mohamed; Swai, Babu; Wagacha, Burton; Mutonga, David; Nguhi, Margaret; Breiman, Robert F.; Eidex, Rachel B. | 2012 | wrong population (wrong age group) |
| Coinfections with Respiratory Syncytial Virus (RSV) in a Cohort of Adults with Pre-Existing Comorbidities in Wisconsin from 2015-16 through 2019-20 | Alonge, O.; Sundaram, M.; Nguyen, H.; King, J. P.; Stefanski, E.; Saeedi, P.; Brabant, Y.; Pircon, J. Y. | 2025 | abstract only |
| Mixed viral infections of the respiratory tract; an epidemiological study during consecutive winter seasons | Antalis, E.; Oikonomopoulou, Z.; Kottaridi, C.; Kossyvakis, A.; Spathis, A.; Magkana, M.; Katsouli, A.; Tsagris, V.; Papaevangelou, V.; Mentis, A.; Tsiodras, S. | 2018 | wrong outcomes |
| Upper and lower respiratory tract infections by respiratory viruses in adult recipients of allogeneic hematopoietic stem cell transplantation (allo-HSCT) | Atilla, E.; Ataca, P.; Sahin, D.; Topcuoglu, P.; Dolapci, I.; Tekeli, A.; Toprak, S. K.; Bozdag, S. C.; Yuksel, M. K.; Ozcan, M.; Gurman, G. | 2015 | abstract only |
| Non-influenza respiratory viruses in adult patients admitted with influenza-like illness: a 3-year prospective multicenter study | BÃ©nÃ©zit, FranÃ§ois; Loubet, Paul; Galtier, Florence; Pronier, Charlotte; Lenzi, Nezha; Lesieur, Zineb; Jouneau, StÃ©phane; Lagathu, GisÃ¨le; L'Honneur, Anne-Sophie; Foulongne, Vincent; Vallejo, Christine; Alain, Sophie; Duval, Xavier; Houhou, Nawal; Costa, Yolande; Vanhems, Philippe; Amour, SÃ©lilah; Carrat, Fabrice; Lina, Bruno; Launay, Odile | 2020 | wrong outcomes |
| Respiratory viral infections in patients with chronic, obstructive pulmonary disease | Beckham, J. D.; Cadena, A.; Lin, J.; Piedra, P. A.; Glezen, W. P.; Greenberg, S. B.; Atmar, R. L. | 2005 | wrong data collection period |
| Asymptomatic and symptomatic respiratory virus infection detected in naso-pharyngeal swabs from solid organ transplant recipients early after transplantation | BenMarzouk-Hidalgo, O. J.; Molina, J.; Cordero, E.; Merino, L.; Cabello, V.; Suarez-Artacho, G.; Sobrino, M.; Perez-Romero, P. | 2011 | wrong outcomes |
| Community acquired respiratory viral infection after lung transplantation: BOS risk and risks of specific viruses | Benzimra, M.; Ainge-Allen, H. W.; Rigby, A. L.; Malouf, M. A.; Havryk, A. P.; Plit, M.; Glanville, A. R. | 2014 | abstract only |
| The burden of RSV, hMPV, and PIV amongst hospitalized adults in the United States from 2016 to 2019 | Bhasin, A.; Nguyen, D. C.; Briggs, B. J.; Nam, H. H. | 2024 | wrong or unclear case definition |
| Etiology of community-acquired pneumonia in patients with chronic heart failure | Bobylev, A. A.; Rachina, S. A.; Avdeev, S. N.; Sukhorukova, M. V.; Yatsyshina, S. B.; Omar, S.; Zorya, O. T.; Solovjeva, A. E. | 2019 | abstract only |
| Progression, shedding patterns, and clinical disease associated with respiratory virus infections after allogeneic hematopoietic cell transplantation (HCT) | Boeckh, M.; Campbell, A.; Xie, H.; Kuypers, J.; Leisenring, W. M.; Chien, J.; Jerome, K.; Englund, J. A. | 2013 | abstract only |
| Comparative analysis of mortality in patients admitted with an infection with influenza A/B virus, respiratory syncytial virus, Rhinovirus, Metapneumovirus or SARS-CoV-2 | Boon, Hanneke; Meinders, Arend-Jan; van Hannen, Erik Jan; Tersmette, Matthijs; Schaftenaar, Erik | 2024 | wrong outcomes |
| Predictive Value of Respiratory Viral Detection in the Upper Respiratory Tract for Infection of the Lower Respiratory Tract With Hematopoietic Stem Cell Transplantation | Boonyaratanakornkit, Jim; Vivek, Meghana; Xie, Hu; Pergam, Steven A.; Cheng, Guang-Shing; Mielcarek, Marco; Hill, Joshua A.; Jerome, Keith R.; Limaye, Ajit P.; Leisenring, Wendy; Boeckh, Michael J.; Waghmare, Alpana | 2020 | wrong population (wrong age group) |
| Respiratory viral infections in Western Australians with cystic fibrosis | Brestovac, B.; Lawrence, C.; Speers, D. J.; Sammels, L. M.; Mulrennan, S. | 2020 | <100 specimens tested |
| Viral pathogens in acute exacerbations of chronic obstructive pulmonary disease | Camargo, Carlos A., Jr.; Ginde, Adit A.; Clark, Sunday; Cartwright, Charles P.; Falsey, Ann R.; Niewoehner, Dennis E. | 2008 | <100 specimens tested |
| Virus infection in exacerbations of chronic obstructive pulmonary disease requiring ventilation | Cameron, R. J.; de Wit, D.; Welsh, T. N.; Ferguson, J.; Grissell, T. V.; Rye, P. J. | 2006 | wrong data collection period |
| Respiratory virus pneumonia after hematopoietic cell transplantation (HCT): Associations between viral load in bronchoalveolar lavage samples, viral RNA detection in serum samples, and clinical outcomes of HCT | Campbell, A. P.; Chien, J. W.; Kuypers, J.; Englund, J. A.; Wald, A.; Guthrie, K. A.; Corey, L.; Boeckh, M. | 2010 | wrong data collection period |
| Survey of Respiratory Virus in Patients Hospitalised for Acute Exacerbations of Heart Failure - A Prospective Observational Study | Candice-Yy, Chan; Jenny-Gh, L. O. W.; Wyiki, Wyone; Lynette, Le Oon; Ban-Hock, T. A. N. | 2018 | wrong population (no comorbidities) |
| Viral pneumonia studied at university hospital in Buenos Aires | Carpio, M. | 2019 | abstract only |
| A virologic survey of patients admitted to a critical care unit for acute cardiorespiratory failure | Carrat, Fabrice; Leruez-Ville, Marianne; Tonnellier, Marc; Baudel, Jean-Luc; Deshayes, Juliette; Meyer, Pascal; Maury, Eric; Galimand, Julie; Rouzioux, Christine; Offenstadt, Georges | 2006 | wrong population (wrong age group) |
| The Clinical Characteristics and Outcomes of Adult Patients With Pneumonia Related to Three Paramyxoviruses | Chen, L.; Han, X. D.; Li, Y. L.; Zhang, C. X.; Xing, X. Q. | 2021 | wrong outcomes |
| Clinical characteristics and outcomes in adult patients hospitalized with influenza, respiratory syncytial virus and human metapneumovirus infections | Chen, L.; Han, X.; Bai, L.; Zhang, J. | 2021 | <100 specimens tested |
| Bacterial and Viral Identification Rate in Acute Exacerbation of Chronic Obstructive Pulmonary Disease in Korea | Choi, J.; Oh, J. Y.; Lee, Y. S.; Hur, G. Y.; Lee, S. Y.; Shim, J. J.; Kang, K. H.; Min, K. H. | 2019 | wrong outcomes |
| Viruses of respiratory tract: an observational retrospective study on hospitalized patients in Rome, Italy | Ciotti, Marco; Maurici, Massimo; Santoro, Viviana; Coppola, Luigi; Sarmati, Loredana; de Carolis, Gerardo; de Filippis, Patrizia; Pica, Francesca | 2020 | wrong outcomes |
| Viral pathogens associated with acute respiratory illness in hospitalized adults and elderly from Zagreb, Croatia, 2016 to 2018 | Civljak, R.; Tot, T.; Falsey, A. R.; Huljev, E.; Vranes, J.; Ljubin-Sternak, S. | 2019 | wrong outcomes |
| Antibiotic Use and Respiratory Pathogens in Adults With Sickle Cell Disease and Acute Chest Syndrome | Claudio, A. M.; Foltanski, L.; Delay, T.; Britell, A.; Duckett, A.; Weeda, E. R.; Bohm, N. | 2019 | no hMPV or RSV detected |
| Severe COVID-19 pneumonia in an intensive care setting and comparisons with historic severe viral pneumonia due to other viruses | Dadhwal, K.; Stonham, R.; Breen, H.; Poole, S.; Saeed, K.; Dushianthan, A. | 2022 | wrong outcomes |
| Respiratory infectious phenotypes in acute exacerbation of COPD: an aid to length of stay and COPD Assessment Test | Dai, Meng-Yuan; Qiao, Jin-Ping; Xu, Yuan-Hong; Fei, Guang-He | 2015 | <100 specimens tested |
| Incidence and predictors of respiratory viral infections by multi-plex pcr in allogeneic hematopoietic cell transplant (HCT) recipients 50 years and older including geriatric assessment (GA) | D'Angelo, C. R.; Kocherginsky, M.; Bishop, M. R.; Godley, L. A.; Kline, J.; Larson, R. A.; Liu, H.; Odenike, O.; Pisano, J.; Stock, W.; Artz, A. | 2014 | abstract only |
| Incidence and predictors of respiratory viral infections by multiplex PCR in allogeneic hematopoietic cell transplant recipients 50 years and older including geriatric assessment | D'Angelo, C. R.; Kocherginsky, M.; Pisano, J.; Bishop, M. R.; Godley, L. A.; Kline, J.; Larson, R. A.; Liu, H.; Odenike, O.; Stock, W.; Artz, A. S. | 2016 | no hMPV or RSV detected |
| Common seasonal respiratory virus infections in allogeneic stem cell transplant recipients during the SARS-COV-2 pandemic | De la Puerta, R.; Montoro, J.; Aznar, C.; Lorenzo, I.; Gonzalez-Barbera, E. M.; Balaguer-Rosello, A.; Guerreiro, M.; Dominguez, L.; Salavert, M.; Aguilar, C.; de la Rubia, J.; Sanz, J.; Gomez, M. D.; Pinana, J. L. | 2021 | wrong population (wrong age group) |
| Respiratory viral infections in patients with lymphoma and multiple myeloma: Risk factors associated with progression to lower respiratory tract infection and mortality | De La Rosa Martinez, D.; Vilar-Compte, D.; Batista, M. V.; Khawaja, F.; El Haddad, L.; Ariza-eredia, E. J.; Chemaly, R. F. | 2019 | abstract only |
| Prolonged respiratory viral shedding in transplant patients | De Lima, C. R. A.; Mirandolli, T. B.; Carneiro, L. C.; Tusset, C.; Romer, C. M.; Andreolla, H. F.; Baethgen, L. F.; Pasqualotto, A. C. | 2014 | <100 specimens tested |
| Evaluation of 10 years of parainfluenza virus, human metapneumovirus, and respiratory syncytial virus infections in lung transplant recipients | de Zwart, Auke E. S.; Riezebos-Brilman, Annelies; Alffenaar, Jan-Willem C.; van den Heuvel, Edwin R.; Gan, Christiaan Tji; van der Bij, Wim; Kerstjens, Huib A. M.; Verschuuren, Erik A. M. | 2020 | wrong or unclear case definition |
| Clinical Outcome of Viral Respiratory Tract Infections in Hospitalized Adults in Norway: High Degree of Inflammation and Need of Emergency Care for Cases With Respiratory Syncytial Virus | Debes, S.; Haug, J. B.; de Blasio, B. F.; Lindstrom, J. C.; Jonassen, C. M.; Dudman, S. G. | 2022 | wrong or unclear case definition |
| Impact of respiratory viruses detection on outcomes in ventilated nosocomial pneumonia: an exposed/unexposed study | Do Rego, Hermann; Dessajan, Julien; Le Hingrat, Quentin; Lefevre, Laurence Armand; De Montmollin, Etienne; Thy, Michael; Ruckly, Stephane; Sonneville, Romain; Bouadma, Lila; Grall, Nathalie; Timsit, Jean-Francois | 2025 | wrong outcomes |
| Human Metapneumovirus Infection in Adults and Its Role in Differential Diagnosis of COVID-19 | Dogan, L.; Uyar, N. Y.; Kocagoz, S. | 2025 | wrong outcomes |
| Detection of respiratory syncytial virus and human metapneumovirus by reverse transcription polymerase chain reaction in adults with and without respiratory illness | Falsey, A. R.; Criddle, M. C.; Walsh, E. E. | 2006 | wrong outcomes |
| Risk Factors and Medical Resource Utilization of Respiratory Syncytial Virus, Human Metapneumovirus, and Influenza-Related Hospitalizations in Adults-A Global Study During the 2017-2019 Epidemic Seasons (Hospitalized Acute Respiratory Tract Infection [HARTI] Study) | Falsey, A. R.; Walsh, E. E.; House, S.; Vandenijck, Y.; Ren, X.; Keim, S.; Kang, D.; Peeters, P.; Witek, J.; Ispas, G. | 2021 | wrong outcomes |
| Comparative assessment of reported symptoms of influenza, respiratory syncytial virus, and human metapneumovirus infection during hospitalization and post-discharge assessed by Respiratory Intensity and Impact Questionnaire | Falsey, A. R.; Walsh, E. E.; Osborne, R. H.; Vandendijck, Y.; Ren, X.; Witek, J.; Kang, D.; Chan, E.; Scott, J.; Ispas, G. | 2022 | wrong population (no comorbidities) |
| Assessment of Illness Severity in Adults Hospitalized With Acute Respiratory Tract Infection due to Influenza, Respiratory Syncytial Virus, or Human Metapneumovirus | Falsey, A.; Walsh, E.; House, S.; Vandendijck, Y.; Stevens, M.; Chan, E.; Ispas, G. | 2024 | wrong outcomes |
| Infectious exacerbation of chronic obstructive pulmonary disease: place and role of respiratory viral pathogens | Feshchenko, Y. I.; Gavrisyuk, V. K.; Dziublyk, I. V.; Dziublyk, O. Y.; Gumeniuk, G. L.; Gumeniuk, M. I.; Kapitan, G. B.; Yachnik, V. A. | 2019 | <100 specimens tested |
| Burden of community-acquired respiratory virus infections and impact on cancer treatment at a tertiary care cancer center in Japan | Fujita, T.; Iida, T. | 2025 | wrong or unclear case definition |
| Single-centre experience with oral ribavirin in lung transplant recipients with paramyxovirus infections | Fuehner, T.; Dierich, M.; Duesberg, C.; DeWall, C.; Welte, T.; Haverich, A.; Warnecke, G.; Simon, A. R.; Gottlieb, J. | 2011 | wrong population (wrong age group) |
| Lower respiratory viral illnesses - Improved diagnosis by molecular methods and clinical impact | Garbino, J.; Gerbase, M. W.; Wunderli, W.; Deffernez, C.; Thomas, Y.; Rochat, T.; Ninet, B.; Schrenzel, J.; Yerly, S.; Perrin, L.; Soccal, P. M.; Nicod, L.; Kaiser, L. | 2004 | wrong outcomes |
| Clinical Outcomes of Paramyxovirus Infections in Lung Transplant Recipients Treated With Oral Ribavirin: A Two-Center Case Series | Garcia, B.; Sharma, N.; Johnson, K.; Salgado, J.; Wille, K. | 2019 | wrong outcomes |
| Disease burden of the most commonly detected respiratory viruses in hospitalized patients calculated using the disability adjusted life year (DALY) model | Gaunt, E. R.; Harvala, H.; McIntyre, C.; Templeton, K. E.; Simmonds, P. | 2011 | wrong outcomes |
| Impact of human metapneumovirus and human cytomegalovirus versus other respiratory viruses on the lower respiratory tract infections of lung transplant recipients | Gerna, Giuseppe; Vitulo, Patrizio; Rovida, Francesca; Lilleri, Daniele; Pellegrini, Carlo; Oggionni, Tiberio; Campanini, Giulia; Baldanti, Fausto; Revello, M. Grazia | 2006 | wrong population (wrong age group) |
| Epidemiological trends of respiratory tract pathogens detected via mPCR in Australian adult patients before COVID-19 | Grech, A. K.; Foo, C. T.; Paul, E.; Aung, A. K.; Yu, C. S. A. | 2024 | wrong outcomes |
| Human metapneumovirus-associated severe acute respiratory illness hospitalisation in HIV-infected and HIV-uninfected South African children and adults | Groome, M. J.; Moyes, J.; Cohen, C.; Walaza, S.; Tempia, S.; Pretorius, M.; Hellferscee, O.; Chhagan, M.; Haffejee, S.; Dawood, H.; Kahn, K.; Variava, E.; Cohen, A. L.; von Gottberg, A.; Wolter, N.; Venter, M.; Madhi, S. A. | 2015 | wrong outcomes |
| Frequency and significance of human rhinovirus and coronavirus detection in high risk patients | Guaring-Angulo, N.; Grosser, D. S.; Rezaei, A.; Watson, W.; Montgomery, M.; Dhiman, N. | 2015 | abstract only |
| Clinical study concerning the relationship between community-acquired pneumonia and viral infection in northern Thailand | Hara, K.; Yahara, K.; Gotoh, K.; Nakazono, Y.; Kashiwagi, T.; Imamura, Y.; Hamada, N.; Khositsakulchai, W.; Sanchai, T.; Khantawa, B.; Tharavichitkul, P.; Maneekarn, N.; Sirisanthana, T.; Watanabe, H. | 2011 | wrong population (wrong age group) |
| Clinical phenotypes and outcomes of SARS-CoV-2, influenza, RSV and seven other respiratory viruses: A retrospective study using complete hospital data | Hedberg, P.; Karlsson Valik, J.; Van Der Werff, S.; Tanushi, H.; Requena Mendez, A.; Granath, F.; Bell, M.; Martensson, J.; Dyrdak, R.; Hertting, O.; Farnert, A.; Ternhag, A.; Naucler, P. | 2022 | wrong outcomes |
| Clinical phenotypes and outcomes of SARS-CoV-2, influenza, RSV and seven other respiratory viruses: a retrospective study using complete hospital data | Hedberg, Pontus; Valik, John Karlsson; van der Werff, Suzanne; Tanushi, Hideyuki; Mendez, Ana Requena; Granath, Fredrik; Bell, Max; Martensson, Johan; Dyrdak, Robert; Hertting, Olof; Farnert, Anna; Ternhag, Anders; Naucler, Pontus | 2021 | wrong outcomes |
| Asthma, chronic obstructive pulmonary disease, respiratory tract infections and viruses: Admission rates, emergency room visits and age | Ho, T.; Corriveau, S.; Johnston, N.; Greene, J.; Killian, K.; O'Byrne, P. M. | 2017 | abstract only |
| Lower respiratory tract diseases caused by common respiratory viruses among stem cell transplantation recipients: A single center experience in Korea | Hong, K. W.; Choi, S. M.; Lee, D. G.; Cho, S. Y.; Lee, H. J.; Choi, J. K.; Kim, S. H.; Park, S. H.; Choi, J. H.; Yoo, J. H.; Lee, J. W. | 2017 | wrong outcomes |
| Viral etiology, seasonality and severity of hospitalized patients with severe acute respiratory infections in the Eastern Mediterranean Region, 2007-2014 | Horton, K. C.; Dueger, E. L.; Kandeel, A.; Abdallat, M.; El-Kholy, A.; Al-Awaidy, S.; Kohlani, A. H.; Amer, H.; ElKhal, A. L.; Said, M.; House, B.; Pimentel, G.; Talaat, M. | 2017 | wrong population (wrong age group) |
| Viral Respiratory Tract Infections in Adult Patients Attending Outpatient and Emergency Departments, Taiwan, 2012-2013: A PCR/Electrospray Ionization Mass Spectrometry Study | Hsin, I. Shih; Hsuan-Chen, Wang; Ih-Jen, Su; Hsiang-Chin, Hsu; Jen-Ren, Wang; Hsiao Fang Sunny, Sun; Chien-Hsuan, Chou; Wen-Chien, Ko; Ming, I. Hsieh; Chi-Jung, Wu; Shih, Hsin- I.; Wang, Hsuan-Chen; Su, Ih-Jen; Hsu, Hsiang-Chin; Wang, Jen-Ren; Sun, Hsiao Fang Sunny; Chou, Chien-Hsuan; Ko, Wen-Chien; Hsieh, Ming- I.; Wu, Chi-Jung | 2015 | wrong outcomes |
| Sputum pathogen spectrum and clinical outcomes of upper respiratory tract infection in bronchiectasis exacerbation: a prospective cohort study | Huang, Y.; Chen, C. L.; Cen, L. J.; Li, H. M.; Lin, Z. H.; Zhu, S. Y.; Duan, C. Y.; Zhang, R. L.; Pan, C. X.; Zhang, X. F.; Zhang, X. X.; He, Z. F.; Shi, M. X.; Zhong, N. S.; Guan, W. J. | 2023 | <100 specimens tested |
| [Several Common Respiratory Viral Pathogens in Hematopoietic Stem Cell Transplantion Patients with Primary Immunodeficiency Disease] | Huang, Yu; Chen, Zhi; Meng, Yan; Guan, Xian-Min; Yu, Jie; Zhao, Xiao-Dong; Dou, Ying | 2020 | wrong population (wrong age group) |
| The importance of bacterial and viral infections associated with adult asthma exacerbations in clinical practice | Iikura, M.; Hojo, M.; Koketsu, R.; Watanabe, S.; Sato, A.; Chino, H.; Ro, S.; Masaki, H.; Hirashima, J.; Ishii, S.; Naka, G.; Takasaki, J.; Izumi, S.; Kobayashi, N.; Yamaguchi, S.; Nakae, S.; Sugiyama, H. | 2015 | <100 specimens tested |
| Economic and clinical burden associated with respiratory viral infections after allogeneic hematopoietic cell transplant in the United States | Ison, M. G.; Marty, F. M.; Chao, N.; Moon, S. H.; Zhang, Z.; Chandak, A. | 2022 | wrong population (wrong age group) |
| Respiratory viral infection in immunocompromised patients | Jahn, K.; Schumann, D.; Tamm, M.; Hirsch, H.; Halter, J.; Junker, L.; Strobel, W.; Savic, S.; Stolz, D. | 2018 | abstract only |
| High prevalence of human metapneumovirus subtype B in cases presenting as severe acute respiratory illness: An experience at tertiary care hospital | Jain, B.; Singh, A. K.; Dangi, T.; Agarwal, A.; Verma, A. K.; Dwivedi, M.; Singh, K. P.; Jain, A. | 2014 | wrong outcomes |
| Clinical Significance of Various Pathogens Identified in Patients Experiencing Acute Exacerbations of COPD: A Multi-center Study in South Korea | Ji, H. W.; Yu, S.; Sim, Y. S.; Seo, H.; Park, J. W.; Min, K. H.; Kim, D. K.; Lee, H. W.; Rhee, C. K.; Park, Y. B.; Shin, K. C.; Yoo, K. H.; Jung, J. Y. | 2025 | duplicate data |
| Viral infection in adults hospitalized with community-acquired pneumonia: Prevalence, pathogens, and presentation | Johnstone, J.; Majumdar, S. R.; Fox, J. D.; Marrie, T. J. | 2008 | wrong outcomes |
| Risk of respiratory viral infections after hematopoietic stem cell transplantation in Indian patients | Jyoti, J.; Sameer, S.; Prashant, K.; Lalit, D. | 2021 | wrong population (wrong age group) |
| Serum inflammatory biomarkers and clinical outcomes of COPD exacerbation caused by different pathogens | Kawamatawong, T.; Apiwattanaporn, A.; Siricharoonwong, W. | 2017 | <100 specimens tested |
| Surveillance of respiratory virus infections in adult hospital admissions using rapid methods | Kaye, M.; Skidmore, S.; Osman, H.; Weinbren, M.; Warren, R. | 2006 | wrong population (no comorbidities) |
| Clinical decision making is improved by BioFire Pneumonia Plus in suspected lower respiratory tract infection after lung transplantation: Results of the prospective DBATE-IT* study | Kayser, M. Z.; Seeliger, B.; Valtin, C.; Fuge, J.; Ziesing, S.; Welte, T.; Pletz, M. W.; Chhatwal, P.; Gottlieb, J. | 2022 | wrong population (wrong age group) |
| Upper-respiratory viral infection, biomarkers, and COPD exacerbations | Kherad, Omar; Kaiser, Laurent; Bridevaux, Pierre-Olivier; Sarasin, Francois; Thomas, Yves; Janssens, Jean-Paul; Rutschmann, Olivier T. | 2010 | <100 specimens tested |
| Influenzavirus infection is a primary cause of febrile respiratory illness in HIV-infected adults, despite vaccination | Klein, M. B.; Lu, Y.; DelBalso, L.; Cote, S.; Boivin, G. | 2007 | wrong outcomes |
| Respiratory viral testing and antibacterial treatment in patients hospitalized with community-acquired pneumonia | Klompas, M.; Imrey, P. B.; Yu, P. C.; Rhee, C.; Deshpande, A.; Haessler, S.; Zilberberg, M. D.; Rothberg, M. B. | 2021 | wrong outcomes |
| A 1-year prospective study of the infectious etiology in patients hospitalized with acute exacerbations of COPD | Ko, F. W. S.; Ip, M.; Chan, P. K. S.; Fok, J. P. C.; Chan, M. C. H.; Ngai, J. C.; Chan, D. P. S.; Hui, D. S. C. | 2007 | wrong outcomes |
| Comparison of the frequency of viral infections in patients with inborn errors of immunity receiving immunoglobulin by different routes | Kose, H.; Ozkan, G.; Simsek, A.; Karali, Y.; Saglik, I.; Agca, H.; Kilic, S. S. | 2025 | wrong population (wrong age group) |
| Clinical impact of community-acquired respiratory viruses on bronchiolitis obliterans after lung transplant | Kumar, D.; Erdman, D.; Keshavjee, S.; Peret, T.; Tellier, R.; Hadjiliadis, D.; Johnson, G.; Ayers, M.; Siegal, D.; Humar, A. | 2005 | wrong population (wrong age group) |
| A prospective molecular surveillance study evaluating the clinical impact of community-acquired respiratory viruses in lung transplant recipients | Kumar, D.; Husain, S.; Chen, M. H.; Moussa, G.; Himsworth, D.; Manuel, O.; Studer, S.; Pakstis, D.; McCurry, K.; Doucette, K.; Pilewski, J.; Janeczko, R.; Humar, A. | 2010 | wrong population (wrong age group) |
| Incidence, risk factors, and viral etiology of community-acquired acute lower respiratory tract infection among older adults in rural north India | Kumar, R.; Dar, L.; Amarchand, R.; Saha, S.; Lafond, K. E.; Purakayastha, D. R.; Kumar, R.; Choudekar, A.; Gopal, G.; Dhakad, S.; Narayan, V. V.; Wahi, A.; Chhokar, R.; Lindstrom, S.; Whitaker, B.; Choudhary, A.; Dey, A. B.; Krishnan, A. | 2021 | wrong population (no comorbidities) |
| Occurrence and disease burden of respiratory syncytial virus and other respiratory pathogens in adults aged >=65 years in community: a prospective cohort study in Japan | Kurai, Daisuke; Natori, Makiko; Yamada, Maho; Zheng, Ri-chuan; Saito, Yuki; Takahashi, Hiroshi | 2021 | wrong outcomes |
| ETIOLOGY OF INFLUENZA-LIKE ILLNESSES IN THE POPULATION OF NOVOSIBIRSK CITY IN THE 2018-2019 EPIDEMIC SEASON | Kurskaya, O. G.; Anoshina, A. V.; Leonova, N. V.; Simkina, O. A.; Komissarova, T. V.; Esikova, E. Y.; Pozdnyakova, L. L.; Sobolev, I. A.; Prokopyeva, E. A.; Kazachkova, E. A.; Danilenko, D. M.; Komissarov, A. B.; Stolyarov, K. A.; Fadeev, A. V.; Sominina, A. A.; Sharshov, K. A.; Yu, A. A.; Shestopalov, A. M.; Murashkina, T. A. | 2021 | wrong outcomes |
| The Effect of Respiratory Viral Infections on Breakthrough Hemolysis in Patients with Paroxysmal Nocturnal Hemoglobinuria | Lazana, I.; Apap Mangion, S.; Babiker, S.; Large, J.; Trikha, R.; Zuckerman, M.; Gandhi, S.; Kulasekararaj, A. G. | 2023 | wrong outcomes |
| Characteristics of community-acquired respiratory viruses infections except seasonal influenza in transplant recipients and non-transplant critically ill patients | Lee, Kyoung Hwa; Yoo, Seul Gi; Cho, Yonggeun; Kwon, Da Eun; La, Yeonju; Han, Sang Hoon; Kim, Myoung Soo; Choi, Jin Sub; Kim, Soon Il; Kim, Yu Seun; Min, Yoo Hong; Cheong, June-Won; Kim, Jin Seok; Song, Yong Goo | 2021 | wrong population (wrong age group) |
| Burden and Clinical Impact of Non-Influenza Respiratory Viral (NIRV) Infections among Hospitalized Adults | Lee, N.; Mubareka, S.; Zapernick, L.; Bekking, C.; Labib, M.; Waldner, D.; Zelyas, N.; Smith, S. | 2019 | abstract only |
| Burden of noninfluenza respiratory viral infections in adults admitted to hospital: analysis of a multiyear Canadian surveillance cohort from 2 centres | Lee, Nelson; Smith, Stephanie; Zelyas, Nathan; Klarenbach, Scott; Zapernick, Lori; Bekking, Christian; So, Helen; Yip, Lily; Tipples, Graham; Taylor, Geoff; Mubareka, Samira | 2021 | wrong population (no comorbidities) |
| Viral etiology of severe acute respiratory infections in hospitalized patients, Shandong, China | Li, C.; He, Y.; Pan, X.; Yin, H.; Pei, Y.; Song, S.; Sun, L.; Zhang, S.; Wu, J.; Li, Z.; Wang, X.; Kou, Z.; Zhao, L.; Xing, W.; Liu, T. | 2025 | wrong population (no comorbidities) |
| Etiological and epidemiological characteristics of severe acute respiratory infection caused by multiple viruses and Mycoplasma pneumoniae in adult patients in Jinshan, Shanghai: a pilot hospital-based surveillance study | Li, Jian; Song, Can-Lei; Wang, Tang; Ye, Yu-Long; Du, Jian-Ru; Li, Shu-Hua; Zhu, Jian-Min | 2021 | wrong population (no comorbidities) |
| Clinical characteristics and prognostic risk factors of mortality in patients with interstitial lung diseases and viral infection: a retrospective cohort study | Li, L. J.; Wang, C. L.; Sun, L. X.; Zhang, X. Q.; Yang, G. R. | 2021 | wrong outcomes |
| Characteristics of viral pneumonia in non-HIV immunocompromised and immunocompetent patients: a retrospective cohort study | Li, L.; Hsu, S. H.; Wang, C.; Li, B.; Sun, L.; Shi, J.; Ren, Y.; Wang, J.; Zhang, X.; Liu, J. | 2021 | wrong outcomes |
| Viral etiologies and epidemiology of patients with acute respiratory infections based on sentinel hospitals in Gansu Province, Northwest China, 2011-2015 | Li, Xuechao; Li, Juansheng; Meng, Lei; Zhu, Wanqi; Liu, Xinfeng; Yang, Mei; Yu, Deshan; Niu, Lixia; Shen, Xiping | 2018 | wrong population (no comorbidities) |
| Impact of viral infection on acute exacerbation of asthma in out-patient clinics: A prospective study | Liao, H.; Yang, Z.; Yang, C.; Tang, Y.; Liu, S.; Guan, W.; Chen, R. | 2016 | wrong population (wrong age group) |
| Etiological analysis and predictive diagnostic model building of community-acquired pneumonia in adult outpatients in Beijing, China | Liu, Y. F.; Gao, Y.; Chen, M. F.; Cao, B.; Yang, X. H.; Wei, L. | 2013 | wrong outcomes |
| Characteristics of Human Metapneumovirus Infection Compared to Respiratory Syncytial Virus and Influenza Infections in Adults Hospitalized for Influenza-Like Illness in France, 2012-2022 | Loubet, P.; Guitton, S.; Rolland, S.; Lefrancois, L. H.; Nguyen, L. B. L.; Vanhems, P.; Laine, F.; Galtier, F.; Duval, X.; Lina, B.; Valette, M.; Lagathu, G.; Foulongne, V.; Houhou-Fidhou, N.; L'Honneur A, S.; Carrat, F.; Meyer, L.; Durier, C.; Launay, O. | 2025 | wrong outcomes |
| Epidemiology of respiratory pathogen carriage in the homeless population within two shelters in Marseille, France, 2015-2017: cross sectional 1-day surveys | Ly, T. D. A.; Edouard, S.; Badiaga, S.; Tissot-Dupont, H.; Hoang, V. T.; Pommier de Santi, V.; Brouqui, P.; Raoult, D.; Gautret, P. | 2019 | wrong population (no comorbidities) |
| Role of Polymerase Chain Reaction-Based Diagnosis of Respiratory Viruses in Febrile Neutropenic Patients | Madhuravasal Krishnan, Janani; Jayaraman, Dhaarani; Kancharla, Adarsh; Thangam, Aishwarya; Venkatramanan, Padmasani; Scott, Julius Xavier | 2023 | wrong population (wrong age group) |
| Characteristics and outcomes among patients with community-acquired respiratory virus infections during the first year after lung transplantation | Mahan, L. D.; Kanade, R.; Mohanka, M. R.; Bollineni, S.; Joerns, J.; Kaza, V.; Torres, F.; La Hoz, R. M.; Banga, A. | 2021 | wrong population (wrong age group) |
| Clinical Features of Respiratory Viral Infections Among Inpatients at a Major US Tertiary Care Hospital | Malhotra, Prashant; Luka, Arthur; McWilliams, Carla S.; Poeth, Kaitlin G.; Schwartz, Rebecca; Elfekey, Mohammed; Balwan, Sandy | 2016 | wrong outcomes |
| Respiratory Viruses Cause Late Morbidity in Recipients of Hematopoietic Stem Cell Transplantation | Marinelli, T.; Wee, L. A.; Rowe, E.; Chhetri, R.; Friel, O.; Higgins, G.; Bardy, P.; Singhal, D.; Pradhan, A.; Crawford, L.; Hiwase, D. K. | 2020 | wrong population (wrong age group) |
| Human metapneumovirus and exacerbations of chronic obstructive pulmonary disease | Martinello, R. A.; Esper, F.; Weibel, C.; Ferguson, D.; Landry, M. L.; Kahn, J. S. | 2006 | <100 specimens tested |
| Prospective study of the incidence, clinical features, and outcome of symptomatic upper and lower respiratory tract infections by respiratory viruses in adult recipients of hematopoietic stem cell transplants for hematologic malignancies | Martino, Rodrigo; Porras, Rocio Parody; Rabella, Nuria; Williams, John V.; Ramila, Elena; Margall, Nuria; Labeaga, Rosa; Crowe, James E., Jr.; Coll, Pedro; Sierra, Jorge | 2005 | wrong data collection period |
| Respiratory Viruses in Febrile Neutropenic Patients with Respiratory Symptoms | Meidani, Mohsen; Mirmohammad Sadeghi, Seyed Alireza | 2018 | wrong population (wrong age group) |
| A single-season prospective study of respiratory viral infections in lung transplant recipients | Milstone, A. P.; Brumble, L. M.; Barnes, J.; Estes, W.; Loyd, J. E.; Pierson, R. N.; Dummer, S. | 2006 | wrong data collection period |
| Seasonal Patterns of Respiratory Syncytial Virus, Influenza A Virus, Human Metapneumovirus, and Parainfluenza Virus Type 3 Infections on the Basis of Virus Isolation Data between 2004 and 2011 in Yamagata, Japan | Mizuta, K.; Abiko, C.; Aoki, Y.; Ikeda, T.; Matsuzaki, Y.; Itagaki, T.; Katsushima, F.; Katsushima, Y.; Noda, M.; Kimura, H.; Ahiko, T. | 2013 | wrong population (no comorbidities) |
| Burden, epidemiology, and outcomes of microbiologically confirmed respiratory viral infections in solid organ transplant recipients: a nationwide, multi-season prospective cohort study | Mombelli, M.; Lang, B. M.; Neofytos, D.; Aubert, J. D.; Benden, C.; Berger, C.; Boggian, K.; Egli, A.; Soccal, P. M.; Kaiser, L.; Hirzel, C.; Pascual, M.; Koller, M.; Mueller, N. J.; van Delden, C.; Hirsch, H. H.; Manuel, O. | 2021 | wrong population (wrong age group) |
| Characterization of Factors Associated With Disease Severity in Adults Hospitalized With Respiratory Viral Infection: The Universal Study | Morelli, T. G.; Purcell, M.; Cox, O.; Lee, P.; Thorne, K.; Roberts, C.; Cazaly, A.; Herbert, W.; Tilt, E.; Allen, A.; Goss, V.; Nuttall, J.; Pavitt, M.; Siddiqui, S.; Greening, N. J.; Crooks, M. G.; Marciniak, S. J.; Daneshvar, C. J.; Myerson, J. S.; Rodrigues, P.; Clark, T.; Freeman, A.; Wilkinson, T. | 2024 | abstract only |
| Characteristics of respiratory virus infections in autologous hematopoietic stem cell transplantation patients, a prospective study, Bern, Switzerland, 2015-2017 | Moret, F.; Marschall, J.; Atkinson, A.; Farag, S.; Zimmerli, S.; Pabst, T.; Sommerstein, R. | 2021 | no hMPV or RSV detected |
| Outcomes of intravenous immunoglobulin treatment of immunocompromised patients with viral respiratory infections | Moughames, E.; Sakayan, S.; Prichett, L.; Runken, M. C.; Borst, D.; Tversky, J.; Azar, A. | 2025 | wrong population (wrong age group) |
| Observational Study on the Clinical Reality of Community-Acquired Respiratory Virus Infections in Adults and Older Individuals | Nagasawa, M.; Udagawa, T.; Kato, T.; Tanaka, I.; Yamamoto, R.; Sakaguchi, H.; Sekikawa, Y. | 2024 | wrong outcomes |
| Laboratory surveillance of influenza-like illness in seven teaching hospitals, South Korea: 2011-2012 season | Noh JiYun, Noh JiYun; Song JoonYoung, Song JoonYoung; Cheong HeeJin, Cheong HeeJin; Choi WonSuk, Choi WonSuk; Lee, J.; Lee JinSoo, Lee JinSoo; Wie SeongHeon, Wie SeongHeon; Jeong HyeWon, Jeong HyeWon; Kim YoungKeun, Kim YoungKeun; Choi SungHyuk, Choi SungHyuk; Han SeungBaik, Han SeungBaik; So ByungHak, So ByungHak; Kim Hyun, Kim Hyun; Kim WooJoo, Kim WooJoo | 2013 | wrong outcomes |
| Respiratory syncytial virus and influenza hospitalizations in Alaska native adults | Nolen, L. D.; Seeman, S.; Desnoyers, C.; DeByle, C.; Klejka, J.; Bruden, D.; Rudolph, K.; Gerber, S. I.; Kim, L.; Langley, G.; Patel, M.; Englund, J.; Chu, H. Y.; Tiesinga, J.; Singleton, R. | 2020 | wrong outcomes |
| Effectiveness of oral ribavirin in immunocompromised adults with respiratory viral infections | Oger, C.; Lefebure, A.; Martelli, S.; Brugiere, O.; Lhuillier, E.; Arnaud, P. | 2017 | abstract only |
| Antibiotic Exposure Prior to Respiratory Viral Infection Is Associated with Progression to Lower Respiratory Tract Disease in Allogeneic Hematopoietic Cell Transplant Recipients | Ogimi, C.; Krantz, E. M.; Golob, J. L.; Waghmare, A.; Liu, C.; Leisenring, W. M.; Woodard, C. R.; Marquis, S.; Kuypers, J. M.; Jerome, K. R.; Pergam, S. A.; Fredricks, D. N.; Sorror, M. L.; Englund, J. A.; Boeckh, M. | 2018 | <100 specimens tested |
| Correlation of initial upper respiratory tract viral burden with progression to lower tract disease in adult allogeneic hematopoietic cell transplant recipients | Ogimi, C.; Xie, H.; Waghmare, A.; Jerome, K. R.; Leisenring, W. M.; Milano, F.; Englund, J. A.; Boeckh, M. | 2022 | wrong outcomes |
| Novel factors to predict respiratory viral disease progression in allogeneic hematopoietic cell transplant recipients | Ogimi, C.; Xie, H.; Waghmare, A.; Jerome, K. R.; Leisenring, W. M.; Ueda Oshima, M.; Carpenter, P. A.; Englund, J. A.; Boeckh, M. | 2022 | wrong outcomes |
| Respiratory virology and microbiology in intensive care units: A prospective cohort study | Ostby, A. C.; Gubbels, S.; Baake, G.; Nielsen, L. P.; Riedel, C.; Arpi, M. | 2013 | wrong outcomes |
| Respiratory Viruses in Acute Exacerbations of Bronchiectasis | Park, Y. E.; Sung, H.; Oh, Y. M. | 2021 | wrong population (wrong age group) |
| Respiratory virus infection among hematopoietic cell transplant recipients: evidence for asymptomatic parainfluenza virus infection | Peck, Angela J.; Englund, Janet A.; Kuypers, Jane; Guthrie, Katherine A.; Corey, Lawrence; Morrow, Rhoda; Hackman, Robert C.; Cent, Anne; Boeckh, Michael | 2007 | wrong population (wrong age group) |
| Sex differences in acute respiratory tract infections-multi-year analysis based on data from a large tertiary care medical center in Israel | Peer, V.; Mandelboim, M.; Jurkowicz, M.; Green, M. S. | 2025 | wrong population (no comorbidities) |
| Are any specific respiratory viruses more severe than others in recipients of allogeneic stem cell transplantation? A focus on lower respiratory tract disease | Perez, A.; Gomez, D.; Montoro, J.; Chorao, P.; Hernani, R.; Guerreiro, M.; Villalba, M.; Albert, E.; Carbonell-Asins, J. A.; Hernandez-Boluda, J. C.; Navarro, D.; Solano, C.; Pinana, J. L. | 2024 | wrong population (wrong age group) |
| Invasive fungal infections after respiratory viral infections in lung transplant recipients are associated with lung allograft failure and chronic lung allograft dysfunction within 1 year | Permpalung, N.; Liang, T.; Gopinath, S.; Bazemore, K.; Mathew, J.; Ostrander, D.; Durand, C. M.; Shoham, S.; Zhang, S. X.; Marr, K. A.; Avery, R. K.; Shah, P. D. | 2023 | wrong population (wrong age group) |
| Incidence and outcomes of hospital-associated respiratory virus infections by viral species | Petrie, J. G.; Moore, R.; Lauring, A. S.; Kaye, K. S. | 2024 | wrong or unclear case definition |
| Epidemiologic and clinical characteristics of coronavirus and bocavirus respiratory infections after allogeneic stem cell transplantation: a prospective single-center study | PiÃ±ana, J. L.; Madrid, S.; PÃ©rez, A.; HernÃ¡ndez-Boluda, J. C.; GimÃ©nez, E.; Terol, M. J. | 2018 | wrong outcomes |
| The clinical benefit of instituting a prospective clinical community-acquired respiratory virus surveillance program in allogeneic hematopoietic stem cell transplantation | PiÃ±ana, J.; Montoro, J.; Aznar, C.; Lorenzo, I.; GÃ³mez, M. D.; Guerreiro, M.; Carretero, C.; GonzÃ¡lez-BarberÃ¡, E. M.; Balaguer-RosellÃ³, A.; Sanz, R.; Salavert, M.; Navarro, D.; Sanz, M. A.; Sanz, G.; Sanz, J. | 2020 | duplicate data |
| Community-Acquired Respiratory Virus Infections: A Threat to Long-Term survivors after Allogeneic Stem Cell Transplant? | Pinana, J. L.; Carbonell-Asins, J. A.; Gomez, D.; Montoro, J.; Perez, A.; Hernani, R.; Chorao, P.; Hernandez-Boluda, J. C.; Navarro, D.; Solano, C. | 2024 | wrong population (wrong age group) |
| Characterizing Respiratory Virus Infections during the Peri-engraftment Period of Allogeneic Hematopoietic Cell Transplant | Pinana, J. L.; Martinez-Lopez, C.; Chorao, P.; Perez, A.; Gomez, D.; Sanz, J.; de la Asuncion, C. S.; Hernandez-Boluda, J. C.; Navarro, D.; Montoro, J.; Solano, C. | 2025 | wrong population (wrong age group) |
| Frequency of respiratory viruses among patients admitted to 26 Intensive Care Units in seven consecutive winter-spring seasons (2009-2016) in Northern Italy | Piralla, A.; Mariani, B.; Rovida, F.; Baldanti, F. | 2017 | wrong outcomes |
| The role of influenza, RSV and other common respiratory viruses in severe acute respiratory infections and influenza-like illness in a population with a high HIV sero-prevalence, South Africa 2012-2015 | Pretorius, M. A.; Tempia, S.; Walaza, S.; Cohen, A. L.; Moyes, J.; Variava, E.; Dawood, H.; Seleka, M.; Hellferscee, O.; Treurnicht, F.; Cohen, C.; Venter, M. | 2016 | wrong outcomes |
| Viral Infections Associated to Patients with Hematologic Malignancies and Hematopoietic Cell Transplant Recipients | Purba, R. H. P.; Asriati, A. E. R. | 2020 | abstract only |
| Comparison of Healthcare Resource Utilization and Disease Outcomes in Adults Hospitalized with Human Metapneumovirus and Respiratory Syncytial Virus | Rayens, E.; Sy, L. S.; Qian, L.; Ackerson, B. K.; An, J.; Luo, Y.; Huang, X.; Ku, J. H.; Modha, P. P.; Bathala, R. M.; Venkatesan, S.; Glasser, L.; Molnar, D.; McNulty, R.; Wang, C.; Tseng, H. F. | 2025 | wrong or unclear case definition |
| Mortality rates of human Metapneumovirus and respiratory syncytial virus lower respiratory tract infections in hematopoietic cell transplantation recipients | Renaud, C.; Xie, H.; Seo, S.; Kuypers, J.; Cent, A.; Corey, L.; Leisenring, W.; Boeckh, M.; Englund, J. A. | 2013 | wrong population (wrong age group) |
| Respiratory virus infections in adult patients hospitalized in an internal medicine unit | Riquelme, R.; Rioseco, M. L.; Agueero, Y.; Ubilla, D.; Mechsner, P.; Inzunza, C.; Riquelme, M. | 2014 | wrong outcomes |
| Respiratory viruses in exacerbations of chronic obstructive pulmonary disease requiring hospitalisation: a case-control study | Rohde, G.; Wiethege, A.; Borg, I.; Kauth, M.; Bauer, T. T.; Gillissen, A.; Bufe, A.; Schultze-Werninghaus, G. | 2003 | wrong data collection period |
| Respiratory Viruses in Patients With Hematological Malignancy in Boreal Autumn/Winter 2023-2024: EPICOVIDEHA-EPIFLUEHA Report | Salmanton-Garcia, J.; Marchesi, F.; Navratil, M.; Piukovics, K.; del Principe, M. I.; Criscuolo, M.; Bilgin, Y. M.; Fracchiolla, N. S.; Vena, A.; Romano, A.; Falces-Romero, I.; Sgherza, N.; Heras-Fernando, I.; Biernat, M. M.; Petzer, V.; Zak, P.; Weinbergerova, B.; Samarkos, M.; Erben, N.; van Praet, J.; Lopez-Garcia, A.; Labrador, J.; Lahmer, T.; Drgona, L.; Merelli, M.; Cuccaro, A.; Martin-Perez, S.; Davila-Valls, J.; Farina, F.; Cattaneo, C.; Pinczes, L. I.; Magyari, F.; Espigado, I.; Buquicchio, C.; Vinh, D. C.; Stoma, I.; Cernan, M.; Prezioso, L.; Papa, M. V.; Plantefeve, G.; Khedr, R. A.; Batinic, J.; Magliano, G.; Erdem, S.; Khostelidi, S.; Colovic, N.; Nappi, D.; Garcia-Ramirez, P.; Gora, J.; Callejas-Charavia, M.; Tlusty, J.; Bakker, M.; Wojtyniak, E.; Antic, D.; Magdziak, A.; Dargenio, M.; Idrizovic, L.; Pantic, N.; Stojanoski, Z.; Eisa, N.; Otasevic, V.; Marchetti, M.; Mackenzie, E.; Garcia-Vidal, C.; Aujayeb, A.; Almasari, A.; Miranda-Castillo, C.; Gavriilaki, E.; Coppola, N.; Busca, A.; Adzic-Vukicevic, T.; Schonlein, M.; Hersby, D. S.; Grafe, S. K.; Glenthoj, A.; Aiello, T. F.; Cvetanoski, M.; Mitrovic, M.; Cerchione, C.; Prin, R.; Varricchio, G.; Arellano, E.; Cordoba, R.; Mayer, J.; Visek, B.; Wolf, D.; Anastasopoulou, A. N.; Delia, M.; Musto, P.; Leotta, D.; Bavastro, M.; Limongelli, A.; Sciume, M.; van den Ven, L.; Fianchi, L.; Brunetti, S. C.; Drozd-Sokolowska, J.; Dabrowska-Iwanicka, A.; Cornely, O. A.; Pagano, L. | 2025 | wrong or unclear case definition |
| Clinical significance of respiratory virus detection in patients with acute exacerbation of interstitial lung diseases | Saraya, T.; Kimura, H.; Kurai, D.; Tamura, M.; Ogawa, Y.; Mikura, S.; Sada, M.; Oda, M.; Watanabe, T.; Ohkuma, K.; Inoue, M.; Honda, K.; Watanabe, M.; Yokoyama, T.; Fujiwara, M.; Ishii, H.; Takizawa, H. | 2018 | <100 specimens tested |
| Prevalence and contribution of respiratory viruses in the community to rates of emergency department visits and hospitalizations with respiratory tract infections, chronic obstructive pulmonary disease and asthma | Satia, I.; Cusack, R.; Greene, J. M.; O'Byrne, P. M.; Killian, K. J.; Johnston, N. | 2020 | wrong outcomes |
| Rhinovirus and other respiratory viruses exert different effects on lung allograft function that are not mediated through acute rejection | Sayah, D. M.; Koff, J. L.; Leard, L. E.; Hays, S. R.; Golden, J. A.; Singer, J. P. | 2013 | wrong population (wrong age group) |
| Molecular detection of respiratory viruses in immunocopromised ICU patients: Incidence and meaning | Schnell, D.; Legoff, J.; Mariotte, E.; Seguin, A.; Canet, E.; Lemiale, V.; Darmon, M.; Schlemmer, B.; Simon, F.; Azoulay, E. | 2012 | wrong population (wrong age group) |
| The burden of respiratory viral illness in HIV-infected patients | Sellers, S. A.; Dover, K.; Wohl, D.; Miller, M.; Dittmer, D.; Fischer, W. | 2017 | abstract only |
| Etiology and clinical outcomes of acute respiratory virus infection in hospitalized adults | Seo, Y. B.; Song, J. Y.; Choi, M. J.; Kim, I. S.; Yang, T. U.; Hong, K. W.; Cheong, H. J.; Kim, W. J. | 2014 | no hMPV or RSV detected |
| Respiratory viral infections in bone marrow transplant patients: insights from a tertiary care hospital in Rawalpindi, Pakistan | Shakoor, N.; Niazi, S. K.; Ghani, E.; Iftikhar, R.; Rathore, A.; Noor, M. | 2024 | wrong population (wrong age group) |
| Burden and Seasonality of Viral Acute Respiratory Tract Infections among Outpatients in Southern Sri Lanka | Shapiro, David; Bodinayake, Champica K.; Nagahawatte, Ajith; Devasiri, Vasantha; Kurukulasooriya, Ruvini; Hsiang, Jeremy; Nicholson, Bradley; De Silva, Aruna Dharshan; Ostbye, Truls; Reller, Megan E.; Woods, Christopher W.; Tillekeratne, L. Gayani | 2017 | wrong population (no comorbidities) |
| Preliminary exploratory analysis of non-influenza respiratory viral infections (NIRVIS) among inpatients at a tertiary teaching hospital in newyork | Sharma, D.; Khalil, A.; Avula, A. | 2018 | abstract only |
| Adaptation of a Russian population to SARS-CoV-2: Asymptomatic course, comorbidities, mortality, and other respiratory viruses - A reply to Fear versus Data | Sharov, K. S. | 2020 | wrong outcomes |
| Respiratory viruses are the most common cause of lower respiratory tract infection in southern province, Sri Lanka | Sheng, T.; Vanderburg, S.; Bodinayake, C.; Wijayaratne, G.; Nagahawatte, A.; Devasiri, V.; Kurukulasooriya, R.; Sellathurai, M.; Danthanarayana, N.; Halloluwa, C.; Sewwandi, K.; Anderson, J. G.; Nicholson, B. P.; Woods, C. W.; Tillekeratne, L. G. | 2020 | abstract only |
| Seasonality and Co-Detection of Respiratory Viral Infections Among Hospitalised Patients Admitted With Acute Respiratory Illness-Valencia Region, Spain, 2010-2021 | Shirreff, G.; Chaves, S.; Coudeville, L.; Mengual-Chulia, B.; Mira-Iglesias, A.; Puig-Barbera, J.; Orrico-Sanchez, A.; Diez-Domingo, J.; Opatowski, L.; Lopez-Labrador, F. | 2024 | wrong population (no comorbidities) |
| Comparative incidence and burden of respiratory viruses associated with hospitalization in adults in New York City | Sieling, W. D.; Goldman, C. R.; Oberhardt, M.; Phillips, M.; Finelli, L.; Saiman, L. | 2021 | wrong population (no comorbidities) |
| Human Metapneumovirus, Respiratory Syncytial Virus and Influenza Associated Pneumonia Hospitalizations in Colorado Adults Aged Over 50 Years: 2016-2023 | Simoes, E. A. F.; Suss, R. J.; Raje, D. V. | 2025 | no lab confirmed diagnosis |
| Clinical Impact of Community-Acquired Respiratory Viruses in Patients With Solid Organ Transplants | Singh, S.; Josan, E.; Kovacs, C. | 2024 | wrong population (wrong age group) |
| Impact of virulent viral pathogens on hospital length of stay and readmissions after an acute exacerbation of chronic obstructive pulmonary disease | Snyder, M. E.; Aaron, C. P.; Regalbuto, R.; Yip, N.; Brinson, M. D.; Daley, M.; Regan, B.; Thomashow, B. | 2017 | abstract only |
| Upper and Lower Respiratory Tract Viral Infections and Acute Graft Rejection in Lung Transplant Recipients | Soccal, P. M.; Aubert, J. D.; Bridevaux, P. O.; Garbino, J.; Thomas, Y.; Rochat, T.; Rochat, T. S.; Meylan, P.; Tapparel, C.; Kaiser, L. | 2010 | wrong population (wrong age group) |
| Epidemiology and clinical impact of viral, atypical, and fungal respiratory pathogens in symptomatic immunocompromised patients: a two-center study using a multi-parameter customized respiratory Taqman array card | Steensels, D.; Reynders, M.; Descheemaeker, P.; Curran, M. D.; Hites, M.; Etienne, I.; Montesinos, I. | 2019 | wrong population (wrong age group) |
| Five-Year Community Surveillance Study for Acute Respiratory Infections Using Text Messaging: Findings from the MoSAIC Study | Stockwell, M. S.; Reed, C.; Vargas, C. Y.; Wang, L.; Alba, L. R.; Jia, H.; Larussa, P.; Larson, E. L.; Saiman, L. | 2022 | wrong outcomes |
| Viral detection in COPD - Implication in exacerbations | Stolz, D.; Hirsch, H.; Schilter, D.; Louis, R.; Rakic, J.; Boeck, L.; Papakonstantinou, E.; Schindler, C.; Grize, L.; Tamm, M. | 2018 | abstract only |
| Medically attended respiratory syncytial virus infections in adults aged >=50 years: Clinical characteristics and outcomes | Sundaram, M. E.; Meece, J. K.; Sifakis, F.; Gasser, R. A.; Belongia, E. A. | 2014 | wrong outcomes |
| Risks and burden of viral respiratory tract infections in patients with multiple myeloma in the era of immunomodulatory drugs and bortezomib: experience at an Australian Cancer Hospital | Teh, B. W.; Worth, L. J.; Harrison, S. J.; Thursky, K. A.; Slavin, M. A. | 2015 | wrong outcomes |
| Epidemiology and outcomes of respiratory virus infections in adult acute leukemia and hematopoietic stem cell transplant patients | Trikha, G.; Conway, R.; Hiemenz, J. W.; Ljungman, P. T. | 2014 | abstract only |
| Surveillance of severe community-acquired pneumonia of unknown etiology-result from unknown pathogen detection/ investigation group (UPDIG), Taiwan | Tsou, T. P.; Chen, W. C.; Hung, M. N.; Wei, S. H.; Lee, H. C.; Su, C. P.; Mu, J. J.; Liu, M. T. | 2017 | abstract only |
| Viral respiratory infections in a nursing home: A six-month prospective study | Ursic, T.; Miksic, N. G.; Lusa, L.; Strle, F.; Petrovec, M. | 2016 | wrong outcomes |
| Respiratory Viruses in Invasively Ventilated Critically Ill Patients-A Prospective Multicenter Observational Study | Van Someren Greve, F.; Juffermans, N. P.; Bos, L. D. J.; Binnekade, J. M.; Braber, A.; Cremer, O. L.; De Jonge, E.; Molenkamp, R.; Ong, D. S. Y.; Rebers, S. P. H.; Spoelstra-De Man, A. M. E.; Van Der Sluijs, K. F.; Spronk, P. E.; Verheul, K. D.; De Waard, M. C.; De Wilde, R. B. P.; Winters, T.; De Jong, M. D.; Schultz, M. J. | 2018 | wrong outcomes |
| Simultaneous Detection of Multiple Respiratory Viruses Among SARS-CoV-2-Positive and Negative Patients by Multiplex TaqMan One-Step Real-Time PCR | Veisi, P.; Malekshahi, S. S.; Choobin, H.; Jabbari, M. R.; Torbati, P. M. | 2022 | wrong outcomes |
| Does detection of respiratory viral infection in upper respiratory tract (URT) predict lower respiratory tract (LRT) disease in hematopoietic cell transplant (HCT) patients? | Vivek, M.; Xie, H.; Pergam, S. A.; Mielcarek, M.; Hill, J.; Kuypers, J.; Jerome, K.; Limaye, A.; Leisenring, W.; Boeckh, M.; Waghmare, A. | 2017 | abstract only |
| Respiratory viral infections among hospitalized adults: Experience of a single tertiary healthcare hospital | Walker, E.; Ison, M. G. | 2014 | wrong or unclear case definition |
| Respiratory virus infection after allogeneic hematopoietic stem cell transplant in a tropical center: Predictive value of the immunodeficiency scoring index | Wang, L.; Allen, J.; Diong, C.; Goh, Y. T.; Gopalakrishnan, S.; Ho, A.; Hwang, W.; Lim, F.; Oon, L.; Tan, T. T.; Linn, Y. C.; Tan, B. H. | 2017 | wrong population (wrong age group) |
| A prospective study comparing human metapneumovirus with other respiratory viruses in adults with hematologic malignancies and respiratory tract infections | Williams, J. V.; Martino, R.; Rabella, N.; Otegui, M.; Parody, R.; Heck, J. M. | 2005 | wrong data collection period |
| Viral respiratory infections after allogeneic hemaotpoietic stem cell transplantation: Outcome and prognosis | Wolfromm, A.; Porcher, R.; Legoff, J.; De Latour, R. P.; Xhaard, A.; Ribaud, P.; Scieux, C.; Bergeron, A.; Socie, G.; Robin, M. | 2011 | abstract only |
| Etiology and clinical characterization of respiratory virus infections in adult patients attending an emergency department in Beijing | Yu, Xiaoyan; Lu, Roujian; Wang, Zhong; Zhu, Na; Wang, Wen; Julian, Druce; Chris, Birch; Lu, Jianxin; Tan, Wenjie | 2012 | wrong population (no comorbidities) |
| Human metapneumovirus infection in adults with lower respiratory tract infections | Zeidan, A.; Zaki, M.; Raafat, D.; Fathy, A. | 2009 | abstract only |
| Disease severity and clinical outcomes of community acquired pneumonia caused by non-influenza respiratory viruses: A multicenter prospective registry study from CAP-China network | Zhou, F.; Wang, Y.; Liu, Y.; Liu, X.; Gu, L.; Zhang, X.; Pu, Z.; Yang, G.; Liu, B.; Mie, Q.; Xue, B.; Feng, J.; Guo, Q.; Wang, C.; Cao, B. | 2019 | abstract only |
| Disease severity and clinical outcomes of community-acquired pneumonia caused by non-influenza respiratory viruses in adults: A multicentre prospective registry study from the CAP-China Network | Zhou, F.; Wang, Y.; Liu, Y.; Liu, X.; Gu, L.; Zhang, X.; Pu, Z.; Yang, G.; Liu, B.; Nie, Q.; Xue, B.; Feng, J.; Guo, Q.; Liu, J.; Fan, H.; Chen, J.; Zhang, Y.; Xu, Z.; Pang, M.; Chen, Y.; Nie, X.; Cai, Z.; Xu, J.; Peng, K.; Li, X.; Xiang, P.; Zhang, Z.; Jiang, S.; Su, X.; Zhang, J.; Li, Y.; Jin, X.; Jiang, R.; Dong, J.; Song, Y.; Zhou, H.; Wang, C.; Cao, B. | 2019 | wrong outcomes |
| Influenza and other respiratory virus infections in outpatients with medically attended acute respiratory infection during the 2011-12 influenza season | Zimmerman, Richard K.; Rinaldo, Charles R.; Nowalk, Mary Patricia; Gk, Balasubramani; Thompson, Mark G.; Moehling, Krissy K.; Bullotta, Arlene; Wisniewski, Stephen | 2014 | wrong outcomes |
| Interferon-alpha Nasal Spray Prophylaxis Reduces COVID-19 in Cancer Patients: A Randomized, Double-Blinded, Placebo-Controlled Trial | Yong, M. K.; Thursky, K.; Crane, M.; Spelman, T.; Mahar, R. K.; Simpson, J. A.; Scott, A. M.; Harrison, S. J.; Szer, J.; Pellegrini, M.; Lingaratnam, S.; Pang, K. C.; Tennakoon, S.; Sim, B. Z.; Blyth, E.; Gan, H. K.; Quach, H.; McIntosh, M. P.; Page, H.; Woolstencroft, R.; Slavin, M. | 2025 | wrong outcomes |
| Incidence and Clinical Outcomes of Multiple Viral Infections After Allogeneic Hematopoietic Cell Transplantation | Yong, K. Y.; Tio, S. Y.; Sim, B. Z.; Sasadeusz, J.; Rivalland, A.; Chee, L.; Szer, J.; Spelman, T.; Slavin, M.; Ritchie, D.; Yong, M. K. | 2025 | wrong population (wrong age group) |
| Incidence and viral aetiology of hospital-acquired respiratory infections at three tertiary care hospitals in Bangladesh, 2008-2011 |  | 2014 | wrong population (no comorbidities) |
| Incidence and Clinical Significance of Respiratory Viral Infections in Patients with Multiple Myeloma A1 - Anonymous |  | 2025 | wrong study design |

# Supplementary Figure S1: Forest plot of random effects meta-analysis estimating the hMPV and RSV positivity in individuals with at least one chronic underlying health condition, stratified by study setting, restricting to studies that collected data over at least one calendar year (annual)


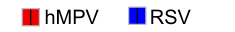


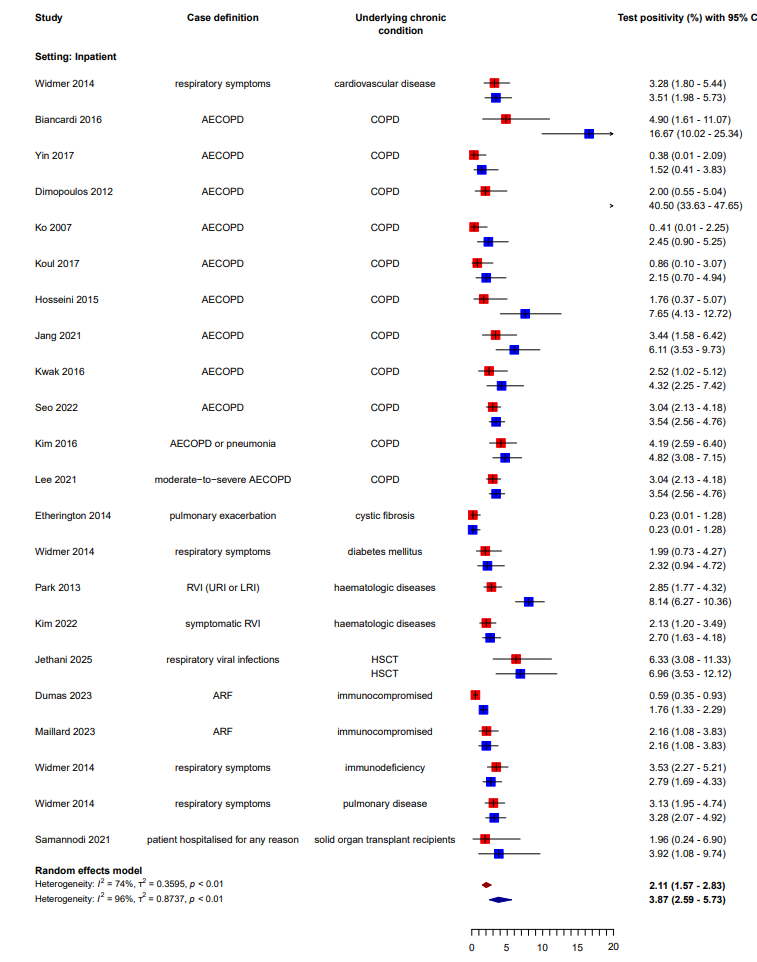


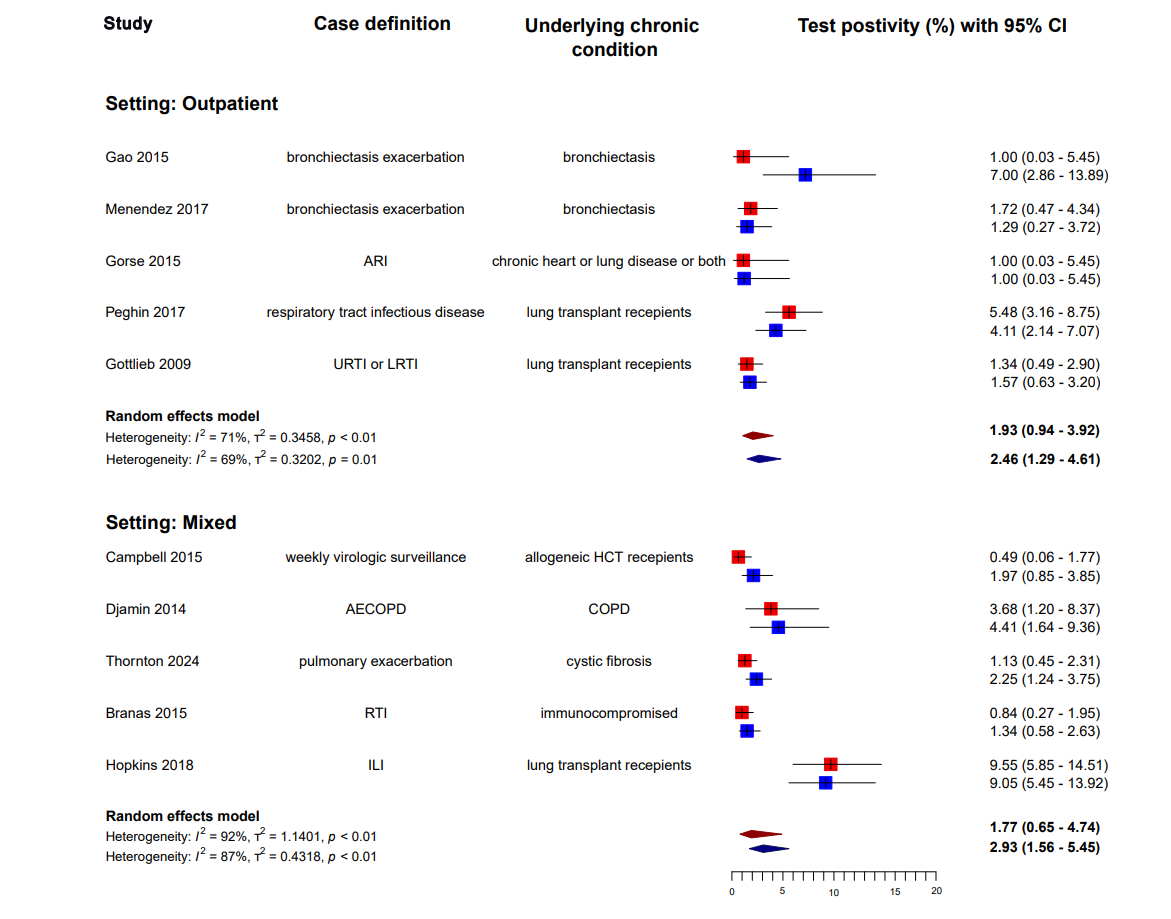


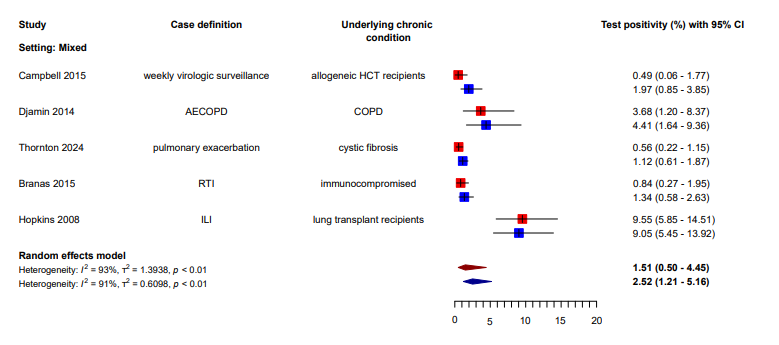


# Supplementary Figure S2: GLMM-predicted proportions of hMPV and RSV positivity in different study settings (population-average estimates) with 95% CI, using annual data


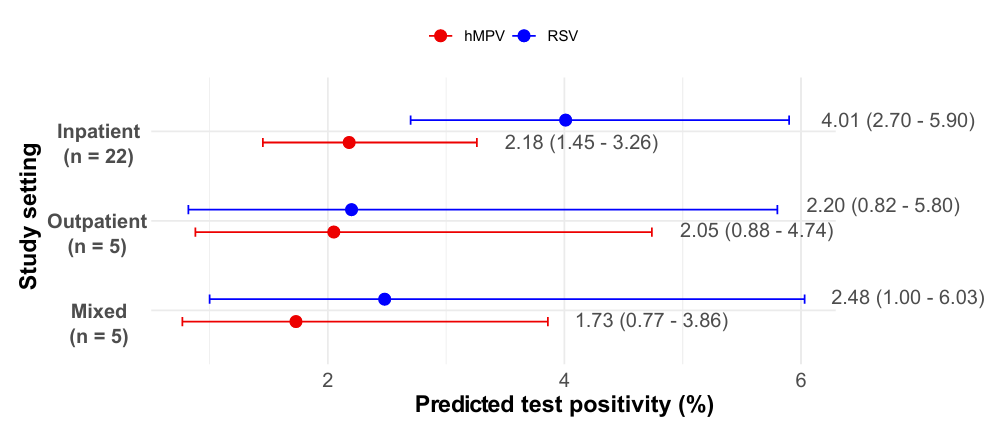


# Supplementary Figure S3: Leave-one-out sensitivity analysis

| 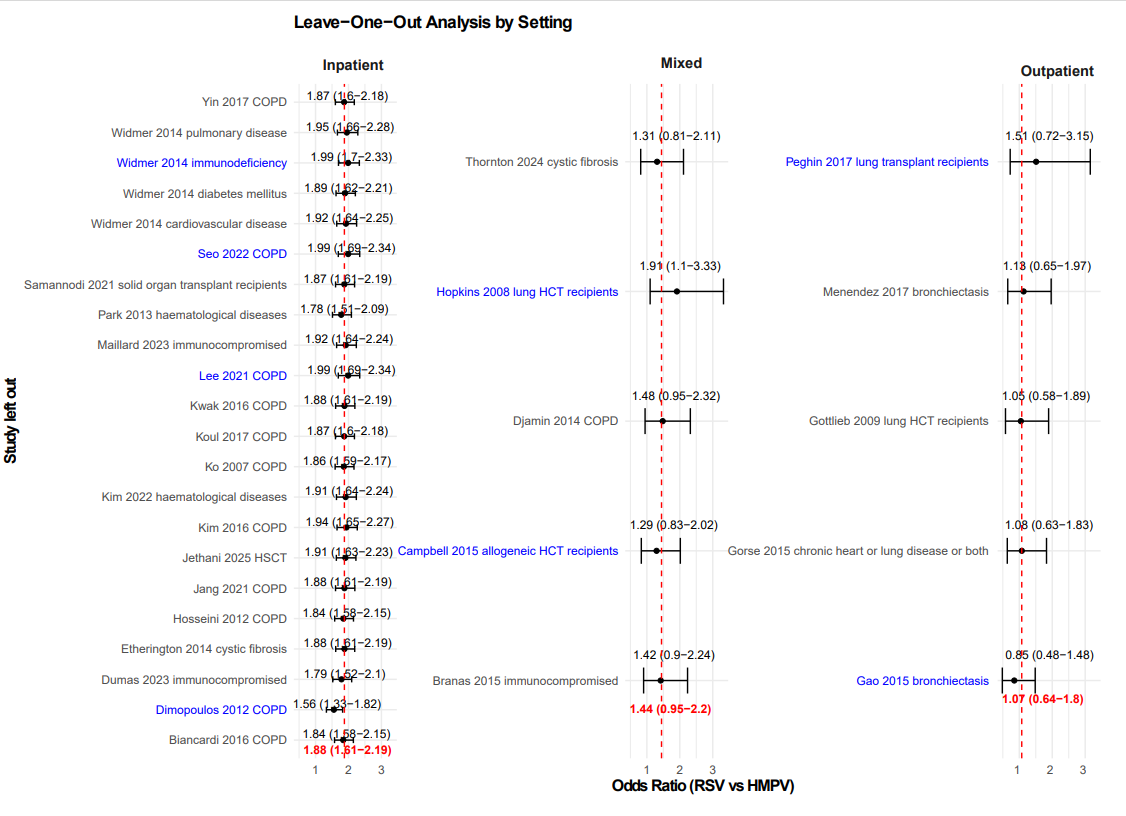 | Black = Leave-one-out estimates,  Red dashed = Full model estimate,  Blue font = Influential estimates |
| --- | --- |

# Supplementary Table S7: Quality appraisal of included studies

## Cohort studies

| First author name | Published year | 1. Were the two groups similar and recruited from the same population? | 2. Were the exposures measured similarly to assign people to both exposed and unexposed groups? | 3. Was the exposure measured in a valid and reliable way? | 4. Were confounding factors identified? | 5. Were strategies to deal with confounding factors stated? | 6. Were the groups/participants free of the outcome at the start of the study (or at the moment of exposure)? | 7. Were the outcomes measured in a valid and reliable way? | 8. Was the follow up time reported and sufficient to be long enough for outcomes to occur? | 9. Was follow up complete, and if not, were the reasons to loss to follow up described and explored? | 10. Were strategies to address incomplete follow up utilized? | 11. Was appropriate statistical analysis used? | Total score |
| --- | --- | --- | --- | --- | --- | --- | --- | --- | --- | --- | --- | --- | --- |
| Akhmedov | 2020 | 0 | 1 | 1 | 1 | 1 | 0 | 1 | 0 | 1 | 1 | 1 | 8 |
| Biancardi | 2016 | 1 | 1 | 1 | 0 | 0 | 0 | 1 | 0 | 0 | 0 | 1 | 5 |
| Branas | 2015 | 0 | 1 | 1 | 0 | 0 | 0 | 1 | 0 | 0 | 0 | 1 | 4 |
| Campbell | 2015 | 1 | 1 | 1 | 1 | 0 | 1 | 1 | 1 | 0 | 0 | 1 | 8 |
| Choi | 2025 | 1 | 1 | 1 | 1 | 0 | 1 | 1 | 1 | 0 | 0 | 1 | 8 |
| Clark | 2015 | 1 | 1 | 1 | 1 | 1 | 1 | 1 | 1 | 1 | 1 | 1 | 11 |
| DeSerres | 2009 | 1 | 1 | 1 | 1 | 0 | 1 | 1 | 0 | 1 | 1 | 1 | 9 |
| Dimopoulos | 2012 | 1 | 1 | 1 | 1 | 0 | 0 | 1 | 1 | 1 | 1 | 1 | 9 |
| Djamin | 2014 | 0 | 1 | 1 | 0 | 0 | 1 | 1 | 1 | 0 | 0 | 1 | 6 |
| Dumas | 2023 | 1 | 0 | 1 | 1 | 1 | 1 | 1 | 0 | 0 | 0 | 1 | 7 |
| Etherington | 2014 | 0 | 1 | 1 | 1 | 0 | 0 | 1 | 0 | 1 | 1 | 1 | 7 |
| Feikin | 2012 | 1 | 1 | 1 | 1 | 1 | 1 | 1 | 1 | 0 | 1 | 1 | 10 |
| Flight | 2014 | 1 | 1 | 1 | 1 | 1 | 1 | 1 | 1 | 1 | 1 | 1 | 11 |
| Gao | 2015 | 1 | 1 | 1 | 1 | 0 | 0 | 1 | 1 | 1 | 0 | 1 | 8 |
| Gorse | 2015 | 1 | 1 | 1 | 1 | 0 | 1 | 1 | 1 | 0 | 0 | 1 | 8 |
| Gottlieb | 2009 | 0 | 0 | 1 | 1 | 1 | 0 | 1 | 1 | 0 | 0 | 1 | 6 |
| Hong | 2014 | 1 | 0 | 1 | 1 | 0 | 1 | 1 | 1 | 1 | 1 | 1 | 9 |
| Hopkins | 2018 | 1 | 0 | 1 | 1 | 1 | 1 | 1 | 0 | 0 | 0 | 1 | 7 |
| Hosseini | 2015 | 1 | 1 | 1 | 1 | 0 | 0 | 1 | 0 | 0 | 0 | 1 | 6 |
| Jang | 2021 | 0 | 1 | 1 | 1 | 1 | 0 | 1 | 0 | 0 | 0 | 1 | 6 |
| Jethani | 2025 | 0 | 1 | 1 | 0 | 0 | 1 | 1 | 1 | 0 | 0 | 1 | 6 |
| Kim | 2016 | 1 | 1 | 1 | 1 | 0 | 1 | 1 | 1 | 0 | 0 | 1 | 8 |
| Kim | 2022 | 0 | 1 | 1 | 1 | 1 | 0 | 1 | 1 | 0 | 0 | 1 | 7 |
| Ko | 2007 | 1 | 1 | 1 | 1 | 0 | 0 | 1 | 1 | 1 | 1 | 1 | 9 |
| Koul | 2017 | 1 | 1 | 1 | 1 | 0 | 0 | 1 | 1 | 0 | 0 | 1 | 7 |
| Kwak | 2016 | 1 | 1 | 1 | 1 | 1 | 0 | 1 | 1 | 0 | 0 | 1 | 8 |
| Lee | 2021 | 1 | 0 | 1 | 1 | 1 | 1 | 1 | 1 | 1 | 1 | 1 | 10 |
| Lokhandwala | 2026 | 1 | 1 | 1 | 1 | 1 | 1 | 1 | 1 | 0 | 0 | 1 | 9 |
| Loubet | 2021 | 1 | 1 | 1 | 1 | 1 | 1 | 1 | 1 | 1 | 1 | 1 | 11 |
| Maillard | 2023 | 1 | 1 | 1 | 1 | 1 | 0 | 1 | 1 | 1 | 0 | 1 | 9 |
| McManus | 2008 | 1 | 1 | 1 | 1 | 0 | 0 | 1 | 1 | 0 | 0 | 1 | 7 |
| Menendez | 2017 | 1 | 1 | 1 | 1 | 1 | 1 | 1 | 1 | 0 | 0 | 1 | 9 |
| Murphy | 2013 | 0 | 1 | 1 | 1 | 1 | 1 | 1 | 0 | 0 | 0 | 1 | 7 |
| Park | 2013 | 1 | 1 | 1 | 1 | 1 | 1 | 1 | 1 | 0 | 0 | 1 | 9 |
| Peghin | 2017 | 1 | 1 | 1 | 1 | 0 | 0 | 1 | 1 | 0 | 0 | 1 | 7 |
| Piñana | 2020 | 1 | 1 | 1 | 1 | 1 | 1 | 1 | 1 | 0 | 0 | 1 | 9 |
| Ponsford | 2021 | 1 | 1 | 1 | 1 | 1 | 0 | 1 | 1 | 0 | 0 | 1 | 8 |
| Ranchow | 2020 | 0 | 1 | 1 | 1 | 1 | 1 | 1 | 1 | 0 | 0 | 1 | 8 |
| Samannodi | 2021 | 1 | 1 | 1 | 1 | 1 | 0 | 1 | 0 | 0 | 0 | 1 | 7 |
| Samoriski | 2025 | 1 | 1 | 1 | 1 | 0 | 0 | 1 | 0 | 0 | 0 | 1 | 6 |
| Seo | 2017 | 1 | 1 | 1 | 1 | 0 | 0 | 1 | 0 | 0 | 0 | 1 | 6 |
| Seo | 2022 | 1 | 1 | 1 | 1 | 1 | 1 | 1 | 1 | 0 | 0 | 1 | 9 |
| Spahr | 2018 | 1 | 1 | 1 | 1 | 1 | 1 | 1 | 0 | 0 | 0 | 1 | 8 |
| Thornton | 2024 | 1 | 1 | 1 | 1 | 0 | 1 | 1 | 1 | 0 | 0 | 1 | 8 |
| Wee | 2025 | 0 | 1 | 1 | 1 | 1 | 1 | 1 | 1 | 0 | 0 | 1 | 8 |
| Weinberg | 2010 | 0 | 1 | 1 | 1 | 0 | 1 | 1 | 1 | 0 | 0 | 1 | 7 |
| Widmer | 2012 | 1 | 1 | 1 | 1 | 0 | 0 | 1 | 1 | 1 | 0 | 1 | 8 |
| Widmer | 2014 | 1 | 1 | 1 | 1 | 0 | 0 | 1 | 1 | 1 | 0 | 1 | 8 |
| Yin | 2017 | 1 | 1 | 1 | 0 | 0 | 0 | 1 | 1 | 1 | 1 | 1 | 8 |

## Cross-sectional studies

| First author | Published year | 1. Were the criteria for inclusion in the sample clearly defined? | 2. Were the study subjects and the setting described in detail? | 3. Was the exposure measured in a valid and reliable way? | 4. Were objective, standard criteria used for measurement of the condition? | 5. Were confounding factors identified | 6. Were strategies to deal with confounding factors stated? | 7. Were the outcomes measured in a valid and reliable way? | 8. Was appropriate statistical analysis used? | Total score |
| --- | --- | --- | --- | --- | --- | --- | --- | --- | --- | --- |
| Reckziegel | 2020 | 1 | 0 | 1 | 1 | 1 | 0 | 1 | 1 | 5 |

# Supplementary Table S8: Sensitivity analysis including good quality studies only

|  | **Main analysis** | | | | **Sensitivity analysis** | | | |
| --- | --- | --- | --- | --- | --- | --- | --- | --- |
| **Setting** | **Number of data points** | **OR (95% CI)** | **z-value** | **p-value** | **Number of data points** | **OR (95% CI)** | **z-value** | **p-value** |
| **Inpatient** | 22 | 1.88 (1.61 – 2.19) | 8.07 | <0.001 | 13 | 1.76 (1.47 – 2.11) | 6.22 | <0.001 |
| **Outpatient** | 5 | 1.07 (0.64 – 1.80) | 0.27 | 0.786 | 3 | 1.86 (0.69 – 4.99) | 1.23 | 0.217 |
| **Mixed** | 5 | 1.44 (0.95 – 2.20) | 1.72 | 0.086 | 2 | 2.47 (1.14 – 5.32) | 2.30 | 0.022 |
| **Overall** | 32 | 1.65 (1.43 – 1.91) | 6.82 | <0.001 | 18 | 1.85 (1.48 – 2.31) | 5.39 | <0.001 |

CI = Confidence interval, OR = Odds ratio

# Supplementary Table S9: Odds of RSV relative to hMPV test positivity in different study settings in adults with at least one chronic underlying condition in high-income countries, using annual data

| **Setting** | **OR (95% CI)** | **z-value** | **p-value** |
| --- | --- | --- | --- |
| **Inpatient (n = 18)** | 1.86 (1.59 – 2.18) | 7.69 | <0.001 |
| **Outpatient (n = 4)** | 0.85 (0.48 – 1.48) | -0.58 | 0.600 |
| **Mixed (n = 5)** | 1.44 (0.95 – 2.20) | 1.72 | 0.086 |
| **Overall (n = 27)** | 1.58 (1.36 – 1.84) | 5.96 | <0.001 |

CI = Confidence Interval, OR = Odds ratio

# Supplementary Figure S4: GLMM-predicted proportions of hMPV and RSV positivity in different study settings in high-income countries (Population-average estimates) with 95% CI, using annual data

**
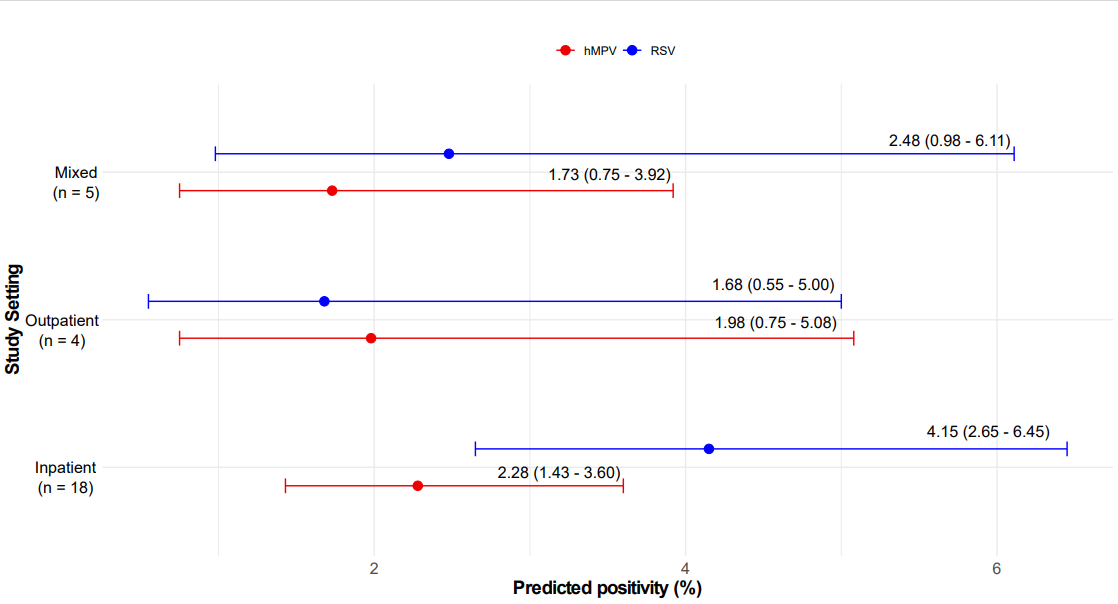
**

# Supplementary Figure S5: GLMM-predicted proportions of hMPV and RSV positivity in different study settings (Population-average estimates) with 95% CI, using seasonal data


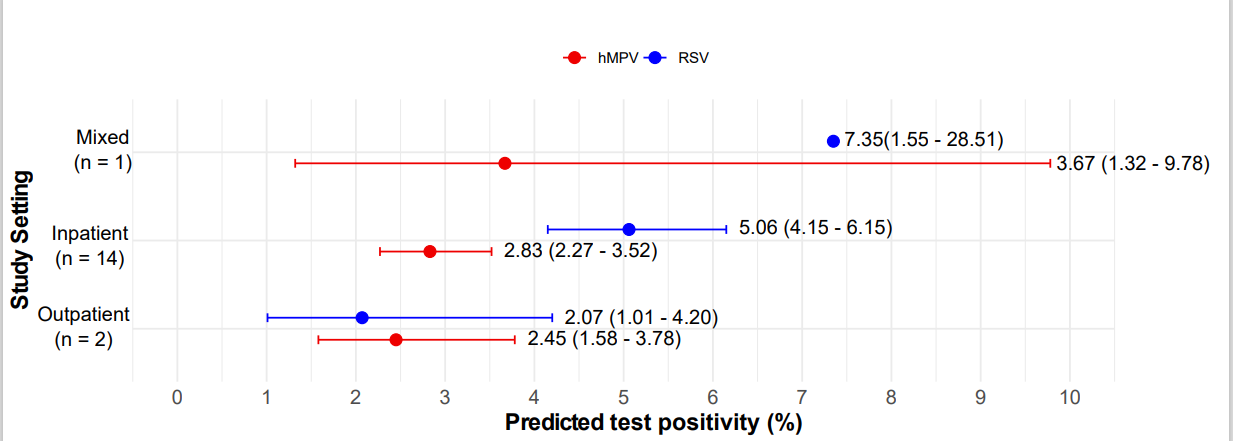


# Supplementary Table S10: Odds of RSV relative to hMPV test positivity in different study settings in adults with at least one chronic underlying health condition, using seasonal data

| **Setting** | **OR (95% CI)** | **z-value** | **p-value** |
| --- | --- | --- | --- |
| **Inpatient (n = 14)** | 1.83 (1.54 – 2.18) | 6.84 | <0.001 |
| **Outpatient (n = 2)** | 0.84 (0.47 – 1.50) | -0.58 | 0.559 |
| **Mixed (n = 1)** | 2.08 (0.61 – 7.13) | 1.17 | 0.243 |
| **Overall (n = 17)** | 1.68 (1.42 – 2.00) | 5.92 | <0.001 |

CI = Confidence interval, OR = Odds ratio

# Supplementary Table S11: hMPV and RSV test positivity reported in studies excluded from the meta-analysis

| **Study** | **Data collection period** | **Setting** | **Case definition** | **Chronic underlying health condition** | **Total episodes tested** | **hMPV- positive episodes** | **RSV-positive episodes** |
| --- | --- | --- | --- | --- | --- | --- | --- |
| Weinberg 2010 | annual | unclear | RTI | lung transplant | 112 | 4 | 8 |
| Reckziegel 2020 | annual | unclear | RTI | immunocompromised | 188 | 2 | 12 |
| Seo 2017 | annual | unclear | suspected RTI (stable LRTI or exacerbated LRTI) | asthma | 323 | 5 | 11 |
| Samoriski 2025 | mixed | inpatient | Acute cardiopulmonary illness or ARI | congestive heart failure | 412 | 16 | 33 |
| Samoriski 2025 | mixed | inpatient | Acute cardiopulmonary illness or ARI | asthma | 591 | 28 | 40 |
| Samoriski 2025 | mixed | inpatient | Acute cardiopulmonary illness or ARI | chronic kidney disease | 130 | 5 | 7 |
| Samoriski 2025 | mixed | inpatient | Acute cardiopulmonary illness or ARI | coronary artery disease | 458 | 24 | 35 |
| Samoriski 2025 | mixed | inpatient | Acute cardiopulmonary illness or ARI | diabetes mellitus | 636 | 32 | 44 |
| Samoriski 2025 | mixed | inpatient | Acute cardiopulmonary illness or ARI | COPD | 781 | 28 | 52 |
| Samoriski 2025 | mixed | inpatient | Acute cardiopulmonary illness or ARI | substance use disorder | 134 | 2 | 9 |
| McManus 2008 | unclear | inpatient | AECOPD | COPD | 136 | 2 | 3 |

hMPV = human metapneumovirus, LRTI = lower respiratory tract infection, RSV = respiratory syncytial virus, RTI = respiratory tract infections

# Supplementary Figure S6: Meta-analysis by chronic underlying condition groups and period of data collection

## Supplementary Figure S6a: Chronic respiratory disease using annual data


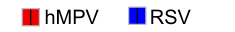


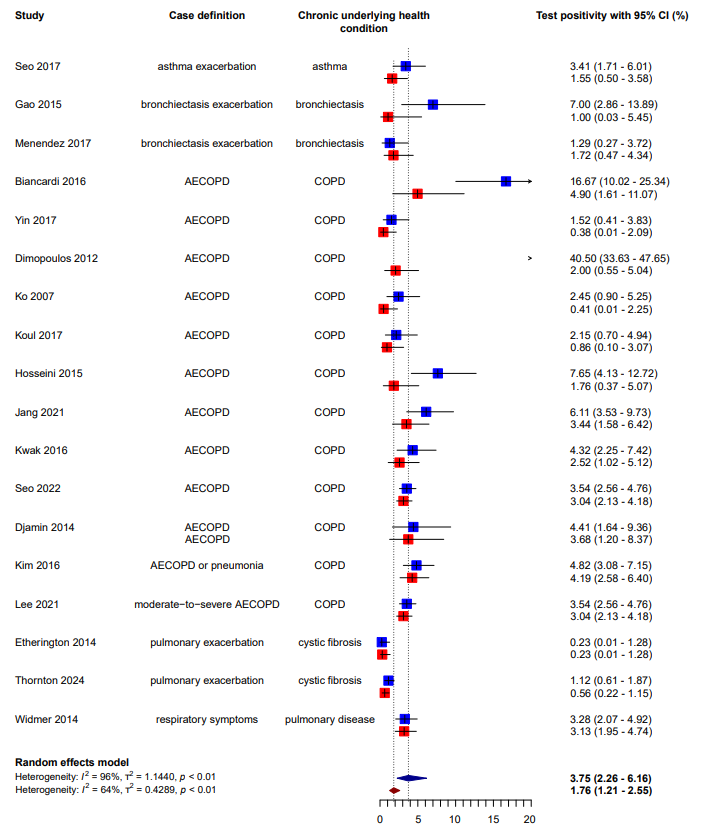


## Supplementary Figure S6b: Chronic respiratory disease using seasonal data


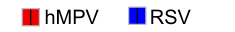


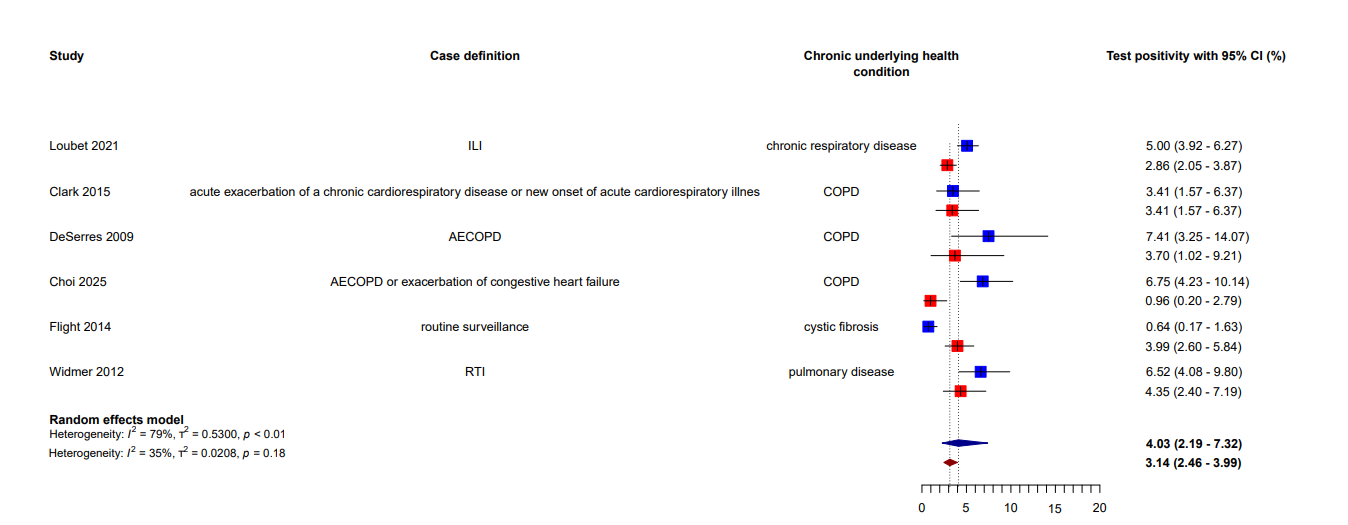


## Supplementary Figure S6c: Cardiovascular disease using seasonal data


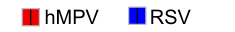


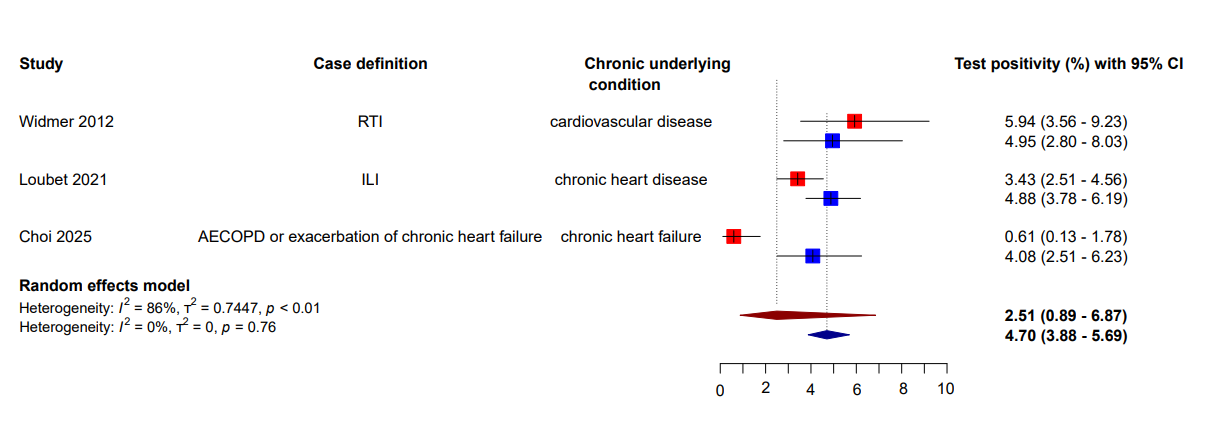


## Supplementary Figure S6d: Organ transplant using annual data


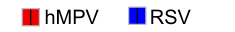


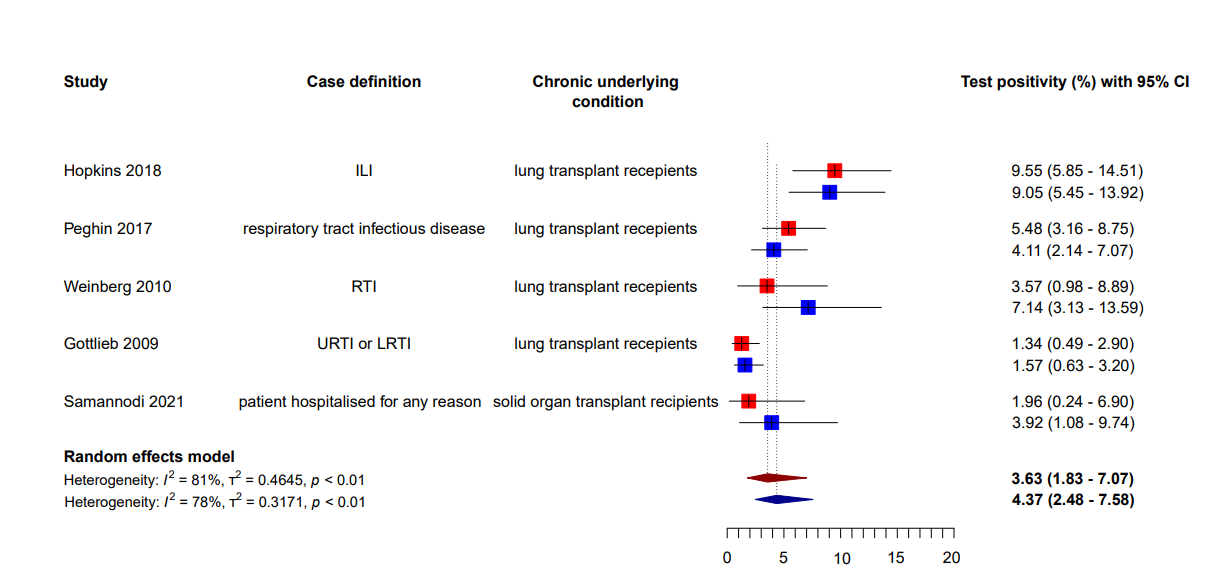


## Supplementary Figure S6e: Immunocompromised status using annual data


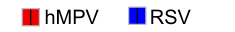


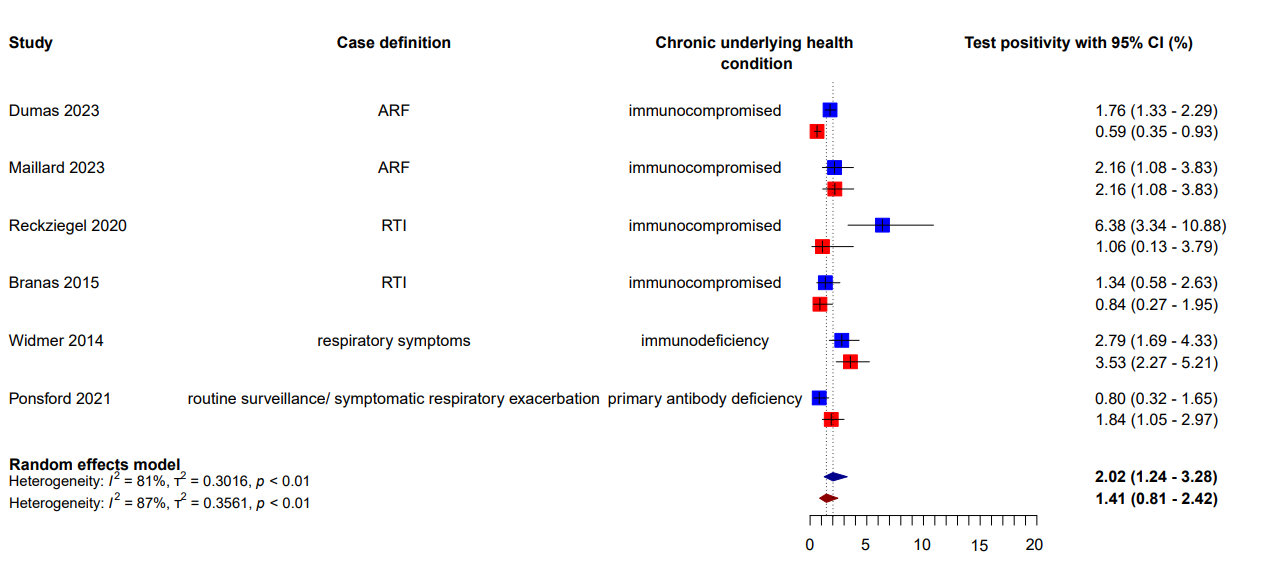


## Supplementary Figure S6f: Haematologic disease using annual data


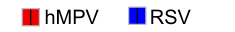


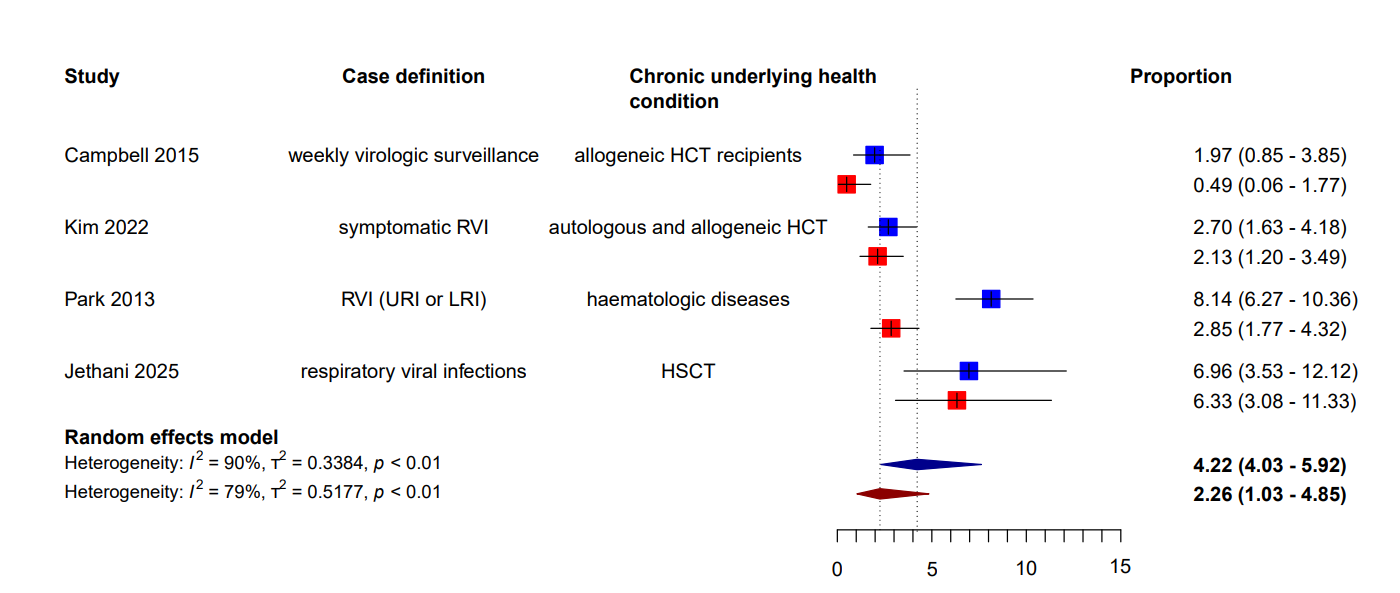


haematologic diseases

# Supplementary Table S12: Findings from studies reporting hMPV and RSV test positivity in adults with COPD, along with other chronic conditions

| **Study** | **Chronic underlying health conditions** | **Total number of episodes tested** | **hMPV- positive episodes** | **RSV-positive episodes** |
| --- | --- | --- | --- | --- |
| Seo 2022 | COPD + interstitial lung disease | 27 | 0 | 1 |
| Seo 2022 | COPD + chronic kidney disease | 76 | 2 | 3 |
| Seo 2022 | COPD + congestive heart failure | 166 | 5 | 12 |
| Seo 2022 | COPD + bronchiectasis | 169 | 2 | 4 |
| Seo 2022 | COPD + diabetes mellitus | 318 | 11 | 6 |
| Seo 2022 | COPD + PTB | 377 | 13 | 14 |
| Lee 2021 | COPD + asthma (ACO) | 144 | 6 | 7 |

ACO = asthma chronic obstructive pulmonary disease overlap, COPD = chronic obstructive pulmonary disease, hMPV = human metapneumovirus, RSV = respiratory syncytial virus, PTB = previous pulmonary tuberculosis

# Supplementary Table S13: Proportion of episodes requiring hospital admission in individual studies

| **Study** | **hMPV-associated hospital admissions/Total hMPV episodes** | **RSV-associated hospital admissions/ Total RSV episodes** | **Case definition** | **Underlying health condition** | **Proportion of hMPV episodes requiring hospital admission with 95% CI (%)** | **Proportion of RSV episodes requiring hospital admission with 95% CI (%)** | **p-value** |
| --- | --- | --- | --- | --- | --- | --- | --- |
| Spahr 2018 | 10/22 | 10/33 | RTID | allogeneic HCT | 45.45 (24.65 – 66.26) | 30.30 (14.62 – 45.98) | 0.270 |
| Weinberg 2010 | 1/4 | 3/8 | RTI | lung transplant | 25.00 (0 – 67.44) | 37.50 (3.95 – 71.05) | 1.000 |

CI = Confidence Interval, HCT = haematopoietic cell transplant, hMPV = human metapneumovirus, RSV = respiratory syncytial virus, RTI = respiratory tract infection, RTID = respiratory tract infectious disease

p-value represents the significance level calculated using Fisher’s exact test.

# Supplementary Table S14: Proportion of episodes requiring intensive care unit (ICU) admission in individual studies

| **Study** | **hMPV-associated ICU admissions/Total hMPV episodes** | **RSV-associated ICU admissions/ Total RSV episodes** | **Case definition** | **Underlying health condition** | **hMPV ICU admission rate with 95% CI (%)** | **RSV ICU admission rate with 95% CI (%)** | **p-value** |
| --- | --- | --- | --- | --- | --- | --- | --- |
| Lokhandwala 2026 | 7/20 | 6/28 | physiologically significant RTI | haematologic malignancies | 35.00 (14.10 – 55.90) | 21.43 (6.23 – 36.63) | 0.339 |
| Spahr 2018 | 1/22 | 2/33 | RTID | HCT | 4.55 (0 - 13.25) | 6.06 (0 - 14.2) | 1.000 |
| Wee 2025 | 12/128 | 27/235 | acute respiratory symptoms | cancer | 9.38 (4.33 – 14.42) | 11.49 (7.41 – 15.57) | 0.598 |

CI = Confidence Interval, HCT = haematopoietic cell transplantation, hMPV = human metapneumovirus, ICU = intensive care unit, RSV = respiratory syncytial virus, RTI = respiratory tract infection, RTID = respiratory tract infectious disease

p-value represents the significance level calculated using Fisher’s exact test.

# Supplementary Table S15: Case fatality rate (CFR) in individual studies

| **Study** | **Number of deaths in hMPV-associated episodes/hMPV episodes** | **Number of deaths in RSV-associated episodes/ RSV episodes** | **Case definition** | **Underlying health condition** | **Mortality outcome** | **hMPV CFR with 95% CI (%)** | **RSV CFR with 95% CI (%)** | **p-value** |
| --- | --- | --- | --- | --- | --- | --- | --- | --- |
| Akhmedov 2020 | 3/7 | 3/23 | LRTI | HCT | virus-associated death | 42.86 (6.20 - 79.52) | 13.04 (0 - 26.81) | 0.120 |
| Dumas 2023 | 3/18 | 14/54 | ARF | immunocompromised | in-hospital death | 16.67 (0 - 33.88) | 25.93 (14.24 - 37.61) | 0.533 |
| Lokhandwala 2026 | 1/20 | 3/28 | physiologically significant RTI | haematologic malignancies | All-cause hospital mortality | 5.00 (0 – 14.55) | 10.71 (0 – 22.17) | 0.632 |
| Park 2013 | 4/21 | 8/60 | RVI (URI or LRI) | haematologic diseases | unclear | 19.05 (2.25 - 35.84) | 13.33 (4.73 - 21.93) | 0.631 |
| Piñana 2020 | 2/17 | 10/46 | LRTD | HCT | 90-day overall mortality | 11.76 (0 - 27.08) | 21.74 (9.82 - 33.66) | 0.487 |
| Spahr 2018 | 2/22 | 2/33 | RTID | HCT | virus-attributable mortality | 9.09 (0 - 21.1) | 6.06 (0 - 14.2) | 1.000 |
| Wee 2025 | 23/128 | 43/235 | acute respiratory symptoms | cancer | all-cause mortality | 17.97 (11.32 – 24.62) | 18.30 (13.35 – 23.24) | 1.000 |

ARF = acute respiratory failure, CI = Confidence Interval, HCT = haematopoietic cell transplantation, hMPV = human metapneumovirus, LRI = lower respiratory infection, LRTD = lower respiratory tract disease, LRTI = lower respiratory tract infection, RSV = respiratory syncytial virus, RTID = respiratory tract infectious disease, RVI = respiratory viral infection, URI = upper respiratory infection

p-value represents the significance level calculated using Fisher’s exact test.
